# DNA Barcoding Works in Practice but Not in (Neutral) Theory

**DOI:** 10.1371/journal.pone.0100755

**Published:** 2014-07-02

**Authors:** Mark Y. Stoeckle, David S. Thaler

**Affiliations:** 1 Program for the Human Environment, The Rockefeller University, New York, New York, United States of America; 2 Department of Earth and Environmental Engineering, Columbia University, New York, New York, United States of America; 3 Department of Pathology and Cell Biology, Columbia University Medical Center, New York, New York, United States of America; Ben-Gurion University of the Negev, Israel

## Abstract

**Background:**

DNA barcode differences within animal species are usually much less than differences among species, making it generally straightforward to match unknowns to a reference library. Here we aim to better understand the evolutionary mechanisms underlying this usual “barcode gap” pattern. We employ avian barcode libraries to test a central prediction of neutral theory, namely, intraspecific variation equals 2 Nµ, where N is population size and µ is mutations per site per generation. Birds are uniquely suited for this task: they have the best-known species limits, are well represented in barcode libraries, and, most critically, are the only large group with documented census population sizes. In addition, we ask if mitochondrial molecular clock measurements conform to neutral theory prediction of clock rate equals µ.

**Results:**

Intraspecific COI barcode variation was uniformly low regardless of census population size (n = 142 species in 15 families). Apparent outliers reflected lumping of reproductively isolated populations or hybrid lineages. Re-analysis of a published survey of cytochrome b variation in diverse birds (n = 93 species in 39 families) further confirmed uniformly low intraspecific variation. Hybridization/gene flow among species/populations was the main limitation to DNA barcode identification.

**Conclusions/Significance:**

To our knowledge, this is the first large study of animal mitochondrial diversity using actual census population sizes and the first to test outliers for population structure. Our finding of universally low intraspecific variation contradicts a central prediction of neutral theory and is not readily accounted for by commonly proposed ad hoc modifications. We argue that the weight of evidence–low intraspecific variation and the molecular clock–indicates neutral evolution plays a minor role in mitochondrial sequence evolution. As an alternate paradigm consistent with empirical data, we propose extreme purifying selection, including at synonymous sites, limits variation within species and continuous adaptive selection drives the molecular clock.

## Introduction

DNA barcoding is a practical method for distinguishing species using a short DNA sequence from a standardized location on the genome. In animals, the agreed-upon standard is a 648 base pair fragment of mitochondrial cytochrome oxidase *c* subunit I (COI) [1], [2]. COI barcode differences within animal species are usually much less than differences among, a pattern often referred to as a “barcode gap,” making it generally straightforward to match unknowns to reference sequences [3]–[5]. A decade of DNA barcoding has generated libraries representing hundreds of thousands of vertebrate and invertebrate species, offering an unprecedented window into genetic variation [6].

To better understand the limits to DNA barcoding and the evolutionary mechanisms that underlie the usual barcode gap pattern, here we use birds to test whether differences within and among species conform to neutral theory, the reigning null hypothesis for mitochondrial sequence evolution [7]–[9]. We analyze apparent barcode gap exceptions in detail–those with unusually large *intra*specific differences and those lacking *inter*specific differences. From a practical point of view exceptions may help define limits to COI barcodes as a marker of speciation. In the context of evolutionary theory, exceptions may give valuable insight into the mechanisms controlling variance within and among species. Birds are uniquely suited this task: they are well represented in barcode libraries, have the best-known species limits of any large animal group, and, most critically, are the only large group with known census population sizes, a key parameter in neutral theory [10]–[13].

Neutral theory posits most sequence differences within and among species are selectively neutral and so invisible to both adaptive and purifying selection. Several lines of evidence support neutrality. First, nearly all mitochondrial protein-coding gene (including COI) differences within and among closely related animal species are synonymous, i.e., do not change the amino acid sequence, indicating the observed changes are at least relatively neutral compared to non-synonymous substitutions [14], [15]. Second, viable hybrids and introgression imply most substitutions are functionally silent rather than species-specific adaptations [16]. Finally, variation in mitochondrial protein-coding region synonymous sites is the same magnitude as in the non-coding control region (e.g., [17]), which is thought to be under highly relaxed selection.

Neutral theory predicts intraspecific variation equals 2 Nµ, where N is population size and µ is mutation rate per generation [7]–[9]. Although textbooks and scientific reports recognize a multitude of exceptions to this predicted relationship, deviations are subsumed under the rubric of “effective population size” and accounted for by ad hoc modifications to the theory, which is assumed operative (e.g., [18]). Here we harness the unique resources of avian barcode libraries and census population data to look at the question the other way around, namely, do the empirical data show any signature of variance proportional to population size? If not, does the observed range of variation fit with commonly proposed modifications to neutral theory? In addition, we examine whether molecular clock measurements conform to neutral theory prediction that clock rate equals µ [19]–[21].

Taxonomic classifications are potential stumbling blocks in analyzing genetic variation. While a DNA sequence is an objective feature of an individual organism, species names reflect expert judgment. Even in a well-studied group like birds, ornithologists describe new species every year by “splitting” taxa previously considered single species [11], [22]. A compilation of DNA sequences may include as yet unsplit species, with the result that variation appears anomalously large. More generally, many species have genetically distinct, geographically isolated subpopulations [23]. Regardless of whether these merit species status, they fit the theoretical model of species as reproductively isolated groups [24], [25]. Another difficulty is hybridization between species or gene flow between regional populations, which may blur both genetic and taxonomic boundaries. Additional sources of inflated variation within species include mislabeled sequence records, sequencing error, and unrecognized pseudogenes [26]. The converse difficulty occurs when species previously considered distinct are “lumped” as single taxa based on new information. In these cases, apparent differences within and among may incorrectly appear low. Here we address potential taxonomic confounders by examining outliers in detail.

In the following we demonstrate uniformly low intraspecific mitochondrial DNA variation in birds regardless of population size. Nearly all apparent exceptions reflect lumping of reproductively isolated populations (many of which represent distinct species) or hybrid lineages. To our knowledge, this is the first large test of neutral theory applied to mitochondrial diversity using actual census population measurements rather than crude proxies of population size such as phylogeny or body weight [27]–[30], and the first to test outliers for population structure. In contrast to prior analyses, we find uniformly low intraspecific variation regardless of census population size. We conclude that this finding together with the molecular clock phenomenon are strong evidence that neutral processes play a minor role in animal mitochondrial evolution. We argue a radically different view of evolution–extreme purifying selection and continuous adaptive evolution–is needed to account for the widespread pattern of limited variation within species and larger differences among that underlies the general effectiveness of DNA barcoding.

## Results

**Figure 1:**
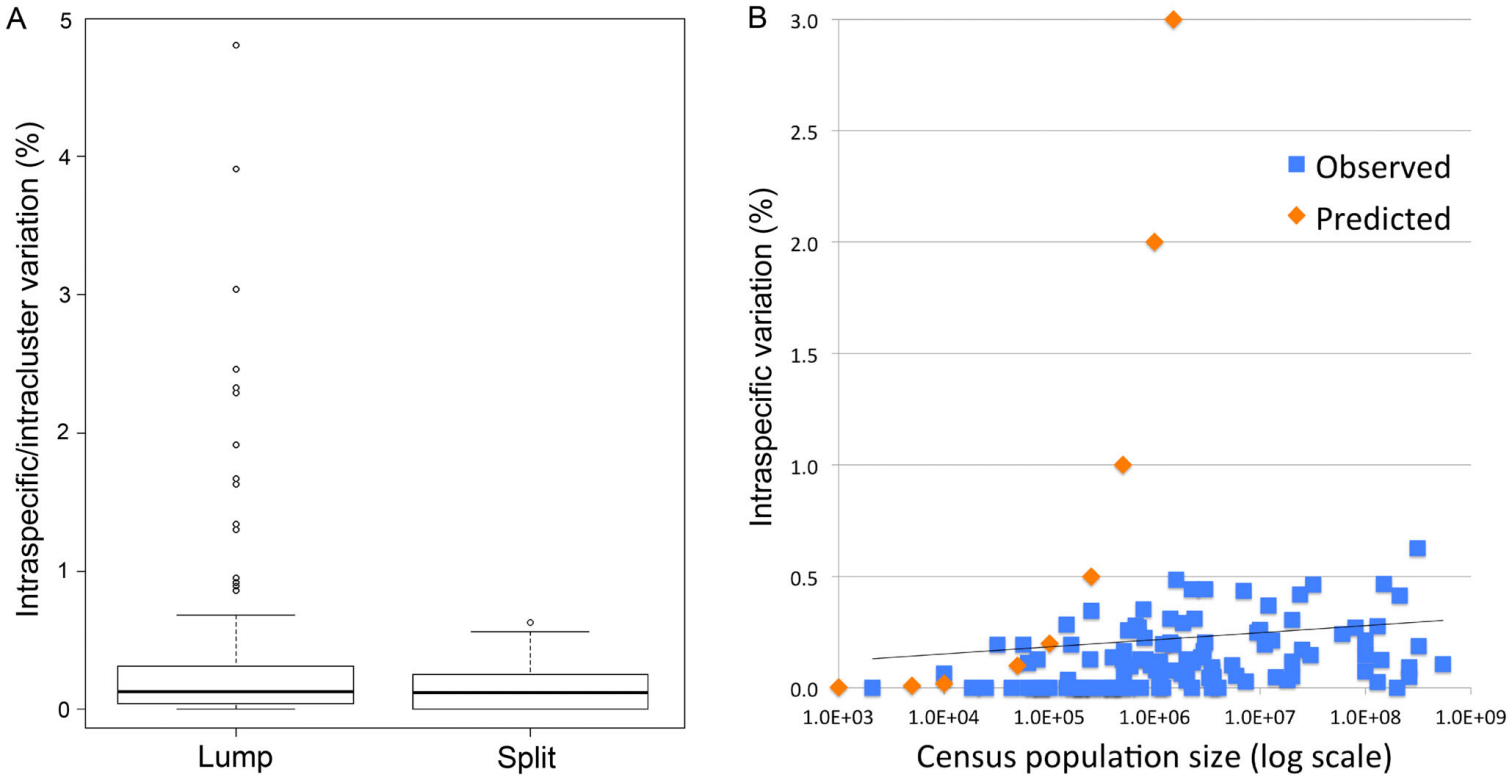
Low ceiling on intraspecific mtDNA variation in birds regardless of population size. A) Intraspecific variation (average pairwise percent K2P difference) in 142 avian species listed in Table S1. Lower and upper bounds of boxes are first and third quartiles respectively, center bar is median, and lower and upper whiskers represent 1.5 times the interquartile range or the minimum or maximum value respectively. Residual values are shown as dots. Right, same dataset except that outliers were separated into geographic or hybrid clusters where present (Table S2). B) Intraspecific variation and population size among 111 species with census estimates; species with geographic or hybrid clusters were excluded. Note y-axis scale differs between A and B. Inclusion of geographic and hybrid clusters as representing equal fractions of parent population gave a similar result. Orange markers indicate predicted variation (2 Nµ) for a model species with census population size N and mutation rate µ of 10^−8^ per nucleotide site per generation (equivalent to 1% per lineage per My).

We examined COI barcode records from two avian families representing the two major divisions of birds, one non-passerine (Scolopacidae, sandpipers; 61 species analyzed) and one passerine (Parulidae, New World warblers; 63 species analyzed). These families were chosen as being among the best characterized in terms of barcode records (number of species and number of individuals per species [26]) and census data [12], [13]. We reasoned that analyzing multiple species within a family would give the clearest evidence for a population size effect since other factors postulated to influence intraspecific variation, such as proportion of breeding adults, number of offspring, generation time, and mating system [7]–[9] are generally similar within avian families [10]. In addition, in case a population size effect was evident only at the extreme of abundance, we analyzed birds with 100 million or more individuals for which 10 or more barcode records were available (18 additional species in 13 additional families). Intraspecific variation measured as average pairwise Kimura-2-parameter (K2P) difference was generally very low (Fig. 1, Table S1) and was unrelated to sample size (Fig. S1). Nearly all cases with high variation, defined as greater than 0.5% average pairwise difference, had geographic or hybrid lineages (Fig. 1, Table S1, Figs. S2, S3, S4, and S5). Geographic lineages were defined as monophyletic branches in NJ trees that mapped to well-established avian biogeographic regions, such as eastern and western North America [25], [31]. In most cases, inferences of reproductive isolation between geographic populations were supported by published studies including analyses using other mitochondrial and nuclear loci (Table S2). In neutral theory a model species is a reproductively isolated population. Thus in terms of variation a reproductively isolated population is expected to behave as an independent entity equivalent to a species. When geographic and hybrid lineages were considered as independent entities, intraspecific diversity appeared tightly constrained with an average about 0.1% and maximum about 0.5% (Fig. 1, Tables S1, S2). The observed average and maximum levels were what would be expected at equilibrium under a neutral model for a species with about 50,000 and 250,000 individuals, respectively [32]. However, our results demonstrated uniformly low variation in birds with census sizes ranging from a few thousand up to several hundred million (Fig. 1).

To test whether these findings were representative of mitochondrial coding region variation in a more diverse set of birds, we re-analyzed a published series reporting up to 5.0% intraspecific variation in avian cytochrome b [30]. Of 92 avian species in 39 families with greater than 0.5% average pairwise difference, review of original reports demonstrated nearly all (92%) reflected geographic clusters (Table S3, Fig. S6). Furthermore, about half (55%) with geographic clusters are currently split or recommended to be split into distinct species. Hybridization and overlooked pseudogenes were present in single cases. The few remaining cases had relatively modest variation (0.5%–1.1%) and small sample sizes (4–6 individuals).

To assess exceptions lacking interspecific differences, we examined all species highlighted as problematic in four recent avian barcode surveys [33]–[36] covering over 1000 species in total. Of 33 pairs or sets of species flagged as difficult to distinguish, four in fact have diagnostic sequence differences, three are proposed to represent single species, and eight lack sufficient data for analysis (single paraphyletic sequences) (Table S4). Of 18 evaluable cases with shared barcode clusters, 14 (78%) are associated with hybridization or introgression.

## Discussion

Our analysis shows uniformly low intraspecific mtDNA variation in birds ranging in population size from 10^3^ to 10^8^ individuals. Apparent outliers demonstrate low variation when reproductively isolated populations and hybridization are taken into account (Fig. 1). This is the first large study of animal mitochondrial diversity that tests for geographic and hybrid lineages and employs actual census population measurements rather than inferring population size from a crude proxy such as phylogeny or body weight [27]–[30]. Our results contrast prior studies in finding variation is uniformly low, rather than differing according to mutation rate [29], [30] or recency of selective sweeps [27]. Re-analysis of a published series [30] further confirmed that outliers reported to have high mitochondrial variation in fact reflect lumping of reproductively isolated populations or overlooked species (Table S3).

### Implications for neutral theory

The universally low intraspecific variation observed in this study does not support a central prediction of neutral theory, namely variance equals 2 Nµ, where N is population size and µ is mutation rate per generation [7]. Taken at face value the results imply µ is inversely proportional to population size across a 100,000-fold range. In contrast, direct measurements of mitochondrial mutation rate per generation are roughly similar in animals analyzed so far including organisms with very different population sizes and generation times such as fruit flies and humans [37].

The ad hoc modifications to neutral theory commonly proposed to account for low variation in individual cases, namely, recurrent bottlenecks or selective sweeps, struggle as general mechanisms. If bottlenecks limit variation, then a universal low ceiling implies recent population crashes for all species. This appears unlikely–almost a Noah’s Ark hypothesis–although perhaps long-term climate cycles might cause widespread periodic bottlenecks [38]. If selective sweeps limit variation, then a universal low ceiling implies a dynamic view of evolution, with all species adapting all the time [39], in contrast to the equilibrium model at the core of neutral theory.

However, even if bottlenecks or selective sweeps do occur regularly in all species, neutral theory still predicts a wide range of variation–with higher levels in organisms that reproduce more rapidly, as the rate of diversity recovery after a selective sweep or bottleneck is proportional to generation time (time to equilibrium variation is N generations) [7], [40]. In contrast to this prediction, the widespread effectiveness of DNA barcoding reflects similarly low levels of intraspecific variation across across the diversity of animal life, including insects and vertebrates that differ 100-fold in generation time (e.g., [41], [42]).

Finally, regardless of selective sweep or bottleneck frequency, there remains a paradox related to the molecular clock. In neutral theory, the molecular clock is proposed to be powered by drift and clock rate equals µ (mutation rate per generation) [19]–[21], [43]. As noted above, µ appears to be approximately the same in different animals. Neutral theory therefore predicts a chronologic clock rate proportional to generation time. However, published studies show the mitochondrial molecular clock ticks at roughly the same rate (∼1–2%/site/My) in diverse animals with very different generation times (e.g., [44], [45]).

Thus two very well documented phenomena, namely, universal limited intraspecific variation and a universal molecular clock, contradict key predictions of neutral theory. We suggest a new paradigm is needed [46]. As a step in that direction, we critically examine two assumptions underlying the neutral model as it is usually applied: most sequence differences are neutral and species are relatively stable over long periods of time, i.e., adaptive evolution is infrequent.

**Figure 2:**
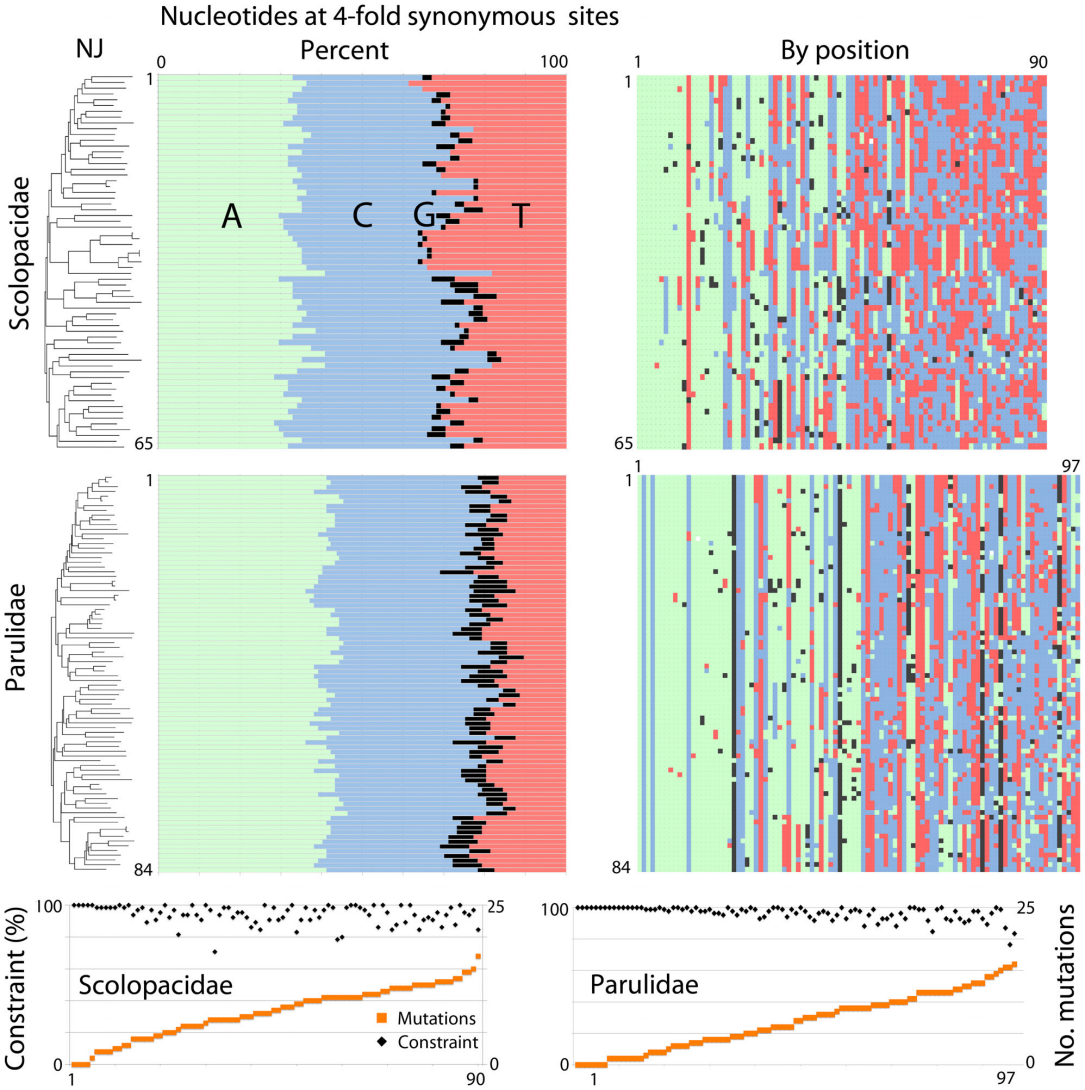
Constrained synonymous variation. From left, COI NJ tree (n = 65 Scolopacidae, 84 Parulidae species), nucleotide composition at four-fold synonymous sites by species (A, adenine, green; C, cytosine, blue; G, guanine, black; T, thymine, red), and by position are shown (n = 90 Scolopacidae, 97 Parulidae sites in 519 nt COI segment). For the latter, positions are sorted by number of apparent mutation events according to NJ tree. At bottom, number of apparent mutation events and percent constraint (limited to one or two nucleotides) for each position are shown. Evidence for restricted variation includes unequal nucleotide composition; unequal distribution of mutation events according to predominant nucleotide (positions with predominantly A have fewer mutation events than those with T or C); and nucleotide composition largely constrained to one or two nucleotides at all sites regardless of number of mutation events.

**Figure 3:**
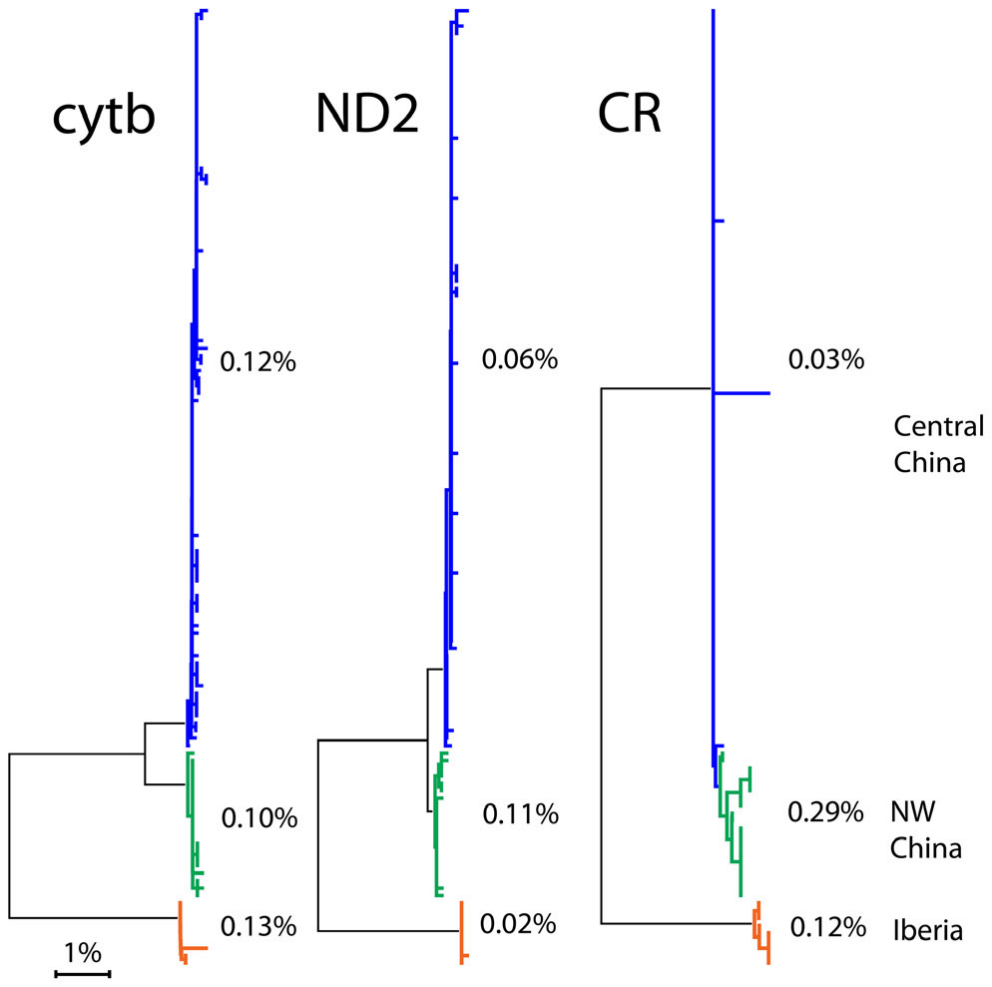
Mitochondrial coding genes and non-coding control region show similar patterns of variation and divergence (adapted from [49]). Azure-winged magpie (*Cyanopica cyanus*) (n = 128 individuals) K2P NJ trees for cytochrome b (cytb), ND2, and control region (CR) generated in MEGA are shown at the same scale. Termini representing geographic populations are colorized and average pairwise percent K2P difference within each geographic population are shown.

Synonymous substitutions are not necessarily selectively silent [47], [48]. As a test we analyzed patterns of interspecific substitution at four-fold synonymous sites, which theoretically can accommodate all four bases. Nucleotide diversity was significantly constrained (Fig. 2). This pattern might indicate translational efficiency limits synonymous substitutions. However, variation appears roughly uniform across mtDNA coding genes and the non-coding control region (e.g., Fig. 3), suggesting a mechanism unrelated to translation limits diversity, although there is much less data on control region than for COI barcodes. Similarly skewed nucleotide composition at four-fold synonymous sites in human and other animal mtDNAs is attributed to strand- and site-specific DNA mutation and repair biases [50]. Regardless of mechanism, an emerging view is exceptions to neutrality in mitochondrial evolution may be the rule [51]–[53].

We hypothesize an absolute limit to intraspecific variation, including at synonymous sites, due to extreme purifying selection. In this scenario, mutations (and reversions) occur at a very small number of positions, resulting in low variation even in large populations over long times. In support, somatic mtDNA mutations in normal human cells usually match variants found in unrelated individuals and rarely involve protein coding regions, suggesting a very limited repertoire of allowable substitutions [54]. In contrast, cancer cells show diverse somatic mutations in coding and non-coding regions that are not represented in mtDNA databases. Further evidence of limited allowable variation is that homoplasy at synonymous sites is common within species (including humans) and between closely-related species [55], [56]. Moreover, much of what appears to be neutral intraspecific variation may instead reflect distinct lineages resulting from local adaptations in heterogeneous environments [48].

Second, we hypothesize species are adapting more or less continuously, with mitochondrial genes evolving together with nuclear genome through genetic draft and epistasis [39], [57], [58]. In this scenario, selective sweeps power the molecular clock [59]. Assuming, as posited above, that allowable variation is tightly restricted, then all species reach the same maximum but low level of variation after a small number of generations, and chronologically-timed selective sweeps produce a chronologic clock.

A universal selection-driven mtDNA clock implies all organisms are evolving at about the same rate [39], [44], [45], [58]. What could cause similar rates of change for diverse organisms in diverse environments? We speculate all life is adapting together at more or less the same rate, tied together by a multitude of food web, predator-prey, and parasite-host interactions, whether on land, lakes, or sea, with long-term planetary climate cycles as the ultimate driver of evolution. This leads us to ask whether there are any environments unchanged over the past hundreds of thousands of years. For example, there might be deep-sea environments with little physical change (although the biological environment might still be dynamic). If so, it would be of interest to examine intraspecific variation and the mitochondrial clock in those settings.

Limitations of this study include most species examined are Nearctic or Palearctic birds. Low intraspecific diversity in these regions may be due to population expansions associated with glacial cycles [60]. However, recent studies of tropical birds show similarly low variation when geographic lineages are taken into account [61], [62]. Another limitation is sample sizes are relatively small. For species with high variation and geographic clustering, the inferred divisions are supported by published work using other mtDNA loci in most cases and correspond to known biogeographic boundaries (Tables S2, S3, Figs. S2, S3, S4, and S5). For species with low variation, further study may reveal greater genetic diversity but this is likely to be in the form of geographic or hybrid lineages.

On the other hand, our results may overestimate variation. Geographic differences were analyzed only in species with greater than 0.5% average or 1.5% maximum variation, and some showed distinct regional clusters below those thresholds. Existing records are insufficient to detect small-scale geographic clustering, particularly as collection locations include migration ranges. Variation in some species may be elevated due to gene flow, i.e., mixing of historically distinct populations.

The findings presented here could in principle be particular to birds. Sexual selection is common in birds and females are the heterogametic sex, with both factors hypothesized to restrict mitochondrial DNA variation [57]. However, barcode surveys abundantly document low intraspecific variance in thousands of species from diverse animal phyla, with exceptions usually carrying biological differences that indicate reproductively isolated populations or overlooked species (e.g., [41], [42]). It may be of interest to conduct similar comprehensive analyses of mtDNA variation in other taxonomically well-studied groups with large DNA barcode databases, such as fish (Actinopterygii) or butterflies and moths (Lepidoptera).

A critical question is whether these findings reflect special features of mitochondrial biology [63]–[65]. A largely unexplored potential mechanism is male contribution to mitochondrial inheritance via gene conversion, which might play a role in limiting variation within species and promoting divergence at speciation. Recent studies call attention to an old riddle of limited intraspecific variation in nuclear genes [66], [67], suggesting the relevant processes are common to both genetic compartments. On the other hand Bazin and colleagues report evidence for neutral evolution in nuclear but not mitochondrial genes [27].

### Implications for DNA barcoding

Based on our analysis, the main limitation to DNA barcode taxonomy is hybridization/gene flow between species/populations that were previously isolated (Table S4). When species or populations with divergent mtDNA genomes interbreed, complex patterns may occur, ranging from occasional hybrid individuals to stable hybrid zones [68] to complete replacement, i.e., introgression [16]. In such cases, assigning individuals to a species with a single mitochondrial locus and even with more extensive mitochondrial and nuclear data may not be possible [69]. From a practical point of view, this scenario appears relatively uncommon, involving a few percent of individuals and species. In fact, the general rarity of large differences within species suggests such reproductive mixing is short-lived from an evolutionary point of view. Such scenarios may similarly challenge taxonomists deciding what constitutes a species vs. a set of populations. Young species per se and retention of ancestral polymorphisms per se do not appear important limitations to DNA barcoding unless there is hybridization or gene flow, since shared polymorphisms are otherwise uncommon. In birds at least, most species with greater than 0.5% variation are composites of reproductively isolated populations, many of which are candidate species. As noted above, deciding whether such populations merit species status necessarily involves expert judgment and additional biological information. In any case, it is evident that the commonly applied 2% threshold is insufficient to capture all animal species [3]. Even single base pair differences may signal reproductively isolated lineages worthy of further study.

### Conclusion

COI barcode variation within avian species is uniformly low regardless of census population size. This finding directly contradicts a central prediction of neutral theory and is not readily accounted for by commonly proposed ad hoc modifications. As an alternative model consistent with empirical data including the molecular clock, we propose extreme purifying selection, including at synonymous sites, limits variation within species and continuous adaptive evolution drives the molecular clock.

## Materials and Methods

COI sequences were downloaded from GenBank using search terms “family or species name”(organism) AND (COI[gene name] OR COX1[gene name]), aligned in MEGA [70] with MUSCLE, and trimmed to 648 bp barcode region corresponding to mouse mitochondrial genome positions 52–699 [2]. Sequences not covering at least 80% of barcode region were omitted. Average and maximum pairwise intraspecific distances were calculated in MEGA after excluding 10% at either end of barcode region (final segment 519 nucleotides, barcode positions 64–582) to minimize contribution of sequencing error [26]. Box and dot plots were generated in R and Excel, respectively. Global population estimates [12], [13] were averaged if given as ranges. To examine nucleotide composition at synonymous sites, representative barcode sequences of Scolopacidae and Parulidae species were aligned and re-ordered using TreeParser following NJ K2P template trees [71]. The re-ordered FASTA files were opened in MEGA and nucleotides at four-fold synonymous sites were exported to Excel for nucleotide composition analysis. To compare coding and control region variation, sequences reported by Zhang and colleagues [49] were downloaded from GenBank and analyzed in MEGA. The analyzed dataset includes cytochrome b, ND2 and control region sequences from 128 Azure-winged Jay (*Cyanopica cyanus*) individuals.

## Supporting Information

Figure S1 **Intraspecific COI barcode variation is unrelated to sample size.**
(PDF)

Figure S2 **Collection locations for Scolopacidae species with high intraspecific variation, geographic clusters.**
(PDF)

Figure S3 **Collection locations for Parulidae species with high intraspecific variation, geographic clusters.**
(PDF)

Figure S4 **Collection locations for highly abundant species with high intraspecific variation, geographic clusters.**
(PDF)

Figure S5 **NJ trees with GenBank accession nos. for birds with high intraspecific variation, geographic clusters.**
(PDF)

Figure S6 **Intraspecific cytochrome b variation is unrelated to sample size (adapted from** [30] **).**
(PDF)

Table S1 **Intraspecific mitochondrial DNA variation in birds is generally low.**
(PDF)

Table S2 **Variation within avian geographic and hybrid clusters is low.**
(PDF)

Table S3 **Re-analysis of intraspecific variation in Nabholz 2009** [30] **avian cytochrome b dataset.**
(PDF)

Table S4 **Avian species with shared DNA barcode clusters.**
(PDF)

References S1 (DOCX)
