# Supplementary material for: DNA Barcoding Works in Practice but Not in (Neutral) Theory
Source: PLoS One. 2014 Jul 2;9(7):e100755. doi: 10.1371/journal.pone.0100755 (PMC4079456; doi:10.1371/journal.pone.0100755)

Fig. S1. Intraspecific COI barcode variation unrelated sample size. Intraspecific variation vs. sample sizes from Tables S1,S2 are shown.

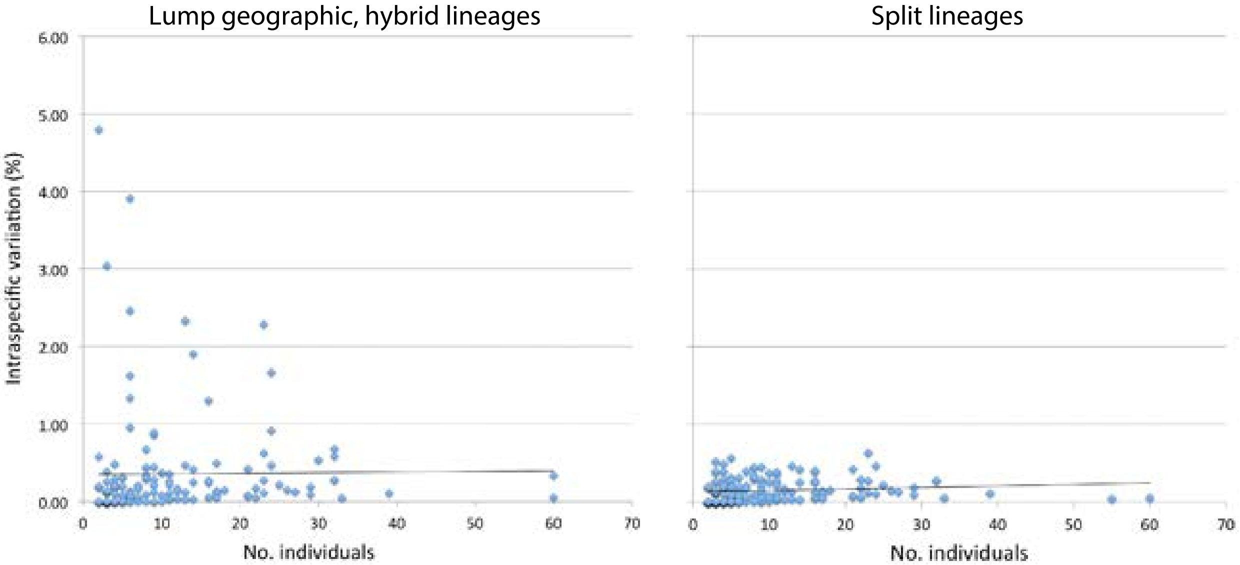

Supplement: Figure S1 — Intraspecific COI barcode variation is unrelated to sample size. (PDF) [file pone.0100755.s001.pdf]
