# Supplementary material for: DNA Barcoding Works in Practice but Not in (Neutral) Theory
Source: PLoS One. 2014 Jul 2;9(7):e100755. doi: 10.1371/journal.pone.0100755 (PMC4079456; doi:10.1371/journal.pone.0100755)

Fig. S2. Collection locations for Scolopacidae species with high variation, geographic clusters. COI NJ trees are shown at left. Collection locations include migration ranges.

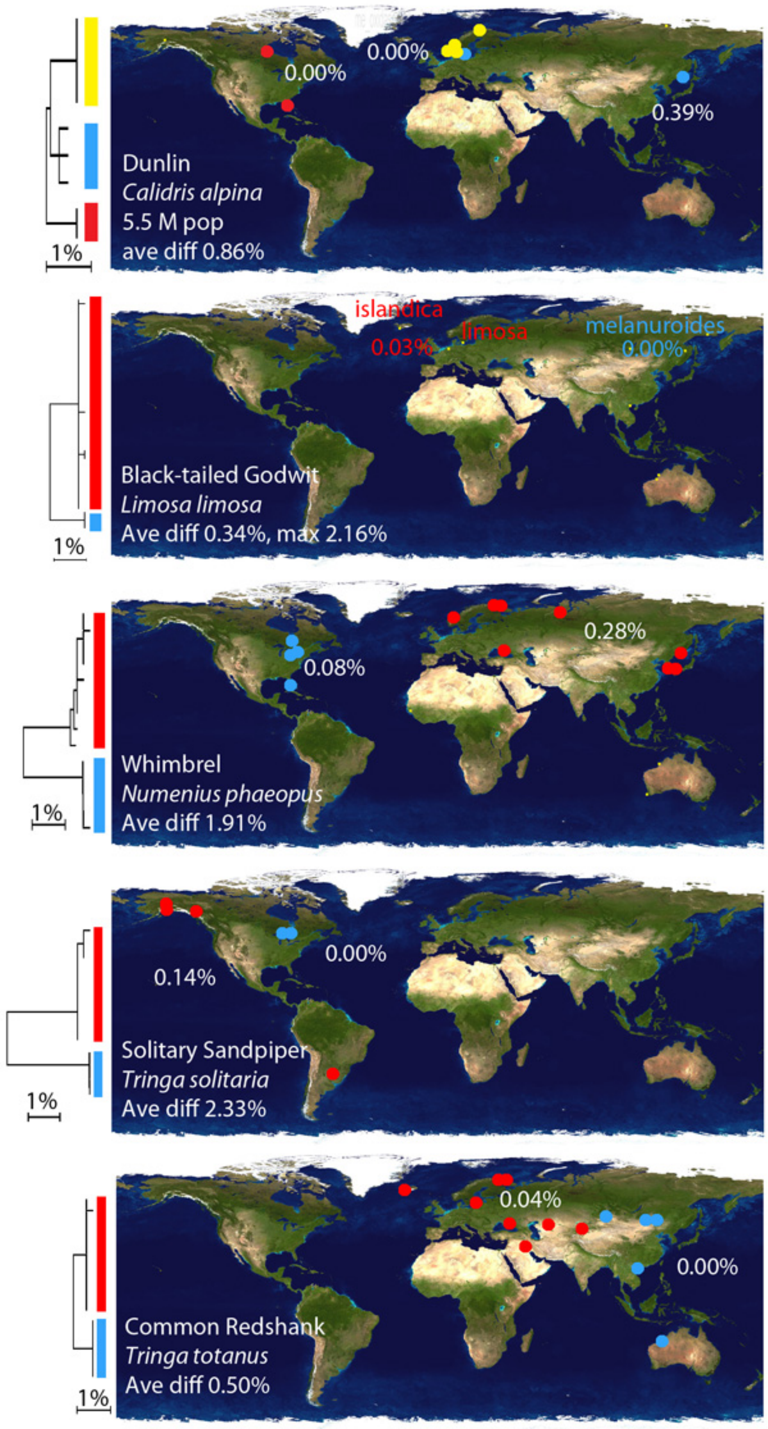

Supplement: Figure S2 — Collection locations for Scolopacidae species with high intraspecific variation, geographic clusters. (PDF) [file pone.0100755.s002.pdf]
