# Supplementary material for: DNA Barcoding Works in Practice but Not in (Neutral) Theory
Source: PLoS One. 2014 Jul 2;9(7):e100755. doi: 10.1371/journal.pone.0100755 (PMC4079456; doi:10.1371/journal.pone.0100755)

**Fig. S3. Collection locations for Parulidae species with high variation, geographic clusters.** COI NJ trees are shown at left. Collection locations include migration ranges. Painted Redstart (*Myioborus pictus*) (n=2, ave diff =0.58%, collection locations Arizona, southern Mexico) not shown.

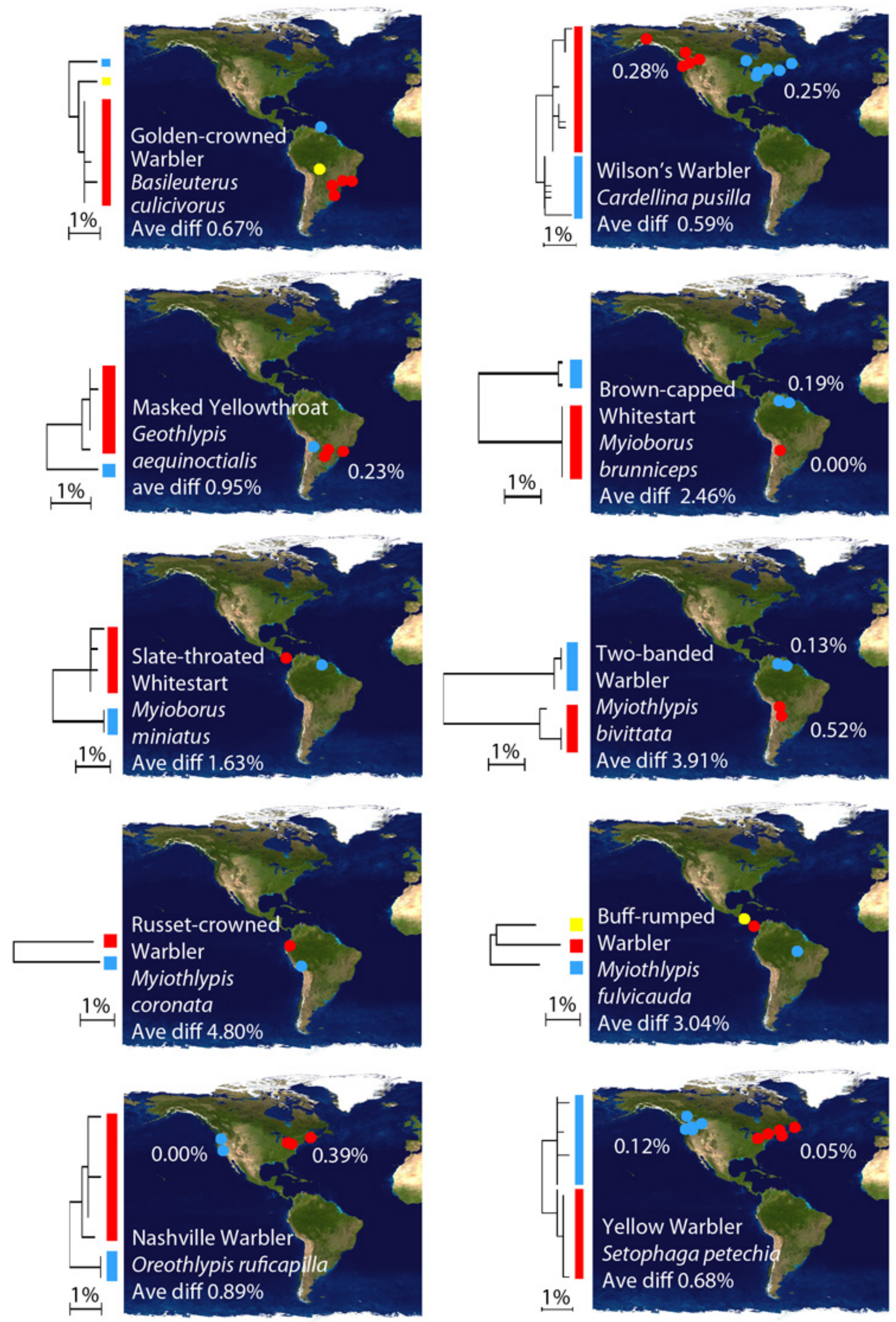

Supplement: Figure S3 — Collection locations for Parulidae species with high intraspecific variation, geographic clusters. (PDF) [file pone.0100755.s003.pdf]
