# Supplementary material for: DNA Barcoding Works in Practice but Not in (Neutral) Theory
Source: PLoS One. 2014 Jul 2;9(7):e100755. doi: 10.1371/journal.pone.0100755 (PMC4079456; doi:10.1371/journal.pone.0100755)

Fig. S4. Collection locations for highly abundant birds with high variation, geographic clusters. COI NJ trees are shown. Collection locations include migration ranges. Collection locations for Tropical Kingbird (*Tyrannus melancholicus*) clusters overlap in eastern Brazil.

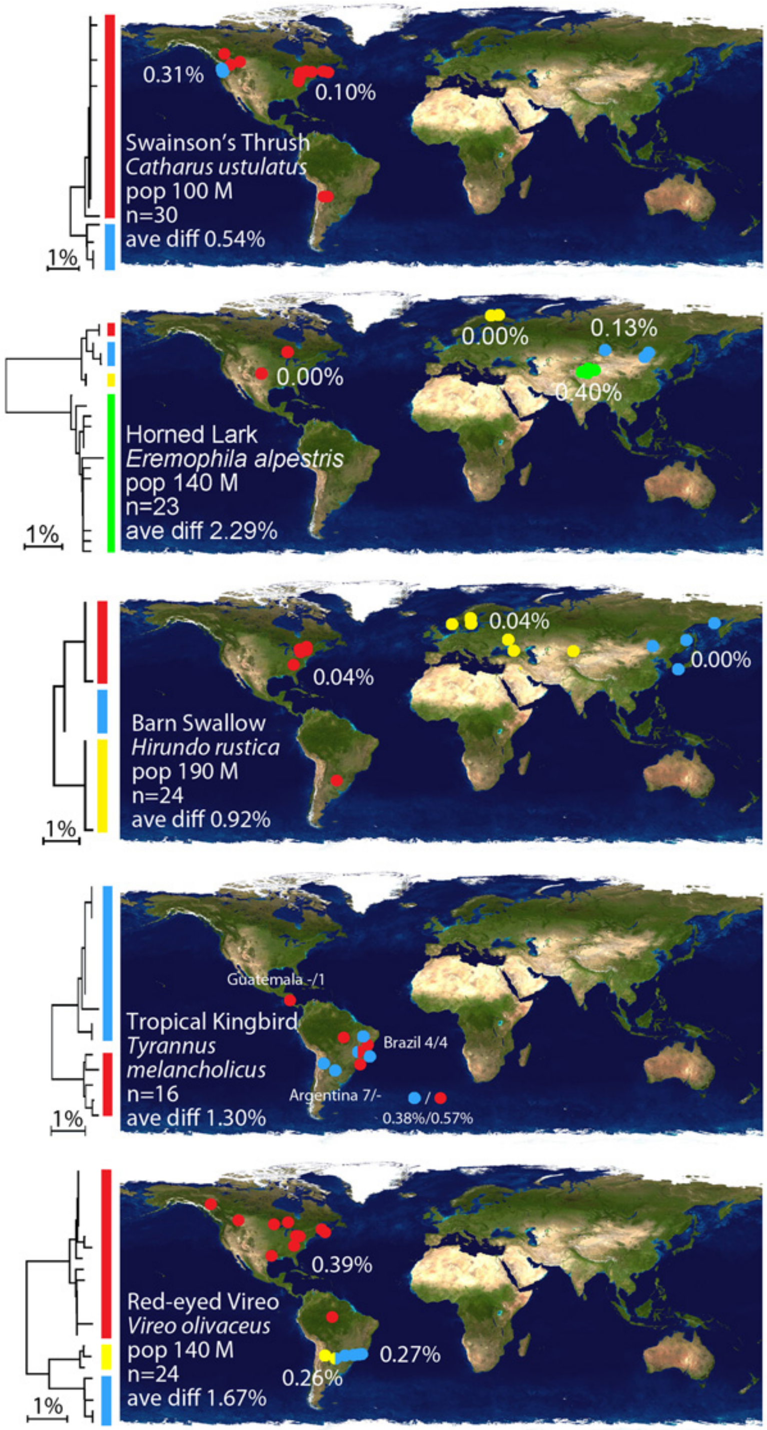

Supplement: Figure S4 — Collection locations for highly abundant species with high intraspecific variation, geographic clusters. (PDF) [file pone.0100755.s004.pdf]
