# Supplementary material for: DNA Barcoding Works in Practice but Not in (Neutral) Theory
Source: PLoS One. 2014 Jul 2;9(7):e100755. doi: 10.1371/journal.pone.0100755 (PMC4079456; doi:10.1371/journal.pone.0100755)
Supplement: Figure S5 — NJ trees with GenBank accession nos. for birds with high intraspecific variation, geographic clusters. (PDF) [file pone.0100755.s005.pdf]

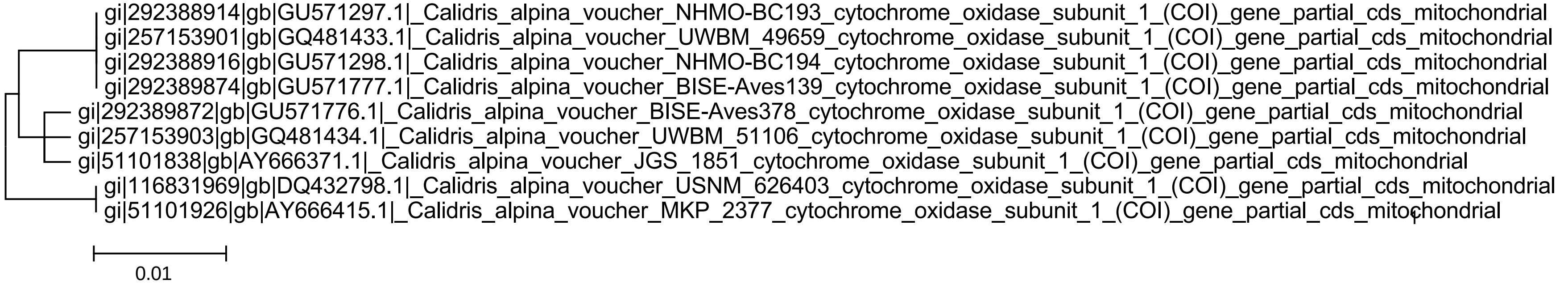
gi|292388914|gb|GU571297.1|_Calidris_alpina_voucher_NHMO-BC193_cytochrome_oxidase_subunit_1_(COI)_gene_partial_cds_mitochondrial
gi|257153901|gb|GQ481433.1|_Calidris_alpina_voucher_UWBM_49659_cytochrome_oxidase_subunit_1_(COI)_gene_partial_cds_mitochondrial
gi|292388916|gb|GU571298.1|_Calidris_alpina_voucher_NHMO-BC194_cytochrome_oxidase_subunit_1_(COI)_gene_partial_cds_mitochondrial
gi|292389874|gb|GU571777.1|_Calidris_alpina_voucher_BISE-Aves139_cytochrome_oxidase_subunit_1_(COI)_gene_partial_cds_mitochondrial
gi|292389872|gb|GU571776.1|_Calidris_alpina_voucher_BISE-Aves378_cytochrome_oxidase_subunit_1_(COI)_gene_partial_cds_mitochondrial
gi|257153903|gb|GQ481434.1|_Calidris_alpina_voucher_UWBM_51106_cytochrome_oxidase_subunit_1_(COI)_gene_partial_cds_mitochondrial
gi|51101838|gb|AY666371.1|_Calidris_alpina_voucher_JGS_1851_cytochrome_oxidase_subunit_1_(COI)_gene_partial_cds_mitochondrial
gi|116831969|gb|DQ432798.1|_Calidris_alpina_voucher_USNM_626403_cytochrome_oxidase_subunit_1_(COI)_gene_partial_cds_mitochondrial
gi|51101926|gb|AY666415.1|_Calidris_alpina_voucher_MKP_2377_cytochrome_oxidase_subunit_1_(COI)_gene_partial_cds_mitochondrial
0.01

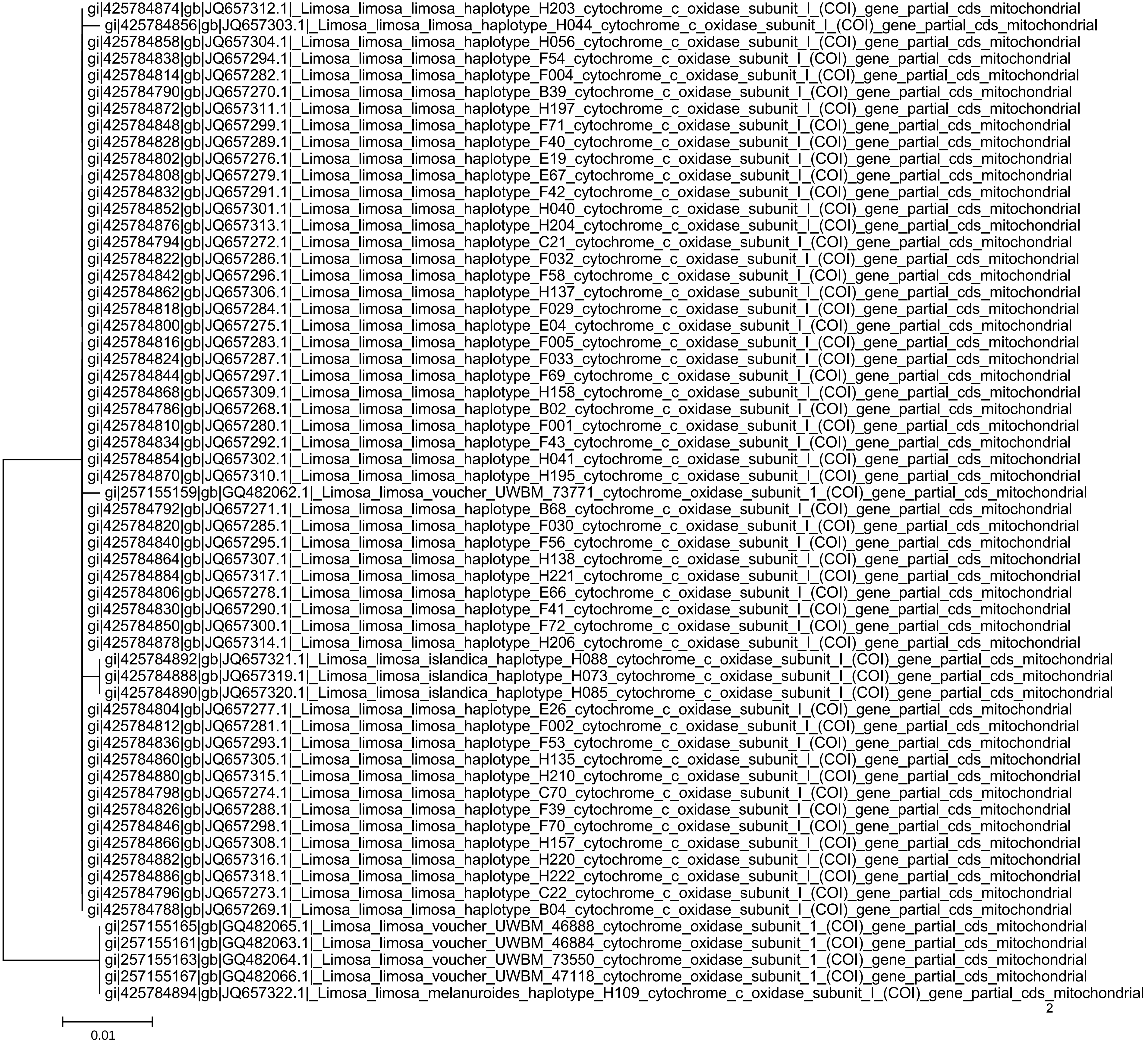
gi|425784874|gb|JQ657312.1|_Limosa_limosa_limosa_haplotype_H203_cytochrome_c_oxidase_subunit_I_(COI)_gene_partial_cds_mitochondrial
gi|425784856|gb|JQ657303.1|_Limosa_limosa_limosa_haplotype_H044_cytochrome_c_oxidase_subunit_I_(COI)_gene_partial_cds_mitochondrial
gi|425784858|gb|JQ657304.1|_Limosa_limosa_limosa_haplotype_H056_cytochrome_c_oxidase_subunit_I_(COI)_gene_partial_cds_mitochondrial
gi|425784838|gb|JQ657294.1|_Limosa_limosa_limosa_haplotype_F54_cytochrome_c_oxidase_subunit_I_(COI)_gene_partial_cds_mitochondrial
gi|425784814|gb|JQ657282.1|_Limosa_limosa_limosa_haplotype_F004_cytochrome_c_oxidase_subunit_I_(COI)_gene_partial_cds_mitochondrial
gi|425784790|gb|JQ657270.1|_Limosa_limosa_limosa_haplotype_B39_cytochrome_c_oxidase_subunit_I_(COI)_gene_partial_cds_mitochondrial
gi|425784872|gb|JQ657311.1|_Limosa_limosa_limosa_haplotype_H197_cytochrome_c_oxidase_subunit_I_(COI)_gene_partial_cds_mitochondrial
gi|425784848|gb|JQ657299.1|_Limosa_limosa_limosa_haplotype_F71_cytochrome_c_oxidase_subunit_I_(COI)_gene_partial_cds_mitochondrial
gi|425784828|gb|JQ657289.1|_Limosa_limosa_limosa_haplotype_F40_cytochrome_c_oxidase_subunit_I_(COI)_gene_partial_cds_mitochondrial
gi|425784802|gb|JQ657276.1|_Limosa_limosa_limosa_haplotype_E19_cytochrome_c_oxidase_subunit_I_(COI)_gene_partial_cds_mitochondrial
gi|425784808|gb|JQ657279.1|_Limosa_limosa_limosa_haplotype_E67_cytochrome_c_oxidase_subunit_I_(COI)_gene_partial_cds_mitochondrial
gi|425784832|gb|JQ657291.1|_Limosa_limosa_limosa_haplotype_F42_cytochrome_c_oxidase_subunit_I_(COI)_gene_partial_cds_mitochondrial
gi|425784852|gb|JQ657301.1|_Limosa_limosa_limosa_haplotype_H040_cytochrome_c_oxidase_subunit_I_(COI)_gene_partial_cds_mitochondrial
gi|425784876|gb|JQ657313.1|_Limosa_limosa_limosa_haplotype_H204_cytochrome_c_oxidase_subunit_I_(COI)_gene_partial_cds_mitochondrial
gi|425784794|gb|JQ657272.1|_Limosa_limosa_limosa_haplotype_C21_cytochrome_c_oxidase_subunit_I_(COI)_gene_partial_cds_mitochondrial
gi|425784822|gb|JQ657286.1|_Limosa_limosa_limosa_haplotype_F032_cytochrome_c_oxidase_subunit_I_(COI)_gene_partial_cds_mitochondrial
gi|425784842|gb|JQ657296.1|_Limosa_limosa_limosa_haplotype_F58_cytochrome_c_oxidase_subunit_I_(COI)_gene_partial_cds_mitochondrial
gi|425784862|gb|JQ657306.1|_Limosa_limosa_limosa_haplotype_H137_cytochrome_c_oxidase_subunit_I_(COI)_gene_partial_cds_mitochondrial
gi|425784818|gb|JQ657284.1|_Limosa_limosa_limosa_haplotype_F029_cytochrome_c_oxidase_subunit_I_(COI)_gene_partial_cds_mitochondrial
gi|425784800|gb|JQ657275.1|_Limosa_limosa_limosa_haplotype_E04_cytochrome_c_oxidase_subunit_I_(COI)_gene_partial_cds_mitochondrial
gi|425784816|gb|JQ657283.1|_Limosa_limosa_limosa_haplotype_F005_cytochrome_c_oxidase_subunit_I_(COI)_gene_partial_cds_mitochondrial
gi|425784824|gb|JQ657287.1|_Limosa_limosa_limosa_haplotype_F033_cytochrome_c_oxidase_subunit_I_(COI)_gene_partial_cds_mitochondrial
gi|425784844|gb|JQ657297.1|_Limosa_limosa_limosa_haplotype_F69_cytochrome_c_oxidase_subunit_I_(COI)_gene_partial_cds_mitochondrial
gi|425784868|gb|JQ657309.1|_Limosa_limosa_limosa_haplotype_H158_cytochrome_c_oxidase_subunit_I_(COI)_gene_partial_cds_mitochondrial
gi|425784786|gb|JQ657268.1|_Limosa_limosa_limosa_haplotype_B02_cytochrome_c_oxidase_subunit_I_(COI)_gene_partial_cds_mitochondrial
gi|425784810|gb|JQ657280.1|_Limosa_limosa_limosa_haplotype_F001_cytochrome_c_oxidase_subunit_I_(COI)_gene_partial_cds_mitochondrial
gi|425784834|gb|JQ657292.1|_Limosa_limosa_limosa_haplotype_F43_cytochrome_c_oxidase_subunit_I_(COI)_gene_partial_cds_mitochondrial
gi|425784854|gb|JQ657302.1|_Limosa_limosa_limosa_haplotype_H041_cytochrome_c_oxidase_subunit_I_(COI)_gene_partial_cds_mitochondrial
gi|425784870|gb|JQ657310.1|_Limosa_limosa_limosa_haplotype_H195_cytochrome_c_oxidase_subunit_I_(COI)_gene_partial_cds_mitochondrial
gi|257155159|gb|GQ482062.1|_Limosa_limosa_voucher_UWBM_73771_cytochrome_oxidase_subunit_1_(COI)_gene_partial_cds_mitochondrial
gi|425784792|gb|JQ657271.1|_Limosa_limosa_limosa_haplotype_B68_cytochrome_c_oxidase_subunit_I_(COI)_gene_partial_cds_mitochondrial
gi|425784820|gb|JQ657285.1|_Limosa_limosa_limosa_haplotype_F030_cytochrome_c_oxidase_subunit_I_(COI)_gene_partial_cds_mitochondrial
gi|425784840|gb|JQ657295.1|_Limosa_limosa_limosa_haplotype_F56_cytochrome_c_oxidase_subunit_I_(COI)_gene_partial_cds_mitochondrial
gi|425784864|gb|JQ657307.1|_Limosa_limosa_limosa_haplotype_H138_cytochrome_c_oxidase_subunit_I_(COI)_gene_partial_cds_mitochondrial
gi|425784884|gb|JQ657317.1|_Limosa_limosa_limosa_haplotype_H221_cytochrome_c_oxidase_subunit_I_(COI)_gene_partial_cds_mitochondrial
gi|425784806|gb|JQ657278.1|_Limosa_limosa_limosa_haplotype_E66_cytochrome_c_oxidase_subunit_I_(COI)_gene_partial_cds_mitochondrial
gi|425784830|gb|JQ657290.1|_Limosa_limosa_limosa_haplotype_F41_cytochrome_c_oxidase_subunit_I_(COI)_gene_partial_cds_mitochondrial
gi|425784850|gb|JQ657300.1|_Limosa_limosa_limosa_haplotype_F72_cytochrome_c_oxidase_subunit_I_(COI)_gene_partial_cds_mitochondrial
gi|425784878|gb|JQ657314.1|_Limosa_limosa_limosa_haplotype_H206_cytochrome_c_oxidase_subunit_I_(COI)_gene_partial_cds_mitochondrial
gi|425784892|gb|JQ657321.1|_Limosa_limosa_islandica_haplotype_H088_cytochrome_c_oxidase_subunit_I_(COI)_gene_partial_cds_mitochondrial
gi|425784888|gb|JQ657319.1|_Limosa_limosa_islandica_haplotype_H073_cytochrome_c_oxidase_subunit_I_(COI)_gene_partial_cds_mitochondrial
gi|425784890|gb|JQ657320.1|_Limosa_limosa_islandica_haplotype_H085_cytochrome_c_oxidase_subunit_I_(COI)_gene_partial_cds_mitochondrial
gi|425784804|gb|JQ657277.1|_Limosa_limosa_limosa_haplotype_E26_cytochrome_c_oxidase_subunit_I_(COI)_gene_partial_cds_mitochondrial
gi|425784812|gb|JQ657281.1|_Limosa_limosa_limosa_haplotype_F002_cytochrome_c_oxidase_subunit_I_(COI)_gene_partial_cds_mitochondrial
gi|425784836|gb|JQ657293.1|_Limosa_limosa_limosa_haplotype_F53_cytochrome_c_oxidase_subunit_I_(COI)_gene_partial_cds_mitochondrial
gi|425784860|gb|JQ657305.1|_Limosa_limosa_limosa_haplotype_H135_cytochrome_c_oxidase_subunit_I_(COI)_gene_partial_cds_mitochondrial
gi|425784880|gb|JQ657315.1|_Limosa_limosa_limosa_haplotype_H210_cytochrome_c_oxidase_subunit_I_(COI)_gene_partial_cds_mitochondrial
gi|425784798|gb|JQ657274.1|_Limosa_limosa_limosa_haplotype_C70_cytochrome_c_oxidase_subunit_I_(COI)_gene_partial_cds_mitochondrial
gi|425784826|gb|JQ657288.1|_Limosa_limosa_limosa_haplotype_F39_cytochrome_c_oxidase_subunit_I_(COI)_gene_partial_cds_mitochondrial
gi|425784846|gb|JQ657298.1|_Limosa_limosa_limosa_haplotype_F70_cytochrome_c_oxidase_subunit_I_(COI)_gene_partial_cds_mitochondrial
gi|425784866|gb|JQ657308.1|_Limosa_limosa_limosa_haplotype_H157_cytochrome_c_oxidase_subunit_I_(COI)_gene_partial_cds_mitochondrial
gi|425784882|gb|JQ657316.1|_Limosa_limosa_limosa_haplotype_H220_cytochrome_c_oxidase_subunit_I_(COI)_gene_partial_cds_mitochondrial
gi|425784886|gb|JQ657318.1|_Limosa_limosa_limosa_haplotype_H222_cytochrome_c_oxidase_subunit_I_(COI)_gene_partial_cds_mitochondrial
gi|425784796|gb|JQ657273.1|_Limosa_limosa_limosa_haplotype_C22_cytochrome_c_oxidase_subunit_I_(COI)_gene_partial_cds_mitochondrial
gi|425784788|gb|JQ657269.1|_Limosa_limosa_limosa_haplotype_B04_cytochrome_c_oxidase_subunit_I_(COI)_gene_partial_cds_mitochondrial
gi|257155165|gb|GQ482065.1|_Limosa_limosa_voucher_UWBM_46888_cytochrome_oxidase_subunit_1_(COI)_gene_partial_cds_mitochondrial
gi|257155161|gb|GQ482063.1|_Limosa_limosa_voucher_UWBM_46884_cytochrome_oxidase_subunit_1_(COI)_gene_partial_cds_mitochondrial
gi|257155163|gb|GQ482064.1|_Limosa_limosa_voucher_UWBM_73550_cytochrome_oxidase_subunit_1_(COI)_gene_partial_cds_mitochondrial
gi|257155167|gb|GQ482066.1|_Limosa_limosa_voucher_UWBM_47118_cytochrome_oxidase_subunit_1_(COI)_gene_partial_cds_mitochondrial
gi|425784894|gb|JQ657322.1|_Limosa_limosa_melanuroides_haplotype_H109_cytochrome_c_oxidase_subunit_I_(COI)_gene_partial_cds_mitochondrial
0.01

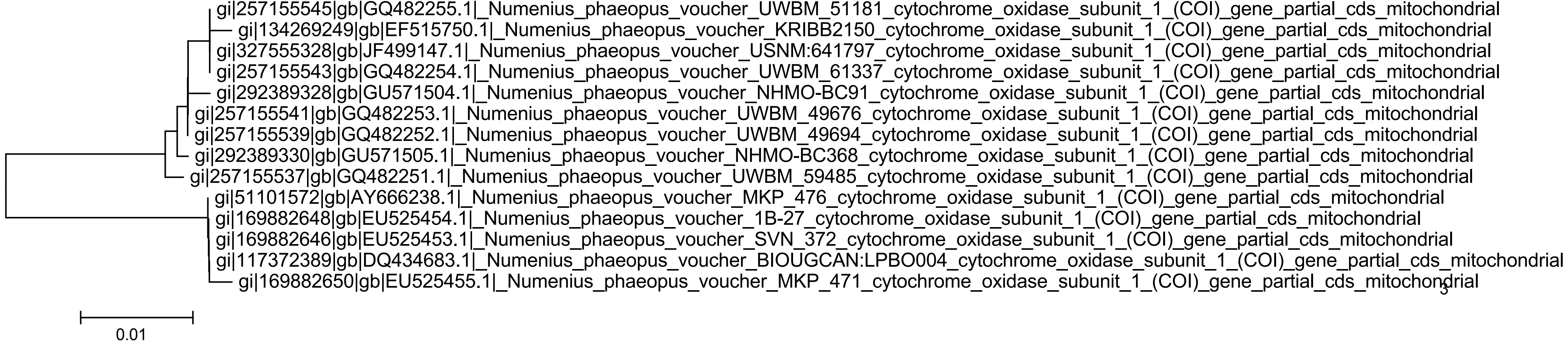
gi|257155545|gb|GQ482255.1|_Numenius_phaeopus_voucher_UWBM_51181_cytochrome_oxidase_subunit_1_(COI)_gene_partial_cds_mitochondrial
gi|134269249|gb|EF515750.1|_Numenius_phaeopus_voucher_KRIBB2150_cytochrome_oxidase_subunit_1_(COI)_gene_partial_cds_mitochondrial
gi|327555328|gb|JF499147.1|_Numenius_phaeopus_voucher_USNM:641797_cytochrome_oxidase_subunit_1_(COI)_gene_partial_cds_mitochondrial
gi|257155543|gb|GQ482254.1|_Numenius_phaeopus_voucher_UWBM_61337_cytochrome_oxidase_subunit_1_(COI)_gene_partial_cds_mitochondrial
gi|292389328|gb|GU571504.1|_Numenius_phaeopus_voucher_NHMO-BC91_cytochrome_oxidase_subunit_1_(COI)_gene_partial_cds_mitochondrial
gi|257155541|gb|GQ482253.1|_Numenius_phaeopus_voucher_UWBM_49676_cytochrome_oxidase_subunit_1_(COI)_gene_partial_cds_mitochondrial
gi|257155539|gb|GQ482252.1|_Numenius_phaeopus_voucher_UWBM_49694_cytochrome_oxidase_subunit_1_(COI)_gene_partial_cds_mitochondrial
gi|292389330|gb|GU571505.1|_Numenius_phaeopus_voucher_NHMO-BC368_cytochrome_oxidase_subunit_1_(COI)_gene_partial_cds_mitochondrial
gi|257155537|gb|GQ482251.1|_Numenius_phaeopus_voucher_UWBM_59485_cytochrome_oxidase_subunit_1_(COI)_gene_partial_cds_mitochondrial
gi|51101572|gb|AY666238.1|_Numenius_phaeopus_voucher_MKP_476_cytochrome_oxidase_subunit_1_(COI)_gene_partial_cds_mitochondrial
gi|169882648|gb|EU525454.1|_Numenius_phaeopus_voucher_1B-27_cytochrome_oxidase_subunit_1_(COI)_gene_partial_cds_mitochondrial
gi|169882646|gb|EU525453.1|_Numenius_phaeopus_voucher_SVN_372_cytochrome_oxidase_subunit_1_(COI)_gene_partial_cds_mitochondrial
gi|117372389|gb|DQ434683.1|_Numenius_phaeopus_voucher_BIOUGCAN:LPBO004_cytochrome_oxidase_subunit_1_(COI)_gene_partial_cds_mitochondrial
gi|169882650|gb|EU525455.1|_Numenius_phaeopus_voucher_MKP_471_cytochrome_oxidase_subunit_1_(COI)_gene_partial_cds_mitochondrial
0.01

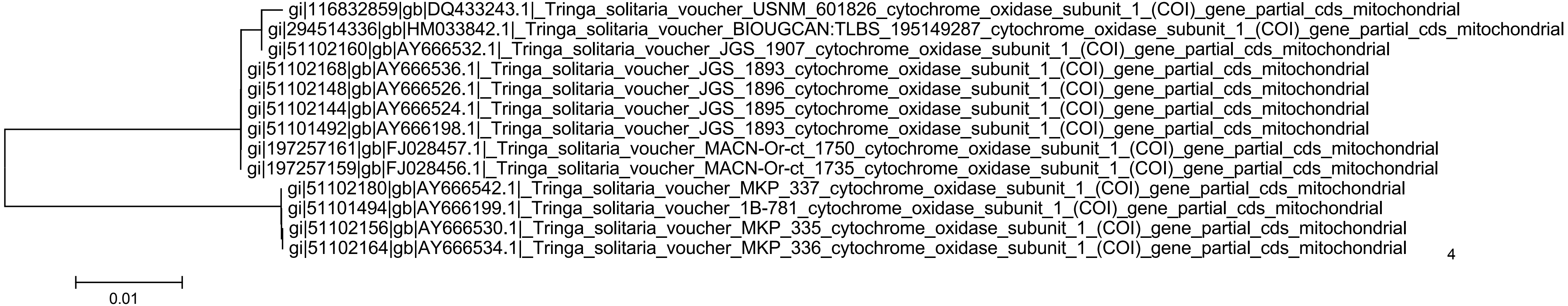
gi|116832859|gb|DQ433243.1|_Tringa_solitaria_voucher_USNM_601826_cytochrome_oxidase_subunit_1_(COI)_gene_partial_cds_mitochondrial
gi|294514336|gb|HM033842.1|_Tringa_solitaria_voucher_BIOUGCAN:TLBS_195149287_cytochrome_oxidase_subunit_1_(COI)_gene_partial_cds_mitochondrial
gi|51102160|gb|AY666532.1|_Tringa_solitaria_voucher_JGS_1907_cytochrome_oxidase_subunit_1_(COI)_gene_partial_cds_mitochondrial
gi|51102168|gb|AY666536.1|_Tringa_solitaria_voucher_JGS_1893_cytochrome_oxidase_subunit_1_(COI)_gene_partial_cds_mitochondrial
gi|51102148|gb|AY666526.1|_Tringa_solitaria_voucher_JGS_1896_cytochrome_oxidase_subunit_1_(COI)_gene_partial_cds_mitochondrial
gi|51102144|gb|AY666524.1|_Tringa_solitaria_voucher_JGS_1895_cytochrome_oxidase_subunit_1_(COI)_gene_partial_cds_mitochondrial
gi|51101492|gb|AY666198.1|_Tringa_solitaria_voucher_JGS_1893_cytochrome_oxidase_subunit_1_(COI)_gene_partial_cds_mitochondrial
gi|197257161|gb|FJ028457.1|_Tringa_solitaria_voucher_MACN-Or-ct_1750_cytochrome_oxidase_subunit_1_(COI)_gene_partial_cds_mitochondrial
gi|197257159|gb|FJ028456.1|_Tringa_solitaria_voucher_MACN-Or-ct_1735_cytochrome_oxidase_subunit_1_(COI)_gene_partial_cds_mitochondrial
gi|51102180|gb|AY666542.1|_Tringa_solitaria_voucher_MKP_337_cytochrome_oxidase_subunit_1_(COI)_gene_partial_cds_mitochondrial
gi|51101494|gb|AY666199.1|_Tringa_solitaria_voucher_1B-781_cytochrome_oxidase_subunit_1_(COI)_gene_partial_cds_mitochondrial
gi|51102156|gb|AY666530.1|_Tringa_solitaria_voucher_MKP_335_cytochrome_oxidase_subunit_1_(COI)_gene_partial_cds_mitochondrial
gi|51102164|gb|AY666534.1|_Tringa_solitaria_voucher_MKP_336_cytochrome_oxidase_subunit_1_(COI)_gene_partial_cds_mitochondrial
0.01

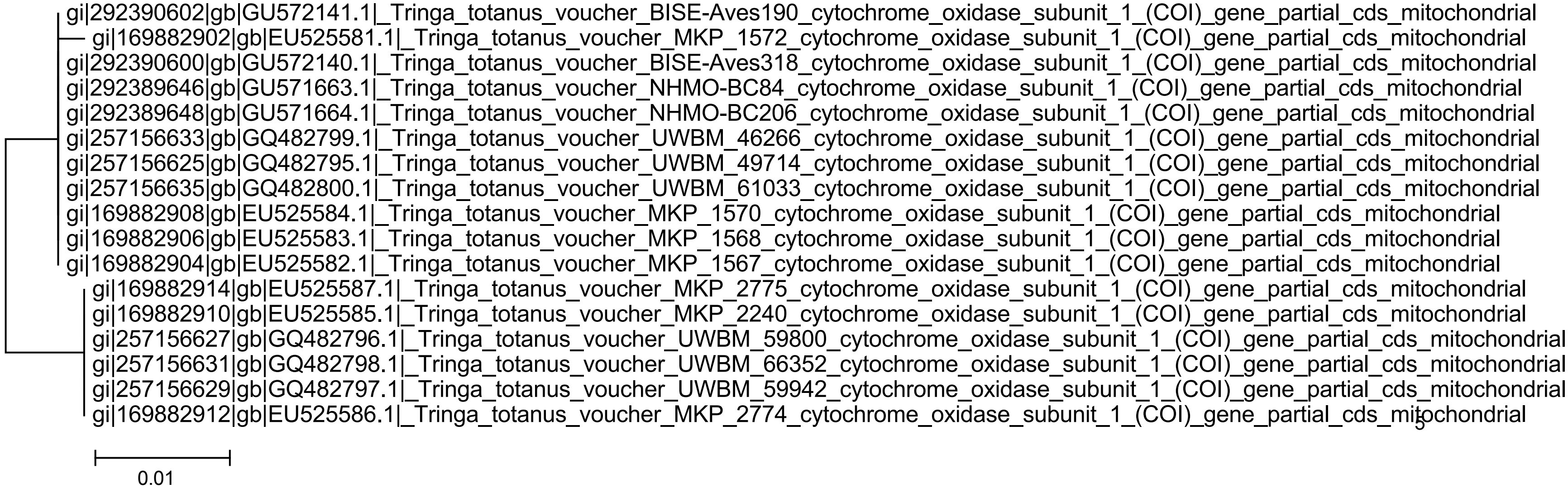
gi|292390602|gb|GU572141.1|_Tringa_totanus_voucher_BISE-Aves190_cytochrome_oxidase_subunit_1_(COI)_gene_partial_cds_mitochondrial
gi|169882902|gb|EU525581.1|_Tringa_totanus_voucher_MKP_1572_cytochrome_oxidase_subunit_1_(COI)_gene_partial_cds_mitochondrial
gi|292390600|gb|GU572140.1|_Tringa_totanus_voucher_BISE-Aves318_cytochrome_oxidase_subunit_1_(COI)_gene_partial_cds_mitochondrial
gi|292389646|gb|GU571663.1|_Tringa_totanus_voucher_NHMO-BC84_cytochrome_oxidase_subunit_1_(COI)_gene_partial_cds_mitochondrial
gi|292389648|gb|GU571664.1|_Tringa_totanus_voucher_NHMO-BC206_cytochrome_oxidase_subunit_1_(COI)_gene_partial_cds_mitochondrial
gi|257156633|gb|GQ482799.1|_Tringa_totanus_voucher_UWBM_46266_cytochrome_oxidase_subunit_1_(COI)_gene_partial_cds_mitochondrial
gi|257156625|gb|GQ482795.1|_Tringa_totanus_voucher_UWBM_49714_cytochrome_oxidase_subunit_1_(COI)_gene_partial_cds_mitochondrial
gi|257156635|gb|GQ482800.1|_Tringa_totanus_voucher_UWBM_61033_cytochrome_oxidase_subunit_1_(COI)_gene_partial_cds_mitochondrial
gi|169882908|gb|EU525584.1|_Tringa_totanus_voucher_MKP_1570_cytochrome_oxidase_subunit_1_(COI)_gene_partial_cds_mitochondrial
gi|169882906|gb|EU525583.1|_Tringa_totanus_voucher_MKP_1568_cytochrome_oxidase_subunit_1_(COI)_gene_partial_cds_mitochondrial
gi|169882904|gb|EU525582.1|_Tringa_totanus_voucher_MKP_1567_cytochrome_oxidase_subunit_1_(COI)_gene_partial_cds_mitochondrial
gi|169882914|gb|EU525587.1|_Tringa_totanus_voucher_MKP_2775_cytochrome_oxidase_subunit_1_(COI)_gene_partial_cds_mitochondrial
gi|169882910|gb|EU525585.1|_Tringa_totanus_voucher_MKP_2240_cytochrome_oxidase_subunit_1_(COI)_gene_partial_cds_mitochondrial
gi|257156627|gb|GQ482796.1|_Tringa_totanus_voucher_UWBM_59800_cytochrome_oxidase_subunit_1_(COI)_gene_partial_cds_mitochondrial
gi|257156631|gb|GQ482798.1|_Tringa_totanus_voucher_UWBM_66352_cytochrome_oxidase_subunit_1_(COI)_gene_partial_cds_mitochondrial
gi|257156629|gb|GQ482797.1|_Tringa_totanus_voucher_UWBM_59942_cytochrome_oxidase_subunit_1_(COI)_gene_partial_cds_mitochondrial
gi|169882912|gb|EU525586.1|_Tringa_totanus_voucher_MKP_2774_cytochrome_oxidase_subunit_1_(COI)_gene_partial_cds_mitochondrial
0.01

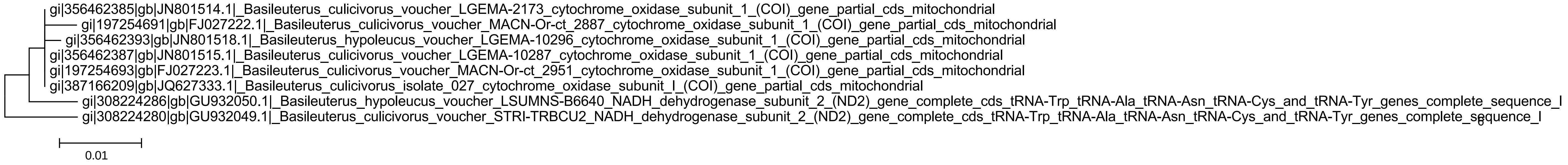
gi|356462385|gb|JN801514.1|_Basileuterus_culicivorus_voucher_LGEMA-2173_cytochrome_oxidase_subunit_1_(COI)_gene_partial_cds_mitochondrial
gi|197254691|gb|FJ027222.1|_Basileuterus_culicivorus_voucher_MACN-Or-ct_2887_cytochrome_oxidase_subunit_1_(COI)_gene_partial_cds_mitochondrial
gi|356462393|gb|JN801518.1|_Basileuterus_hypoleucus_voucher_LGEMA-10296_cytochrome_oxidase_subunit_1_(COI)_gene_partial_cds_mitochondrial
gi|356462387|gb|JN801515.1|_Basileuterus_culicivorus_voucher_LGEMA-10287_cytochrome_oxidase_subunit_1_(COI)_gene_partial_cds_mitochondrial
gi|197254693|gb|FJ027223.1|_Basileuterus_culicivorus_voucher_MACN-Or-ct_2951_cytochrome_oxidase_subunit_1_(COI)_gene_partial_cds_mitochondrial
gi|387166209|gb|JQ627333.1|_Basileuterus_culicivorus_isolate_027_cytochrome_oxidase_subunit_I_(COI)_gene_partial_cds_mitochondrial
gi|308224286|gb|GU932050.1|_Basileuterus_hypoleucus_voucher_LSUMNS-B6640_NADH_dehydrogenase_subunit_2_(ND2)_gene_complete_cds_tRNA-Trp_tRNA-Ala_tRNA-Asn_tRNA-Cys_and_tRNA-Tyr_genes_complete_sequence_I
gi|308224280|gb|GU932049.1|_Basileuterus_culicivorus_voucher_STRI-TRBCU2_NADH_dehydrogenase_subunit_2_(ND2)_gene_complete_cds_tRNA-Trp_tRNA-Ala_tRNA-Asn_tRNA-Cys_and_tRNA-Tyr_genes_complete_sequence_I
0.01

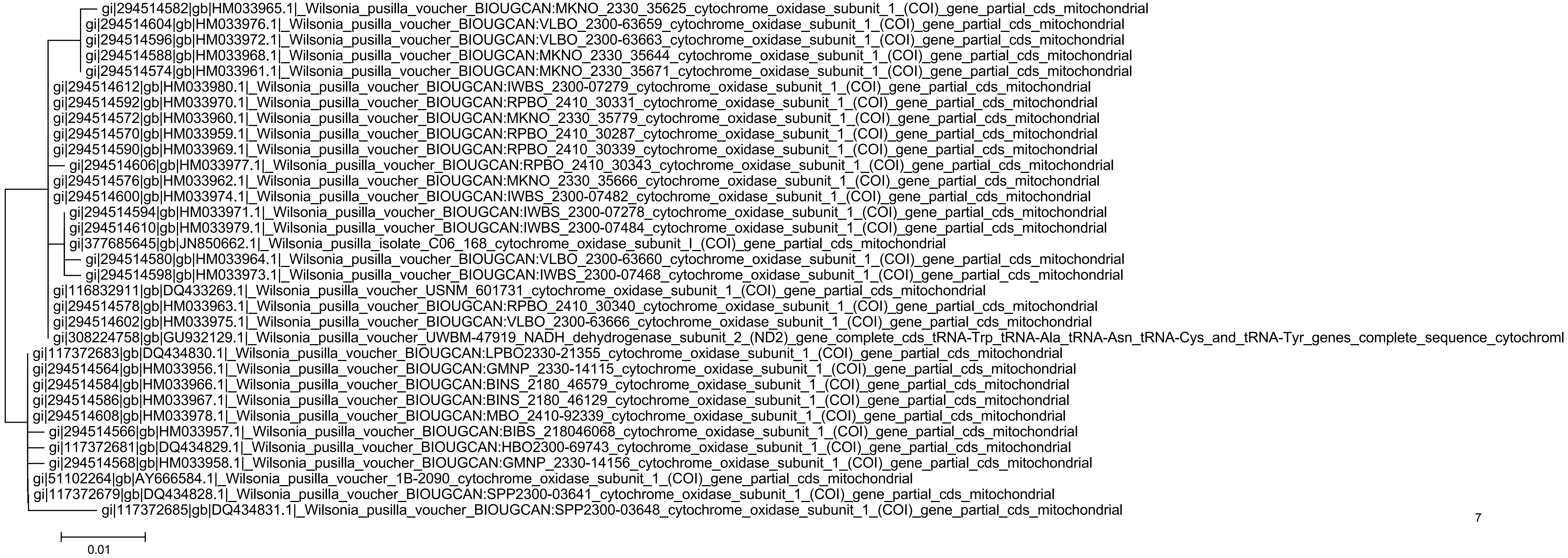
gi|294514582|gb|HM033965.1|_Wilsonia_pusilla_voucher_BIOUGCAN:MKNO_2330_35625_cytochrome_oxidase_subunit_1_(COI)_gene_partial_cds_mitochondrial
gi|294514604|gb|HM033976.1|_Wilsonia_pusilla_voucher_BIOUGCAN:VLBO_2300-63659_cytochrome_oxidase_subunit_1_(COI)_gene_partial_cds_mitochondrial
gi|294514596|gb|HM033972.1|_Wilsonia_pusilla_voucher_BIOUGCAN:VLBO_2300-63663_cytochrome_oxidase_subunit_1_(COI)_gene_partial_cds_mitochondrial
gi|294514588|gb|HM033968.1|_Wilsonia_pusilla_voucher_BIOUGCAN:MKNO_2330_35644_cytochrome_oxidase_subunit_1_(COI)_gene_partial_cds_mitochondrial
gi|294514574|gb|HM033961.1|_Wilsonia_pusilla_voucher_BIOUGCAN:MKNO_2330_35671_cytochrome_oxidase_subunit_1_(COI)_gene_partial_cds_mitochondrial
gi|294514612|gb|HM033980.1|_Wilsonia_pusilla_voucher_BIOUGCAN:IWBS_2300-07279_cytochrome_oxidase_subunit_1_(COI)_gene_partial_cds_mitochondrial
gi|294514592|gb|HM033970.1|_Wilsonia_pusilla_voucher_BIOUGCAN:RPBO_2410_30331_cytochrome_oxidase_subunit_1_(COI)_gene_partial_cds_mitochondrial
gi|294514572|gb|HM033960.1|_Wilsonia_pusilla_voucher_BIOUGCAN:MKNO_2330_35779_cytochrome_oxidase_subunit_1_(COI)_gene_partial_cds_mitochondrial
gi|294514570|gb|HM033959.1|_Wilsonia_pusilla_voucher_BIOUGCAN:RPBO_2410_30287_cytochrome_oxidase_subunit_1_(COI)_gene_partial_cds_mitochondrial
gi|294514590|gb|HM033969.1|_Wilsonia_pusilla_voucher_BIOUGCAN:RPBO_2410_30339_cytochrome_oxidase_subunit_1_(COI)_gene_partial_cds_mitochondrial
gi|294514606|gb|HM033977.1|_Wilsonia_pusilla_voucher_BIOUGCAN:RPBO_2410_30343_cytochrome_oxidase_subunit_1_(COI)_gene_partial_cds_mitochondrial
gi|294514576|gb|HM033962.1|_Wilsonia_pusilla_voucher_BIOUGCAN:MKNO_2330_35666_cytochrome_oxidase_subunit_1_(COI)_gene_partial_cds_mitochondrial
gi|294514600|gb|HM033974.1|_Wilsonia_pusilla_voucher_BIOUGCAN:IWBS_2300-07482_cytochrome_oxidase_subunit_1_(COI)_gene_partial_cds_mitochondrial
gi|294514594|gb|HM033971.1|_Wilsonia_pusilla_voucher_BIOUGCAN:IWBS_2300-07278_cytochrome_oxidase_subunit_1_(COI)_gene_partial_cds_mitochondrial
gi|294514610|gb|HM033979.1|_Wilsonia_pusilla_voucher_BIOUGCAN:IWBS_2300-07484_cytochrome_oxidase_subunit_1_(COI)_gene_partial_cds_mitochondrial
gi|377685645|gb|JN850662.1|_Wilsonia_pusilla_isolate_C06_168_cytochrome_oxidase_subunit_I_(COI)_gene_partial_cds_mitochondrial
gi|294514580|gb|HM033964.1|_Wilsonia_pusilla_voucher_BIOUGCAN:VLBO_2300-63660_cytochrome_oxidase_subunit_1_(COI)_gene_partial_cds_mitochondrial
gi|294514598|gb|HM033973.1|_Wilsonia_pusilla_voucher_BIOUGCAN:IWBS_2300-07468_cytochrome_oxidase_subunit_1_(COI)_gene_partial_cds_mitochondrial
gi|116832911|gb|DQ433269.1|_Wilsonia_pusilla_voucher_USNM_601731_cytochrome_oxidase_subunit_1_(COI)_gene_partial_cds_mitochondrial
gi|294514578|gb|HM033963.1|_Wilsonia_pusilla_voucher_BIOUGCAN:RPBO_2410_30340_cytochrome_oxidase_subunit_1_(COI)_gene_partial_cds_mitochondrial
gi|294514602|gb|HM033975.1|_Wilsonia_pusilla_voucher_BIOUGCAN:VLBO_2300-63666_cytochrome_oxidase_subunit_1_(COI)_gene_partial_cds_mitochondrial
gi|308224758|gb|GU932129.1|_Wilsonia_pusilla_voucher_UWBM-47919_NADH_dehydrogenase_subunit_2_(ND2)_gene_complete_cds_tRNA-Trp_tRNA-Ala_tRNA-Asn_tRNA-Cys_and_tRNA-Tyr_genes_complete_sequence_cytochroml
gi|117372683|gb|DQ434830.1|_Wilsonia_pusilla_voucher_BIOUGCAN:LPBO2330-21355_cytochrome_oxidase_subunit_1_(COI)_gene_partial_cds_mitochondrial
gi|294514564|gb|HM033956.1|_Wilsonia_pusilla_voucher_BIOUGCAN:GMNP_2330-14115_cytochrome_oxidase_subunit_1_(COI)_gene_partial_cds_mitochondrial
gi|294514584|gb|HM033966.1|_Wilsonia_pusilla_voucher_BIOUGCAN:BINS_2180_46579_cytochrome_oxidase_subunit_1_(COI)_gene_partial_cds_mitochondrial
gi|294514586|gb|HM033967.1|_Wilsonia_pusilla_voucher_BIOUGCAN:BINS_2180_46129_cytochrome_oxidase_subunit_1_(COI)_gene_partial_cds_mitochondrial
gi|294514608|gb|HM033978.1|_Wilsonia_pusilla_voucher_BIOUGCAN:MBO_2410-92339_cytochrome_oxidase_subunit_1_(COI)_gene_partial_cds_mitochondrial
gi|294514566|gb|HM033957.1|_Wilsonia_pusilla_voucher_BIOUGCAN:BIBS_218046068_cytochrome_oxidase_subunit_1_(COI)_gene_partial_cds_mitochondrial
gi|117372681|gb|DQ434829.1|_Wilsonia_pusilla_voucher_BIOUGCAN:HBO2300-69743_cytochrome_oxidase_subunit_1_(COI)_gene_partial_cds_mitochondrial
gi|294514568|gb|HM033958.1|_Wilsonia_pusilla_voucher_BIOUGCAN:GMNP_2330-14156_cytochrome_oxidase_subunit_1_(COI)_gene_partial_cds_mitochondrial
gi|51102264|gb|AY666584.1|_Wilsonia_pusilla_voucher_1B-2090_cytochrome_oxidase_subunit_1_(COI)_gene_partial_cds_mitochondrial
gi|117372679|gb|DQ434828.1|_Wilsonia_pusilla_voucher_BIOUGCAN:SPP2300-03641_cytochrome_oxidase_subunit_1_(COI)_gene_partial_cds_mitochondrial
gi|117372685|gb|DQ434831.1|_Wilsonia_pusilla_voucher_BIOUGCAN:SPP2300-03648_cytochrome_oxidase_subunit_1_(COI)_gene_partial_cds_mitochondrial
0.01

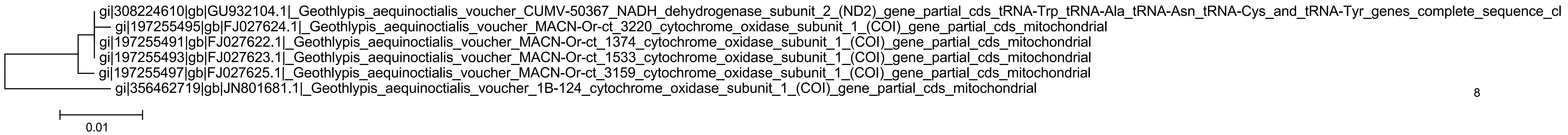
gi|308224610|gb|GU932104.1|_Geothlypis_aequinoctialis_voucher_CUMV-50367_NADH_dehydrogenase_subunit_2_(ND2)_gene_partial_cds_tRNA-Trp_tRNA-Ala_tRNA-Asn_tRNA-Cys_and_tRNA-Tyr_genes_complete_sequence_cl
gi|197255495|gb|FJ027624.1|_Geothlypis_aequinoctialis_voucher_MACN-Or-ct_3220_cytochrome_oxidase_subunit_1_(COI)_gene_partial_cds_mitochondrial
gi|197255491|gb|FJ027622.1|_Geothlypis_aequinoctialis_voucher_MACN-Or-ct_1374_cytochrome_oxidase_subunit_1_(COI)_gene_partial_cds_mitochondrial
gi|197255493|gb|FJ027623.1|_Geothlypis_aequinoctialis_voucher_MACN-Or-ct_1533_cytochrome_oxidase_subunit_1_(COI)_gene_partial_cds_mitochondrial
gi|197255497|gb|FJ027625.1|_Geothlypis_aequinoctialis_voucher_MACN-Or-ct_3159_cytochrome_oxidase_subunit_1_(COI)_gene_partial_cds_mitochondrial
gi|356462719|gb|JN801681.1|_Geothlypis_aequinoctialis_voucher_1B-124_cytochrome_oxidase_subunit_1_(COI)_gene_partial_cds_mitochondrial
0.01

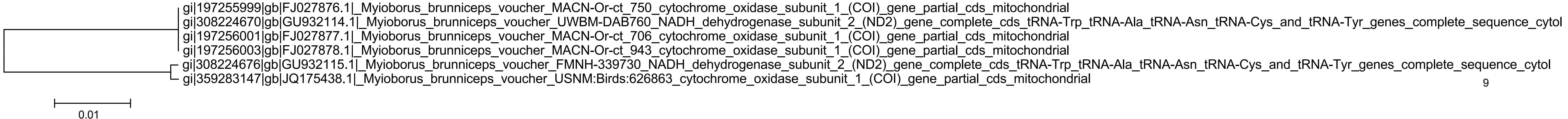
gi|197255999|gb|FJ027876.1|_Myioborus_brunniceps_voucher_MACN-Or-ct_750_cytochrome_oxidase_subunit_1_(COI)_gene_partial_cds_mitochondrial
gi|308224670|gb|GU932114.1|_Myioborus_brunniceps_voucher_UWBM-DAB760_NADH_dehydrogenase_subunit_2_(ND2)_gene_complete_cds_tRNA-Trp_tRNA-Ala_tRNA-Asn_tRNA-Cys_and_tRNA-Tyr_genes_complete_sequence_cytol
gi|197256001|gb|FJ027877.1|_Myioborus_brunniceps_voucher_MACN-Or-ct_706_cytochrome_oxidase_subunit_1_(COI)_gene_partial_cds_mitochondrial
gi|197256003|gb|FJ027878.1|_Myioborus_brunniceps_voucher_MACN-Or-ct_943_cytochrome_oxidase_subunit_1_(COI)_gene_partial_cds_mitochondrial
gi|308224676|gb|GU932115.1|_Myioborus_brunniceps_voucher_FMNH-339730_NADH_dehydrogenase_subunit_2_(ND2)_gene_complete_cds_tRNA-Trp_tRNA-Ala_tRNA-Asn_tRNA-Cys_and_tRNA-Tyr_genes_complete_sequence_cytol
gi|359283147|gb|JQ175438.1|_Myioborus_brunniceps_voucher_USNM:Birds:626863_cytochrome_oxidase_subunit_1_(COI)_gene_partial_cds_mitochondrial
0.01

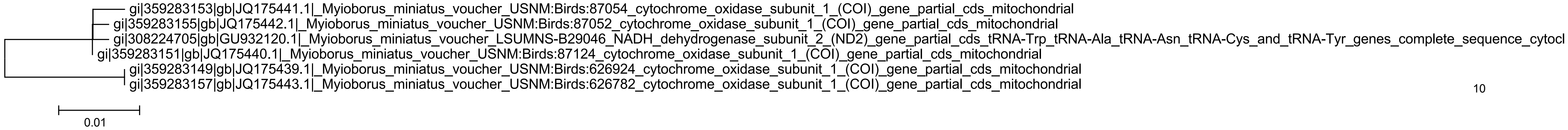
gi|359283153|gb|JQ175441.1|_Myioborus_miniatus_voucher_USNM:Birds:87054_cytochrome_oxidase_subunit_1_(COI)_gene_partial_cds_mitochondrial
gi|359283155|gb|JQ175442.1|_Myioborus_miniatus_voucher_USNM:Birds:87052_cytochrome_oxidase_subunit_1_(COI)_gene_partial_cds_mitochondrial
gi|308224705|gb|GU932120.1|_Myioborus_miniatus_voucher_LSUMNS-B29046_NADH_dehydrogenase_subunit_2_(ND2)_gene_partial_cds_tRNA-Trp_tRNA-Ala_tRNA-Asn_tRNA-Cys_and_tRNA-Tyr_genes_complete_sequence_cytocl
gi|359283151|gb|JQ175440.1|_Myioborus_miniatus_voucher_USNM:Birds:87124_cytochrome_oxidase_subunit_1_(COI)_gene_partial_cds_mitochondrial
gi|359283149|gb|JQ175439.1|_Myioborus_miniatus_voucher_USNM:Birds:626924_cytochrome_oxidase_subunit_1_(COI)_gene_partial_cds_mitochondrial
gi|359283157|gb|JQ175443.1|_Myioborus_miniatus_voucher_USNM:Birds:626782_cytochrome_oxidase_subunit_1_(COI)_gene_partial_cds_mitochondrial
0.01

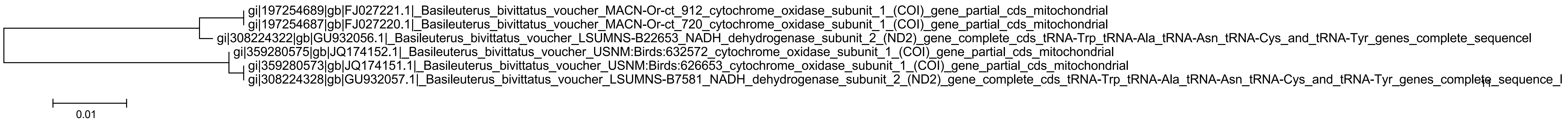
gi|197254689|gb|FJ027221.1|_Basileuterus_bivittatus_voucher_MACN-Or-ct_912_cytochrome_oxidase_subunit_1_(COI)_gene_partial_cds_mitochondrial
gi|197254687|gb|FJ027220.1|_Basileuterus_bivittatus_voucher_MACN-Or-ct_720_cytochrome_oxidase_subunit_1_(COI)_gene_partial_cds_mitochondrial
gi|308224322|gb|GU932056.1|_Basileuterus_bivittatus_voucher_LSUMNS-B22653_NADH_dehydrogenase_subunit_2_(ND2)_gene_complete_cds_tRNA-Trp_tRNA-Ala_tRNA-Asn_tRNA-Cys_and_tRNA-Tyr_genes_complete_sequencel
gi|359280575|gb|JQ174152.1|_Basileuterus_bivittatus_voucher_USNM:Birds:632572_cytochrome_oxidase_subunit_1_(COI)_gene_partial_cds_mitochondrial
gi|359280573|gb|JQ174151.1|_Basileuterus_bivittatus_voucher_USNM:Birds:626653_cytochrome_oxidase_subunit_1_(COI)_gene_partial_cds_mitochondrial
gi|308224328|gb|GU932057.1|_Basileuterus_bivittatus_voucher_LSUMNS-B7581_NADH_dehydrogenase_subunit_2_(ND2)_gene_complete_cds_tRNA-Trp_tRNA-Ala_tRNA-Asn_tRNA-Cys_and_tRNA-Tyr_genes_complete_sequence_l
0.01

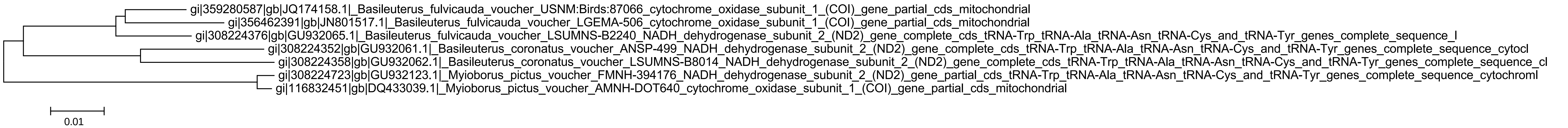
gi|359280587|gb|JQ174158.1|_Basileuterus_fulvicauda_voucher_USNM:Birds:87066_cytochrome_oxidase_subunit_1_(COI)_gene_partial_cds_mitochondrial
gi|356462391|gb|JN801517.1|_Basileuterus_fulvicauda_voucher_LGEMA-506_cytochrome_oxidase_subunit_1_(COI)_gene_partial_cds_mitochondrial
gi|308224376|gb|GU932065.1|_Basileuterus_fulvicauda_voucher_LSUMNS-B2240_NADH_dehydrogenase_subunit_2_(ND2)_gene_complete_cds_tRNA-Trp_tRNA-Ala_tRNA-Asn_tRNA-Cys_and_tRNA-Tyr_genes_complete_sequence_I
gi|308224352|gb|GU932061.1|_Basileuterus_coronatus_voucher_ANSP-499_NADH_dehydrogenase_subunit_2_(ND2)_gene_complete_cds_tRNA-Trp_tRNA-Ala_tRNA-Asn_tRNA-Cys_and_tRNA-Tyr_genes_complete_sequence_cytocl
gi|308224358|gb|GU932062.1|_Basileuterus_coronatus_voucher_LSUMNS-B8014_NADH_dehydrogenase_subunit_2_(ND2)_gene_complete_cds_tRNA-Trp_tRNA-Ala_tRNA-Asn_tRNA-Cys_and_tRNA-Tyr_genes_complete_sequence_cl
gi|308224723|gb|GU932123.1|_Myioborus_pictus_voucher_FMNH-394176_NADH_dehydrogenase_subunit_2_(ND2)_gene_partial_cds_tRNA-Trp_tRNA-Ala_tRNA-Asn_tRNA-Cys_and_tRNA-Tyr_genes_complete_sequence_cytochroml
gi|116832451|gb|DQ433039.1|_Myioborus_pictus_voucher_AMNH-DOT640_cytochrome_oxidase_subunit_1_(COI)_gene_partial_cds_mitochondrial
0.01

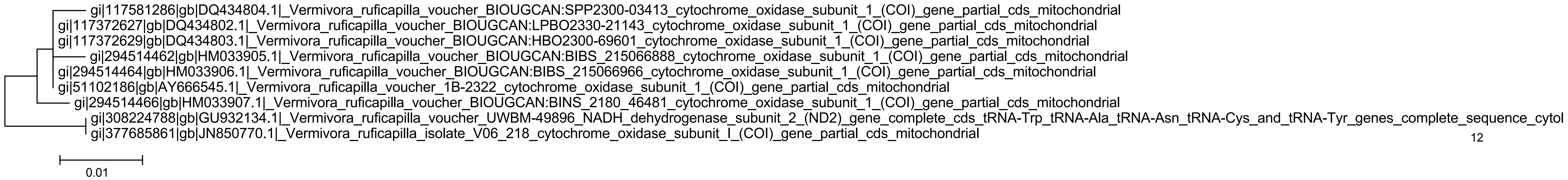
gi|117581286|gb|DQ434804.1|_Vermivora_ruficapilla_voucher_BIOUGCAN:SPP2300-03413_cytochrome_oxidase_subunit_1_(COI)_gene_partial_cds_mitochondrial
gi|117372627|gb|DQ434802.1|_Vermivora_ruficapilla_voucher_BIOUGCAN:LPBO2330-21143_cytochrome_oxidase_subunit_1_(COI)_gene_partial_cds_mitochondrial
gi|117372629|gb|DQ434803.1|_Vermivora_ruficapilla_voucher_BIOUGCAN:HBO2300-69601_cytochrome_oxidase_subunit_1_(COI)_gene_partial_cds_mitochondrial
gi|294514462|gb|HM033905.1|_Vermivora_ruficapilla_voucher_BIOUGCAN:BIBS_215066888_cytochrome_oxidase_subunit_1_(COI)_gene_partial_cds_mitochondrial
gi|294514464|gb|HM033906.1|_Vermivora_ruficapilla_voucher_BIOUGCAN:BIBS_215066966_cytochrome_oxidase_subunit_1_(COI)_gene_partial_cds_mitochondrial
gi|51102186|gb|AY666545.1|_Vermivora_ruficapilla_voucher_1B-2322_cytochrome_oxidase_subunit_1_(COI)_gene_partial_cds_mitochondrial
gi|294514466|gb|HM033907.1|_Vermivora_ruficapilla_voucher_BIOUGCAN:BINS_2180_46481_cytochrome_oxidase_subunit_1_(COI)_gene_partial_cds_mitochondrial
gi|308224788|gb|GU932134.1|_Vermivora_ruficapilla_voucher_UWBM-49896_NADH_dehydrogenase_subunit_2_(ND2)_gene_complete_cds_tRNA-Trp_tRNA-Ala_tRNA-Asn_tRNA-Cys_and_tRNA-Tyr_genes_complete_sequence_cytol
gi|377685861|gb|JN850770.1|_Vermivora_ruficapilla_isolate_V06_218_cytochrome_oxidase_subunit_I_(COI)_gene_partial_cds_mitochondrial
0.01

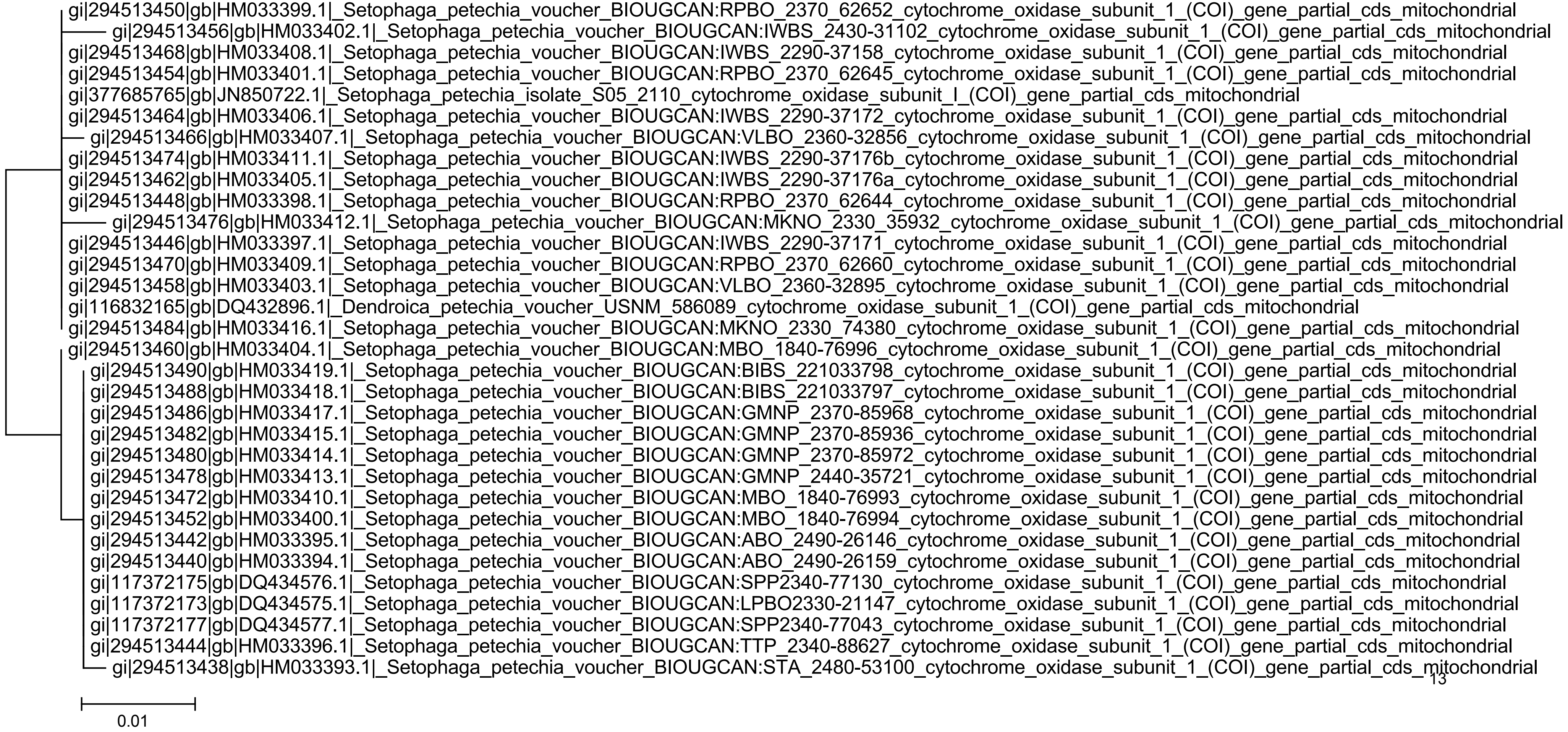
gi|294513450|gb|HM033399.1|_Setophaga_petechia_voucher_BIOUGCAN:RPBO_2370_62652_cytochrome_oxidase_subunit_1_(COI)_gene_partial_cds_mitochondrial
gi|294513456|gb|HM033402.1|_Setophaga_petechia_voucher_BIOUGCAN:IWBS_2430-31102_cytochrome_oxidase_subunit_1_(COI)_gene_partial_cds_mitochondrial
gi|294513468|gb|HM033408.1|_Setophaga_petechia_voucher_BIOUGCAN:IWBS_2290-37158_cytochrome_oxidase_subunit_1_(COI)_gene_partial_cds_mitochondrial
gi|294513454|gb|HM033401.1|_Setophaga_petechia_voucher_BIOUGCAN:RPBO_2370_62645_cytochrome_oxidase_subunit_1_(COI)_gene_partial_cds_mitochondrial
gi|377685765|gb|JN850722.1|_Setophaga_petechia_isolate_S05_2110_cytochrome_oxidase_subunit_I_(COI)_gene_partial_cds_mitochondrial
gi|294513464|gb|HM033406.1|_Setophaga_petechia_voucher_BIOUGCAN:IWBS_2290-37172_cytochrome_oxidase_subunit_1_(COI)_gene_partial_cds_mitochondrial
gi|294513466|gb|HM033407.1|_Setophaga_petechia_voucher_BIOUGCAN:VLBO_2360-32856_cytochrome_oxidase_subunit_1_(COI)_gene_partial_cds_mitochondrial
gi|294513474|gb|HM033411.1|_Setophaga_petechia_voucher_BIOUGCAN:IWBS_2290-37176b_cytochrome_oxidase_subunit_1_(COI)_gene_partial_cds_mitochondrial
gi|294513462|gb|HM033405.1|_Setophaga_petechia_voucher_BIOUGCAN:IWBS_2290-37176a_cytochrome_oxidase_subunit_1_(COI)_gene_partial_cds_mitochondrial
gi|294513448|gb|HM033398.1|_Setophaga_petechia_voucher_BIOUGCAN:RPBO_2370_62644_cytochrome_oxidase_subunit_1_(COI)_gene_partial_cds_mitochondrial
gi|294513476|gb|HM033412.1|_Setophaga_petechia_voucher_BIOUGCAN:MKNO_2330_35932_cytochrome_oxidase_subunit_1_(COI)_gene_partial_cds_mitochondrial
gi|294513446|gb|HM033397.1|_Setophaga_petechia_voucher_BIOUGCAN:IWBS_2290-37171_cytochrome_oxidase_subunit_1_(COI)_gene_partial_cds_mitochondrial
gi|294513470|gb|HM033409.1|_Setophaga_petechia_voucher_BIOUGCAN:RPBO_2370_62660_cytochrome_oxidase_subunit_1_(COI)_gene_partial_cds_mitochondrial
gi|294513458|gb|HM033403.1|_Setophaga_petechia_voucher_BIOUGCAN:VLBO_2360-32895_cytochrome_oxidase_subunit_1_(COI)_gene_partial_cds_mitochondrial
gi|116832165|gb|DQ432896.1|_Dendroica_petechia_voucher_USNM_586089_cytochrome_oxidase_subunit_1_(COI)_gene_partial_cds_mitochondrial
gi|294513484|gb|HM033416.1|_Setophaga_petechia_voucher_BIOUGCAN:MKNO_2330_74380_cytochrome_oxidase_subunit_1_(COI)_gene_partial_cds_mitochondrial
gi|294513460|gb|HM033404.1|_Setophaga_petechia_voucher_BIOUGCAN:MBO_1840-76996_cytochrome_oxidase_subunit_1_(COI)_gene_partial_cds_mitochondrial
gi|294513490|gb|HM033419.1|_Setophaga_petechia_voucher_BIOUGCAN:BIBS_221033798_cytochrome_oxidase_subunit_1_(COI)_gene_partial_cds_mitochondrial
gi|294513488|gb|HM033418.1|_Setophaga_petechia_voucher_BIOUGCAN:BIBS_221033797_cytochrome_oxidase_subunit_1_(COI)_gene_partial_cds_mitochondrial
gi|294513486|gb|HM033417.1|_Setophaga_petechia_voucher_BIOUGCAN:GMNP_2370-85968_cytochrome_oxidase_subunit_1_(COI)_gene_partial_cds_mitochondrial
gi|294513482|gb|HM033415.1|_Setophaga_petechia_voucher_BIOUGCAN:GMNP_2370-85936_cytochrome_oxidase_subunit_1_(COI)_gene_partial_cds_mitochondrial
gi|294513480|gb|HM033414.1|_Setophaga_petechia_voucher_BIOUGCAN:GMNP_2370-85972_cytochrome_oxidase_subunit_1_(COI)_gene_partial_cds_mitochondrial
gi|294513478|gb|HM033413.1|_Setophaga_petechia_voucher_BIOUGCAN:GMNP_2440-35721_cytochrome_oxidase_subunit_1_(COI)_gene_partial_cds_mitochondrial
gi|294513472|gb|HM033410.1|_Setophaga_petechia_voucher_BIOUGCAN:MBO_1840-76993_cytochrome_oxidase_subunit_1_(COI)_gene_partial_cds_mitochondrial
gi|294513452|gb|HM033400.1|_Setophaga_petechia_voucher_BIOUGCAN:MBO_1840-76994_cytochrome_oxidase_subunit_1_(COI)_gene_partial_cds_mitochondrial
gi|294513442|gb|HM033395.1|_Setophaga_petechia_voucher_BIOUGCAN:ABO_2490-26146_cytochrome_oxidase_subunit_1_(COI)_gene_partial_cds_mitochondrial
gi|294513440|gb|HM033394.1|_Setophaga_petechia_voucher_BIOUGCAN:ABO_2490-26159_cytochrome_oxidase_subunit_1_(COI)_gene_partial_cds_mitochondrial
gi|117372175|gb|DQ434576.1|_Setophaga_petechia_voucher_BIOUGCAN:SPP2340-77130_cytochrome_oxidase_subunit_1_(COI)_gene_partial_cds_mitochondrial
gi|117372173|gb|DQ434575.1|_Setophaga_petechia_voucher_BIOUGCAN:LPBO2330-21147_cytochrome_oxidase_subunit_1_(COI)_gene_partial_cds_mitochondrial
gi|117372177|gb|DQ434577.1|_Setophaga_petechia_voucher_BIOUGCAN:SPP2340-77043_cytochrome_oxidase_subunit_1_(COI)_gene_partial_cds_mitochondrial
gi|294513444|gb|HM033396.1|_Setophaga_petechia_voucher_BIOUGCAN:TTP_2340-88627_cytochrome_oxidase_subunit_1_(COI)_gene_partial_cds_mitochondrial
gi|294513438|gb|HM033393.1|_Setophaga_petechia_voucher_BIOUGCAN:STA_2480-53100_cytochrome_oxidase_subunit_1_(COI)_gene_partial_cds_mitochondrial
13
0.01

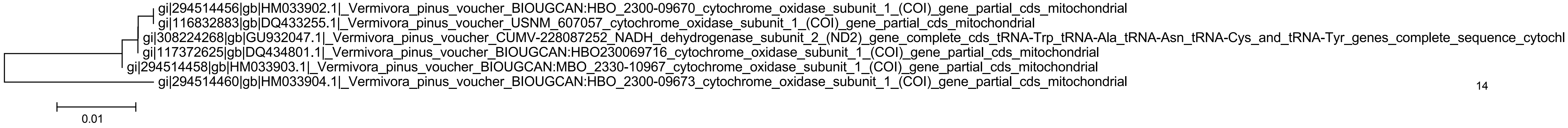

gi|294514456|gb|HM033902.1|_Vermivora_pinus_voucher_BIOUGCAN:HBO_2300-09670_cytochrome_oxidase_subunit_1_(COI)_gene_partial_cds_mitochondrial
gi|116832883|gb|DQ433255.1|_Vermivora_pinus_voucher_USNM_607057_cytochrome_oxidase_subunit_1_(COI)_gene_partial_cds_mitochondrial
gi|308224268|gb|GU932047.1|_Vermivora_pinus_voucher_CUMV-228087252_NADH_dehydrogenase_subunit_2_(ND2)_gene_complete_cds_tRNA-Trp_tRNA-Ala_tRNA-Asn_tRNA-Cys_and_tRNA-Tyr_genes_complete_sequence_cytochl
gi|117372625|gb|DQ434801.1|_Vermivora_pinus_voucher_BIOUGCAN:HBO230069716_cytochrome_oxidase_subunit_1_(COI)_gene_partial_cds_mitochondrial
gi|294514458|gb|HM033903.1|_Vermivora_pinus_voucher_BIOUGCAN:MBO_2330-10967_cytochrome_oxidase_subunit_1_(COI)_gene_partial_cds_mitochondrial
gi|294514460|gb|HM033904.1|_Vermivora_pinus_voucher_BIOUGCAN:HBO_2300-09673_cytochrome_oxidase_subunit_1_(COI)_gene_partial_cds_mitochondrial
0.01

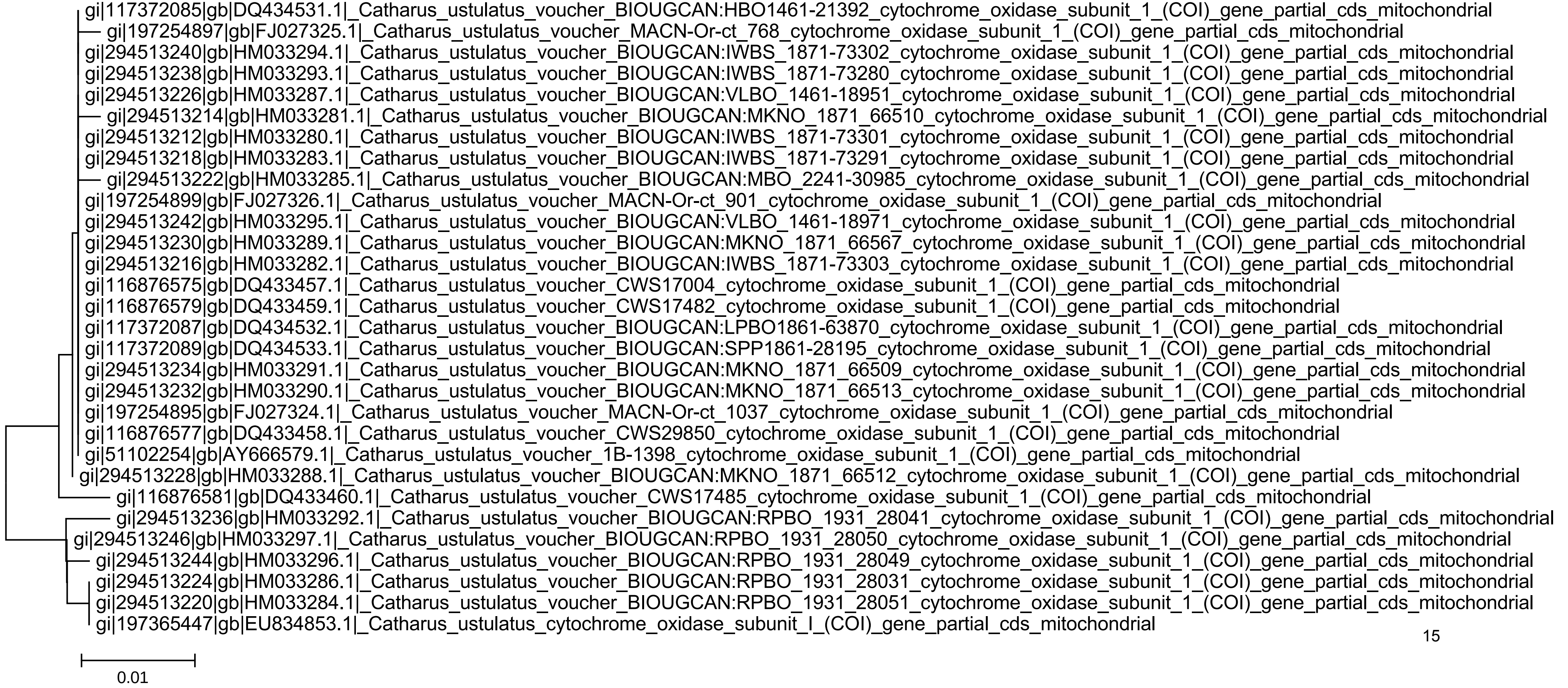
gi|117372085|gb|DQ434531.1|_Catharus_ustulatus_voucher_BIOUGCAN:HBO1461-21392_cytochrome_oxidase_subunit_1_(COI)_gene_partial_cds_mitochondrial
gi|197254897|gb|FJ027325.1|_Catharus_ustulatus_voucher_MACN-Or-ct_768_cytochrome_oxidase_subunit_1_(COI)_gene_partial_cds_mitochondrial
gi|294513240|gb|HM033294.1|_Catharus_ustulatus_voucher_BIOUGCAN:IWBS_1871-73302_cytochrome_oxidase_subunit_1_(COI)_gene_partial_cds_mitochondrial
gi|294513238|gb|HM033293.1|_Catharus_ustulatus_voucher_BIOUGCAN:IWBS_1871-73280_cytochrome_oxidase_subunit_1_(COI)_gene_partial_cds_mitochondrial
gi|294513226|gb|HM033287.1|_Catharus_ustulatus_voucher_BIOUGCAN:VLBO_1461-18951_cytochrome_oxidase_subunit_1_(COI)_gene_partial_cds_mitochondrial
gi|294513214|gb|HM033281.1|_Catharus_ustulatus_voucher_BIOUGCAN:MKNO_1871_66510_cytochrome_oxidase_subunit_1_(COI)_gene_partial_cds_mitochondrial
gi|294513212|gb|HM033280.1|_Catharus_ustulatus_voucher_BIOUGCAN:IWBS_1871-73301_cytochrome_oxidase_subunit_1_(COI)_gene_partial_cds_mitochondrial
gi|294513218|gb|HM033283.1|_Catharus_ustulatus_voucher_BIOUGCAN:IWBS_1871-73291_cytochrome_oxidase_subunit_1_(COI)_gene_partial_cds_mitochondrial
gi|294513222|gb|HM033285.1|_Catharus_ustulatus_voucher_BIOUGCAN:MBO_2241-30985_cytochrome_oxidase_subunit_1_(COI)_gene_partial_cds_mitochondrial
gi|197254899|gb|FJ027326.1|_Catharus_ustulatus_voucher_MACN-Or-ct_901_cytochrome_oxidase_subunit_1_(COI)_gene_partial_cds_mitochondrial
gi|294513242|gb|HM033295.1|_Catharus_ustulatus_voucher_BIOUGCAN:VLBO_1461-18971_cytochrome_oxidase_subunit_1_(COI)_gene_partial_cds_mitochondrial
gi|294513230|gb|HM033289.1|_Catharus_ustulatus_voucher_BIOUGCAN:MKNO_1871_66567_cytochrome_oxidase_subunit_1_(COI)_gene_partial_cds_mitochondrial
gi|294513216|gb|HM033282.1|_Catharus_ustulatus_voucher_BIOUGCAN:IWBS_1871-73303_cytochrome_oxidase_subunit_1_(COI)_gene_partial_cds_mitochondrial
gi|116876575|gb|DQ433457.1|_Catharus_ustulatus_voucher_CWS17004_cytochrome_oxidase_subunit_1_(COI)_gene_partial_cds_mitochondrial
gi|116876579|gb|DQ433459.1|_Catharus_ustulatus_voucher_CWS17482_cytochrome_oxidase_subunit_1_(COI)_gene_partial_cds_mitochondrial
gi|117372087|gb|DQ434532.1|_Catharus_ustulatus_voucher_BIOUGCAN:LPBO1861-63870_cytochrome_oxidase_subunit_1_(COI)_gene_partial_cds_mitochondrial
gi|117372089|gb|DQ434533.1|_Catharus_ustulatus_voucher_BIOUGCAN:SPP1861-28195_cytochrome_oxidase_subunit_1_(COI)_gene_partial_cds_mitochondrial
gi|294513234|gb|HM033291.1|_Catharus_ustulatus_voucher_BIOUGCAN:MKNO_1871_66509_cytochrome_oxidase_subunit_1_(COI)_gene_partial_cds_mitochondrial
gi|294513232|gb|HM033290.1|_Catharus_ustulatus_voucher_BIOUGCAN:MKNO_1871_66513_cytochrome_oxidase_subunit_1_(COI)_gene_partial_cds_mitochondrial
gi|197254895|gb|FJ027324.1|_Catharus_ustulatus_voucher_MACN-Or-ct_1037_cytochrome_oxidase_subunit_1_(COI)_gene_partial_cds_mitochondrial
gi|116876577|gb|DQ433458.1|_Catharus_ustulatus_voucher_CWS29850_cytochrome_oxidase_subunit_1_(COI)_gene_partial_cds_mitochondrial
gi|51102254|gb|AY666579.1|_Catharus_ustulatus_voucher_1B-1398_cytochrome_oxidase_subunit_1_(COI)_gene_partial_cds_mitochondrial
gi|294513228|gb|HM033288.1|_Catharus_ustulatus_voucher_BIOUGCAN:MKNO_1871_66512_cytochrome_oxidase_subunit_1_(COI)_gene_partial_cds_mitochondrial
gi|116876581|gb|DQ433460.1|_Catharus_ustulatus_voucher_CWS17485_cytochrome_oxidase_subunit_1_(COI)_gene_partial_cds_mitochondrial
gi|294513236|gb|HM033292.1|_Catharus_ustulatus_voucher_BIOUGCAN:RPBO_1931_28041_cytochrome_oxidase_subunit_1_(COI)_gene_partial_cds_mitochondrial
gi|294513246|gb|HM033297.1|_Catharus_ustulatus_voucher_BIOUGCAN:RPBO_1931_28050_cytochrome_oxidase_subunit_1_(COI)_gene_partial_cds_mitochondrial
gi|294513244|gb|HM033296.1|_Catharus_ustulatus_voucher_BIOUGCAN:RPBO_1931_28049_cytochrome_oxidase_subunit_1_(COI)_gene_partial_cds_mitochondrial
gi|294513224|gb|HM033286.1|_Catharus_ustulatus_voucher_BIOUGCAN:RPBO_1931_28031_cytochrome_oxidase_subunit_1_(COI)_gene_partial_cds_mitochondrial
gi|294513220|gb|HM033284.1|_Catharus_ustulatus_voucher_BIOUGCAN:RPBO_1931_28051_cytochrome_oxidase_subunit_1_(COI)_gene_partial_cds_mitochondrial
gi|197365447|gb|EU834853.1|_Catharus_ustulatus_cytochrome_oxidase_subunit_I_(COI)_gene_partial_cds_mitochondrial
0.01

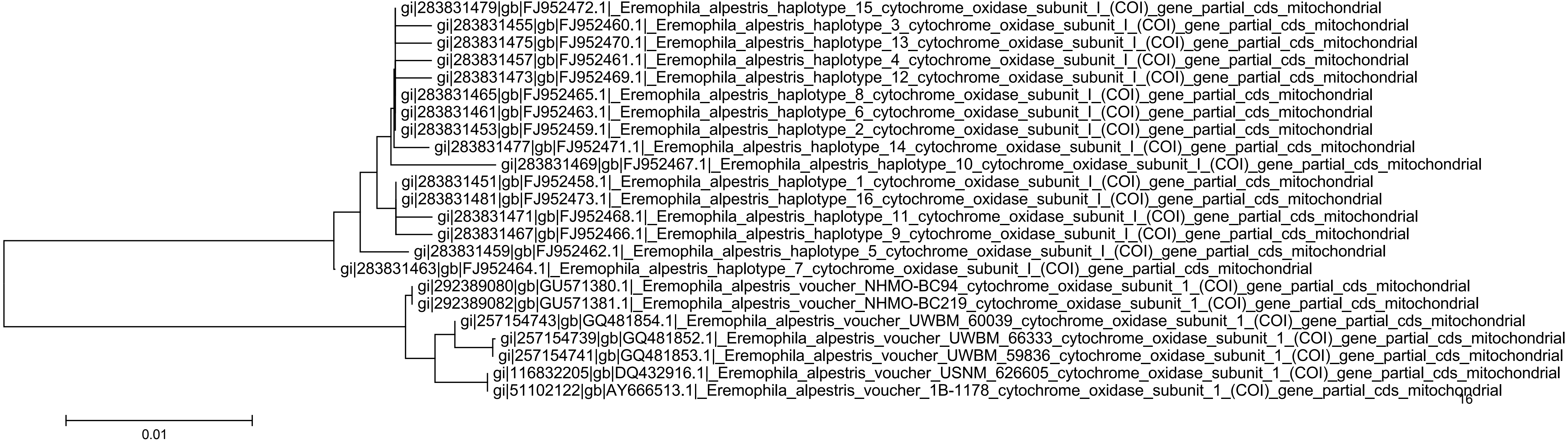
gi|283831479|gb|FJ952472.1|_Eremophila_alpestris_haplotype_15_cytochrome_oxidase_subunit_I_(COI)_gene_partial_cds_mitochondrial
gi|283831455|gb|FJ952460.1|_Eremophila_alpestris_haplotype_3_cytochrome_oxidase_subunit_I_(COI)_gene_partial_cds_mitochondrial
gi|283831475|gb|FJ952470.1|_Eremophila_alpestris_haplotype_13_cytochrome_oxidase_subunit_I_(COI)_gene_partial_cds_mitochondrial
gi|283831457|gb|FJ952461.1|_Eremophila_alpestris_haplotype_4_cytochrome_oxidase_subunit_I_(COI)_gene_partial_cds_mitochondrial
gi|283831473|gb|FJ952469.1|_Eremophila_alpestris_haplotype_12_cytochrome_oxidase_subunit_I_(COI)_gene_partial_cds_mitochondrial
gi|283831465|gb|FJ952465.1|_Eremophila_alpestris_haplotype_8_cytochrome_oxidase_subunit_I_(COI)_gene_partial_cds_mitochondrial
gi|283831461|gb|FJ952463.1|_Eremophila_alpestris_haplotype_6_cytochrome_oxidase_subunit_I_(COI)_gene_partial_cds_mitochondrial
gi|283831453|gb|FJ952459.1|_Eremophila_alpestris_haplotype_2_cytochrome_oxidase_subunit_I_(COI)_gene_partial_cds_mitochondrial
gi|283831477|gb|FJ952471.1|_Eremophila_alpestris_haplotype_14_cytochrome_oxidase_subunit_I_(COI)_gene_partial_cds_mitochondrial
gi|283831469|gb|FJ952467.1|_Eremophila_alpestris_haplotype_10_cytochrome_oxidase_subunit_I_(COI)_gene_partial_cds_mitochondrial
gi|283831451|gb|FJ952458.1|_Eremophila_alpestris_haplotype_1_cytochrome_oxidase_subunit_I_(COI)_gene_partial_cds_mitochondrial
gi|283831481|gb|FJ952473.1|_Eremophila_alpestris_haplotype_16_cytochrome_oxidase_subunit_I_(COI)_gene_partial_cds_mitochondrial
gi|283831471|gb|FJ952468.1|_Eremophila_alpestris_haplotype_11_cytochrome_oxidase_subunit_I_(COI)_gene_partial_cds_mitochondrial
gi|283831467|gb|FJ952466.1|_Eremophila_alpestris_haplotype_9_cytochrome_oxidase_subunit_I_(COI)_gene_partial_cds_mitochondrial
gi|283831459|gb|FJ952462.1|_Eremophila_alpestris_haplotype_5_cytochrome_oxidase_subunit_I_(COI)_gene_partial_cds_mitochondrial
gi|283831463|gb|FJ952464.1|_Eremophila_alpestris_haplotype_7_cytochrome_oxidase_subunit_I_(COI)_gene_partial_cds_mitochondrial
gi|292389080|gb|GU571380.1|_Eremophila_alpestris_voucher_NHMO-BC94_cytochrome_oxidase_subunit_1_(COI)_gene_partial_cds_mitochondrial
gi|292389082|gb|GU571381.1|_Eremophila_alpestris_voucher_NHMO-BC219_cytochrome_oxidase_subunit_1_(COI)_gene_partial_cds_mitochondrial
gi|257154743|gb|GQ481854.1|_Eremophila_alpestris_voucher_UWBM_60039_cytochrome_oxidase_subunit_1_(COI)_gene_partial_cds_mitochondrial
gi|257154739|gb|GQ481852.1|_Eremophila_alpestris_voucher_UWBM_66333_cytochrome_oxidase_subunit_1_(COI)_gene_partial_cds_mitochondrial
gi|257154741|gb|GQ481853.1|_Eremophila_alpestris_voucher_UWBM_59836_cytochrome_oxidase_subunit_1_(COI)_gene_partial_cds_mitochondrial
gi|116832205|gb|DQ432916.1|_Eremophila_alpestris_voucher_USNM_626605_cytochrome_oxidase_subunit_1_(COI)_gene_partial_cds_mitochondrial
gi|51102122|gb|AY666513.1|_Eremophila_alpestris_voucher_1B-1178_cytochrome_oxidase_subunit_1_(COI)_gene_partial_cds_mitochondrial
0.01

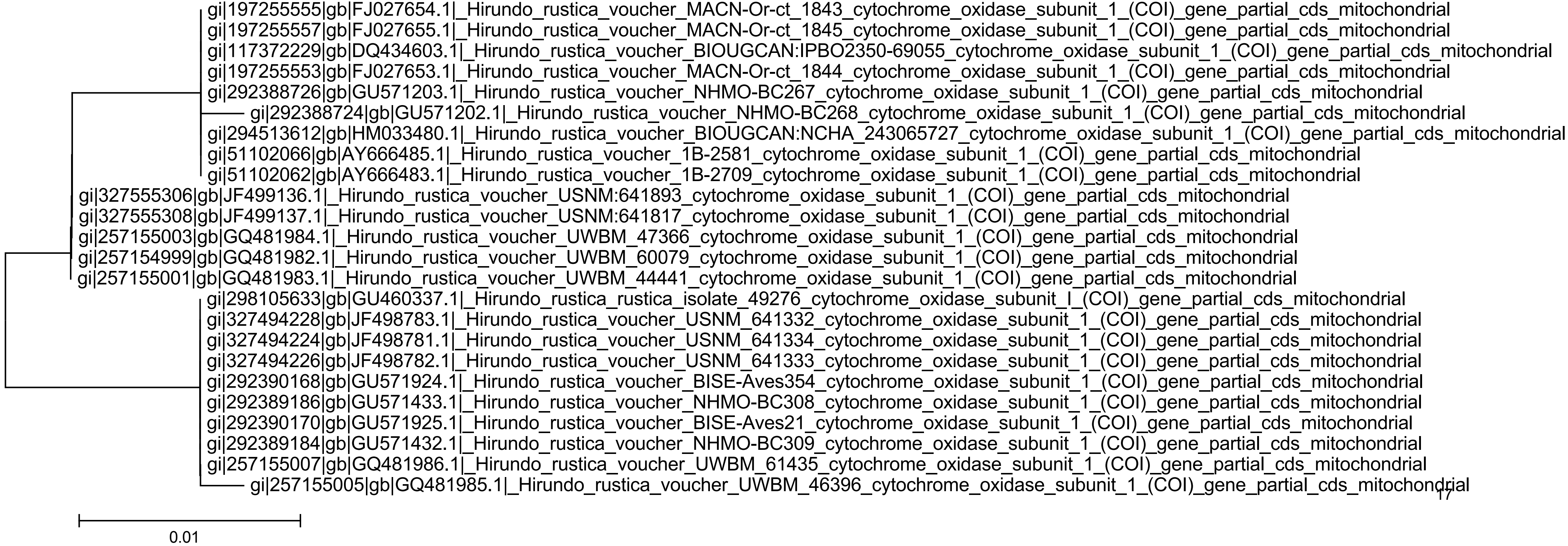
gi|197255555|gb|FJ027654.1|_Hirundo_rustica_voucher_MACN-Or-ct_1843_cytochrome_oxidase_subunit_1_(COI)_gene_partial_cds_mitochondrial
gi|197255557|gb|FJ027655.1|_Hirundo_rustica_voucher_MACN-Or-ct_1845_cytochrome_oxidase_subunit_1_(COI)_gene_partial_cds_mitochondrial
gi|117372229|gb|DQ434603.1|_Hirundo_rustica_voucher_BIOUGCAN:IPBO2350-69055_cytochrome_oxidase_subunit_1_(COI)_gene_partial_cds_mitochondrial
gi|197255553|gb|FJ027653.1|_Hirundo_rustica_voucher_MACN-Or-ct_1844_cytochrome_oxidase_subunit_1_(COI)_gene_partial_cds_mitochondrial
gi|292388726|gb|GU571203.1|_Hirundo_rustica_voucher_NHMO-BC267_cytochrome_oxidase_subunit_1_(COI)_gene_partial_cds_mitochondrial
gi|292388724|gb|GU571202.1|_Hirundo_rustica_voucher_NHMO-BC268_cytochrome_oxidase_subunit_1_(COI)_gene_partial_cds_mitochondrial
gi|294513612|gb|HM033480.1|_Hirundo_rustica_voucher_BIOUGCAN:NCHA_243065727_cytochrome_oxidase_subunit_1_(COI)_gene_partial_cds_mitochondrial
gi|51102066|gb|AY666485.1|_Hirundo_rustica_voucher_1B-2581_cytochrome_oxidase_subunit_1_(COI)_gene_partial_cds_mitochondrial
gi|51102062|gb|AY666483.1|_Hirundo_rustica_voucher_1B-2709_cytochrome_oxidase_subunit_1_(COI)_gene_partial_cds_mitochondrial
gi|327555306|gb|JF499136.1|_Hirundo_rustica_voucher_USNM:641893_cytochrome_oxidase_subunit_1_(COI)_gene_partial_cds_mitochondrial
gi|327555308|gb|JF499137.1|_Hirundo_rustica_voucher_USNM:641817_cytochrome_oxidase_subunit_1_(COI)_gene_partial_cds_mitochondrial
gi|257155003|gb|GQ481984.1|_Hirundo_rustica_voucher_UWBM_47366_cytochrome_oxidase_subunit_1_(COI)_gene_partial_cds_mitochondrial
gi|257154999|gb|GQ481982.1|_Hirundo_rustica_voucher_UWBM_60079_cytochrome_oxidase_subunit_1_(COI)_gene_partial_cds_mitochondrial
gi|257155001|gb|GQ481983.1|_Hirundo_rustica_voucher_UWBM_44441_cytochrome_oxidase_subunit_1_(COI)_gene_partial_cds_mitochondrial
gi|298105633|gb|GU460337.1|_Hirundo_rustica_rustica_isolate_49276_cytochrome_oxidase_subunit_I_(COI)_gene_partial_cds_mitochondrial
gi|327494228|gb|JF498783.1|_Hirundo_rustica_voucher_USNM_641332_cytochrome_oxidase_subunit_1_(COI)_gene_partial_cds_mitochondrial
gi|327494224|gb|JF498781.1|_Hirundo_rustica_voucher_USNM_641334_cytochrome_oxidase_subunit_1_(COI)_gene_partial_cds_mitochondrial
gi|327494226|gb|JF498782.1|_Hirundo_rustica_voucher_USNM_641333_cytochrome_oxidase_subunit_1_(COI)_gene_partial_cds_mitochondrial
gi|292390168|gb|GU571924.1|_Hirundo_rustica_voucher_BISE-Aves354_cytochrome_oxidase_subunit_1_(COI)_gene_partial_cds_mitochondrial
gi|292389186|gb|GU571433.1|_Hirundo_rustica_voucher_NHMO-BC308_cytochrome_oxidase_subunit_1_(COI)_gene_partial_cds_mitochondrial
gi|292390170|gb|GU571925.1|_Hirundo_rustica_voucher_BISE-Aves21_cytochrome_oxidase_subunit_1_(COI)_gene_partial_cds_mitochondrial
gi|292389184|gb|GU571432.1|_Hirundo_rustica_voucher_NHMO-BC309_cytochrome_oxidase_subunit_1_(COI)_gene_partial_cds_mitochondrial
gi|257155007|gb|GQ481986.1|_Hirundo_rustica_voucher_UWBM_61435_cytochrome_oxidase_subunit_1_(COI)_gene_partial_cds_mitochondrial
gi|257155005|gb|GQ481985.1|_Hirundo_rustica_voucher_UWBM_46396_cytochrome_oxidase_subunit_1_(COI)_gene_partial_cds_mitochondrial
0.01

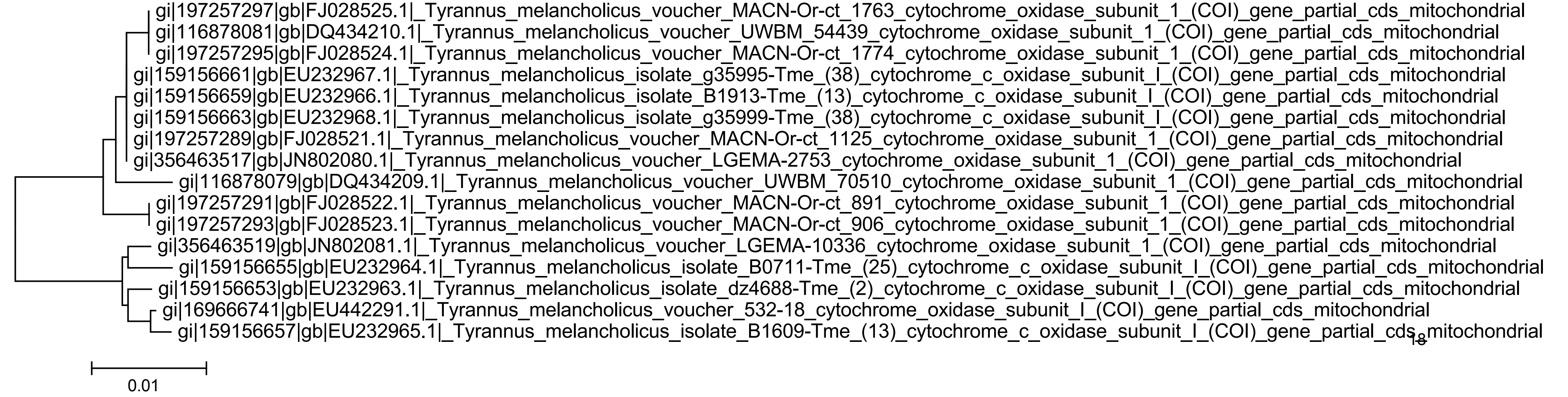
gi|197257297|gb|FJ028525.1|_Tyrannus_melancholicus_voucher_MACN-Or-ct_1763_cytochrome_oxidase_subunit_1_(COI)_gene_partial_cds_mitochondrial
gi|116878081|gb|DQ434210.1|_Tyrannus_melancholicus_voucher_UWBM_54439_cytochrome_oxidase_subunit_1_(COI)_gene_partial_cds_mitochondrial
gi|197257295|gb|FJ028524.1|_Tyrannus_melancholicus_voucher_MACN-Or-ct_1774_cytochrome_oxidase_subunit_1_(COI)_gene_partial_cds_mitochondrial
gi|159156661|gb|EU232967.1|_Tyrannus_melancholicus_isolate_g35995-Tme_(38)_cytochrome_c_oxidase_subunit_I_(COI)_gene_partial_cds_mitochondrial
gi|159156659|gb|EU232966.1|_Tyrannus_melancholicus_isolate_B1913-Tme_(13)_cytochrome_c_oxidase_subunit_I_(COI)_gene_partial_cds_mitochondrial
gi|159156663|gb|EU232968.1|_Tyrannus_melancholicus_isolate_g35999-Tme_(38)_cytochrome_c_oxidase_subunit_I_(COI)_gene_partial_cds_mitochondrial
gi|197257289|gb|FJ028521.1|_Tyrannus_melancholicus_voucher_MACN-Or-ct_1125_cytochrome_oxidase_subunit_1_(COI)_gene_partial_cds_mitochondrial
gi|356463517|gb|JN802080.1|_Tyrannus_melancholicus_voucher_LGEMA-2753_cytochrome_oxidase_subunit_1_(COI)_gene_partial_cds_mitochondrial
gi|116878079|gb|DQ434209.1|_Tyrannus_melancholicus_voucher_UWBM_70510_cytochrome_oxidase_subunit_1_(COI)_gene_partial_cds_mitochondrial
gi|197257291|gb|FJ028522.1|_Tyrannus_melancholicus_voucher_MACN-Or-ct_891_cytochrome_oxidase_subunit_1_(COI)_gene_partial_cds_mitochondrial
gi|197257293|gb|FJ028523.1|_Tyrannus_melancholicus_voucher_MACN-Or-ct_906_cytochrome_oxidase_subunit_1_(COI)_gene_partial_cds_mitochondrial
gi|356463519|gb|JN802081.1|_Tyrannus_melancholicus_voucher_LGEMA-10336_cytochrome_oxidase_subunit_1_(COI)_gene_partial_cds_mitochondrial
gi|159156655|gb|EU232964.1|_Tyrannus_melancholicus_isolate_B0711-Tme_(25)_cytochrome_c_oxidase_subunit_I_(COI)_gene_partial_cds_mitochondrial
gi|159156653|gb|EU232963.1|_Tyrannus_melancholicus_isolate_dz4688-Tme_(2)_cytochrome_c_oxidase_subunit_I_(COI)_gene_partial_cds_mitochondrial
gi|169666741|gb|EU442291.1|_Tyrannus_melancholicus_voucher_532-18_cytochrome_oxidase_subunit_I_(COI)_gene_partial_cds_mitochondrial
gi|159156657|gb|EU232965.1|_Tyrannus_melancholicus_isolate_B1609-Tme_(13)_cytochrome_c_oxidase_subunit_I_(COI)_gene_partial_cds_mitochondrial
0.01

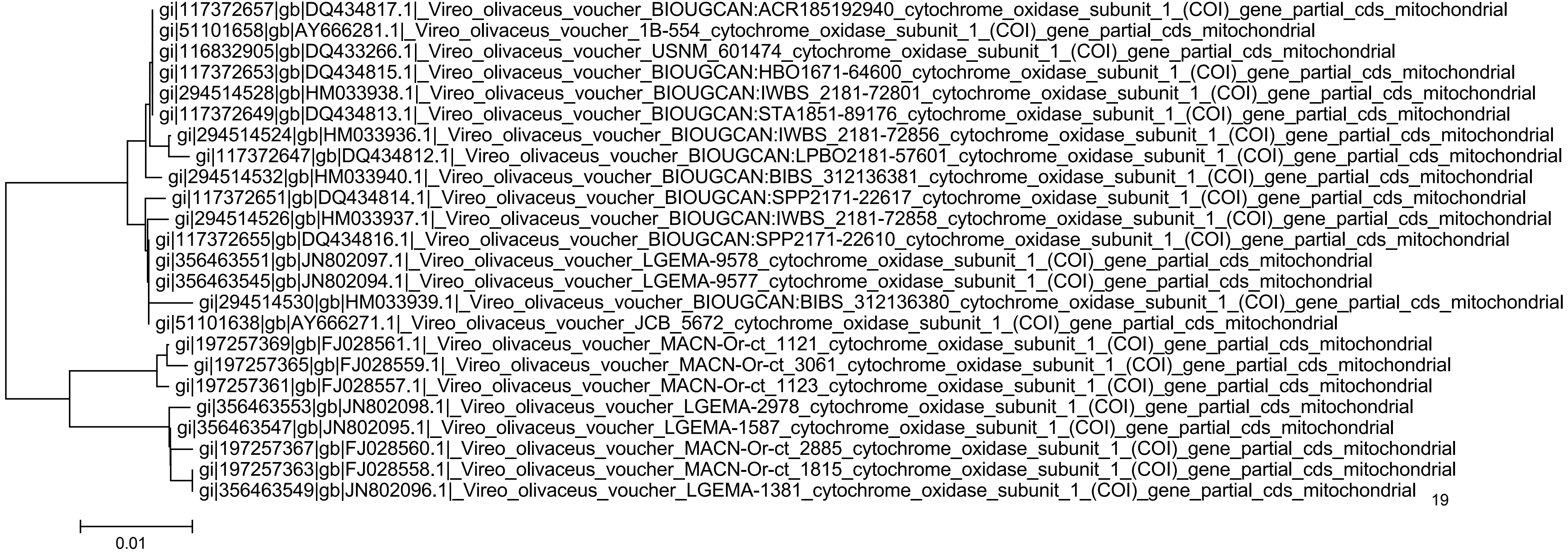
gi|117372657|gb|DQ434817.1|_Vireo_olivaceus_voucher_BIOUGCAN:ACR185192940_cytochrome_oxidase_subunit_1_(COI)_gene_partial_cds_mitochondrial
gi|51101658|gb|AY666281.1|_Vireo_olivaceus_voucher_1B-554_cytochrome_oxidase_subunit_1_(COI)_gene_partial_cds_mitochondrial
gi|116832905|gb|DQ433266.1|_Vireo_olivaceus_voucher_USNM_601474_cytochrome_oxidase_subunit_1_(COI)_gene_partial_cds_mitochondrial
gi|117372653|gb|DQ434815.1|_Vireo_olivaceus_voucher_BIOUGCAN:HBO1671-64600_cytochrome_oxidase_subunit_1_(COI)_gene_partial_cds_mitochondrial
gi|294514528|gb|HM033938.1|_Vireo_olivaceus_voucher_BIOUGCAN:IWBS_2181-72801_cytochrome_oxidase_subunit_1_(COI)_gene_partial_cds_mitochondrial
gi|117372649|gb|DQ434813.1|_Vireo_olivaceus_voucher_BIOUGCAN:STA1851-89176_cytochrome_oxidase_subunit_1_(COI)_gene_partial_cds_mitochondrial
gi|294514524|gb|HM033936.1|_Vireo_olivaceus_voucher_BIOUGCAN:IWBS_2181-72856_cytochrome_oxidase_subunit_1_(COI)_gene_partial_cds_mitochondrial
gi|117372647|gb|DQ434812.1|_Vireo_olivaceus_voucher_BIOUGCAN:LPBO2181-57601_cytochrome_oxidase_subunit_1_(COI)_gene_partial_cds_mitochondrial
gi|294514532|gb|HM033940.1|_Vireo_olivaceus_voucher_BIOUGCAN:BIBS_312136381_cytochrome_oxidase_subunit_1_(COI)_gene_partial_cds_mitochondrial
gi|117372651|gb|DQ434814.1|_Vireo_olivaceus_voucher_BIOUGCAN:SPP2171-22617_cytochrome_oxidase_subunit_1_(COI)_gene_partial_cds_mitochondrial
gi|294514526|gb|HM033937.1|_Vireo_olivaceus_voucher_BIOUGCAN:IWBS_2181-72858_cytochrome_oxidase_subunit_1_(COI)_gene_partial_cds_mitochondrial
gi|117372655|gb|DQ434816.1|_Vireo_olivaceus_voucher_BIOUGCAN:SPP2171-22610_cytochrome_oxidase_subunit_1_(COI)_gene_partial_cds_mitochondrial
gi|356463551|gb|JN802097.1|_Vireo_olivaceus_voucher_LGEMA-9578_cytochrome_oxidase_subunit_1_(COI)_gene_partial_cds_mitochondrial
gi|356463545|gb|JN802094.1|_Vireo_olivaceus_voucher_LGEMA-9577_cytochrome_oxidase_subunit_1_(COI)_gene_partial_cds_mitochondrial
gi|294514530|gb|HM033939.1|_Vireo_olivaceus_voucher_BIOUGCAN:BIBS_312136380_cytochrome_oxidase_subunit_1_(COI)_gene_partial_cds_mitochondrial
gi|51101638|gb|AY666271.1|_Vireo_olivaceus_voucher_JCB_5672_cytochrome_oxidase_subunit_1_(COI)_gene_partial_cds_mitochondrial
gi|197257369|gb|FJ028561.1|_Vireo_olivaceus_voucher_MACN-Or-ct_1121_cytochrome_oxidase_subunit_1_(COI)_gene_partial_cds_mitochondrial
gi|197257365|gb|FJ028559.1|_Vireo_olivaceus_voucher_MACN-Or-ct_3061_cytochrome_oxidase_subunit_1_(COI)_gene_partial_cds_mitochondrial
gi|197257361|gb|FJ028557.1|_Vireo_olivaceus_voucher_MACN-Or-ct_1123_cytochrome_oxidase_subunit_1_(COI)_gene_partial_cds_mitochondrial
gi|356463553|gb|JN802098.1|_Vireo_olivaceus_voucher_LGEMA-2978_cytochrome_oxidase_subunit_1_(COI)_gene_partial_cds_mitochondrial
gi|356463547|gb|JN802095.1|_Vireo_olivaceus_voucher_LGEMA-1587_cytochrome_oxidase_subunit_1_(COI)_gene_partial_cds_mitochondrial
gi|197257367|gb|FJ028560.1|_Vireo_olivaceus_voucher_MACN-Or-ct_2885_cytochrome_oxidase_subunit_1_(COI)_gene_partial_cds_mitochondrial
gi|197257363|gb|FJ028558.1|_Vireo_olivaceus_voucher_MACN-Or-ct_1815_cytochrome_oxidase_subunit_1_(COI)_gene_partial_cds_mitochondrial
gi|356463549|gb|JN802096.1|_Vireo_olivaceus_voucher_LGEMA-1381_cytochrome_oxidase_subunit_1_(COI)_gene_partial_cds_mitochondrial
0.01
