# Supplementary material for: DNA Barcoding Works in Practice but Not in (Neutral) Theory
Source: PLoS One. 2014 Jul 2;9(7):e100755. doi: 10.1371/journal.pone.0100755 (PMC4079456; doi:10.1371/journal.pone.0100755)

Fig. S6. Intraspecific cytochrome b variation unrelated to sample size. Data from Nabholz 2009 dataset (185 species).

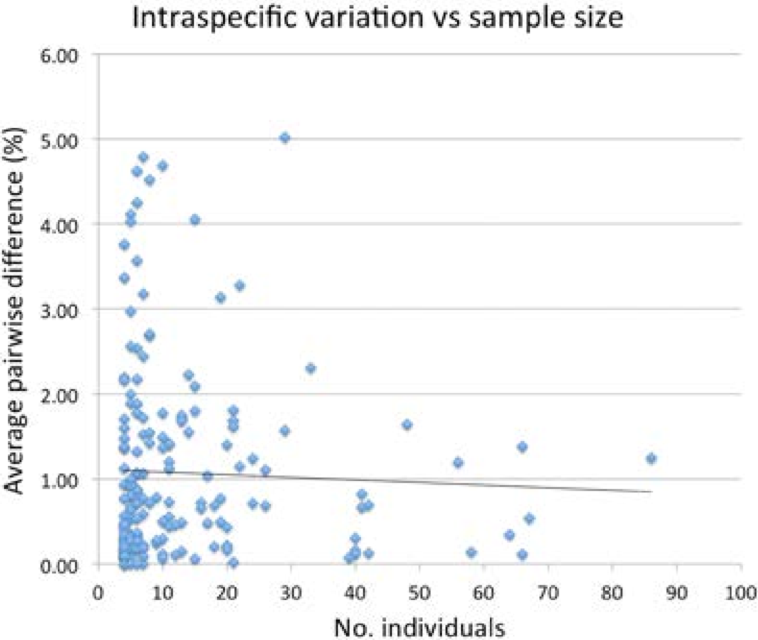

Supplement: Figure S6 — Intraspecific cytochrome b variation is unrelated to sample size (adapted from [30] ). (PDF) [file pone.0100755.s006.pdf]
