# Supplementary material for: DNA Barcoding Works in Practice but Not in (Neutral) Theory
Source: PLoS One. 2014 Jul 2;9(7):e100755. doi: 10.1371/journal.pone.0100755 (PMC4079456; doi:10.1371/journal.pone.0100755)
Supplement: Table S1 — Intraspecific mitochondrial DNA variation in birds is generally low. (PDF) [file pone.0100755.s007.pdf]

Table S1. Intraspecific mtDNA variation in birds is generally low. Number of individuals, average and maximum pairwise distances (%K2P), and world census population estimates are shown. Outlier species, defined as having ≥0.5% average or ≥1.5% maximum pairwise difference, are highlighted in green (n=24) and number of geographic or hybrid clusters are shown. Two species, Cape May Warbler (*Setophaga tigrina*), (average/maximum pairwise difference, 0.44%/1.60%) and Common Starling (*Sturnus vulgaris*), (average/maximum pairwise difference, 0.63%/2.36%) have high variation without evident geographic or hybrid clusters.

Common name	Latin name	No. indivs	ave K2P 519	max K2P 519	No. clusters	World pop
**Scolopacidae (Sandpipers and allies)**						
Common Sandpiper	*Actitis hypoleucos*	17	0.14	0.97		2,750,000
Spotted Sandpiper	*Actitis macularia*	11	0.04	0.19		150,000
Surfbird	*Aphriza virgata*	2	0.00	0.00		70,000
Ruddy Turnstone	*Arenaria interpres*	12	0.17	0.39		510,000
Upland Sandpiper	*Bartramia longicauda*	3	0.13	0.19		500,000
Sanderling	*Calidris alba*	9	0.28	0.78		660,000
Dunlin	*Calidris alpina*	9	0.86	1.37	3	5,500,000
Baird's Sandpiper	*Calidris bairdii*	6	0.00	0.00		300,000
Red Knot	*Calidris canutus*	9	0.00	0.00		1,100,000
Curlew Sandpiper	*Calidris ferruginea*	4	0.29	0.58		1,850,000
White-rumped Sandpiper	*Calidris fuscicollis*	17	0.12	0.48		1,120,000
Purple Sandpiper	*Calidris maritima*	3	0.00	0.00		200,000
Western Sandpiper	*Calidris mauri*	17	0.09	0.40		3,500,000
Pectoral Sandpiper	*Calidris melanotos*	17	0.11	0.45		62,500
Little Stint	*Calidris minuta*	5	0.08	0.19		1,450,000
Least Sandpiper	*Calidris minutilla*	16	0.27	0.78		700,000
Rock Sandpiper	*Calidris ptilocnemis*	2	0.00	0.00		145,000
Semipalmated Sandpiper	*Calidris pusilla*	3	0.00	0.00		2,260,000
Temminck's Stint	*Calidris temminckii*	3	0.00	0.00		735,000
Great Knot	*Calidris tenuirostris*	3	0.00	0.00		380,000
Wilson's Snipe	*Gallinago delicata*	10	0.08	0.39		2,000,000
Common Snipe	*Gallinago gallinago*	21	0.08	0.39		3,500,000
Great Snipe	*Gallinago media*	4	0.10	0.19		585,000
Swinoe's Snipe	*Gallinago megala*	3	0.00	0.00		62,500
South American Snipe	*Gallinago paraguaiae*	5	0.08	0.19		1,025,000
Pin-tailed Snipe	*Gallinago stenura*	6	0.06	0.19		512,500
Giant Snipe	*Gallinago undulata*	3	0.00	0.00		
Broad-billed Sandpiper	*Limicola falcinellus*	4	0.00	0.00		87,000
Short-billed Dowitcher	*Limnodromus griseus*	6	0.13	0.39		245,000
Long-billed Dowitcher	*Limnodromus scolopaceus*	6	0.00	0.00		500,000
Marbled Godwit	*Limosa fedoa*	3	0.00	0.00		171,500
Hudsonian Godwit	*Limosa haemastica*	3	0.13	0.19		77,000
Bar-tailed Godwit	*Limosa lapponica*	7	0.06	0.19		1,124,000
Black-tailed Godwit	*Limosa limosa*	60	0.34	2.16	2	539,000
Jack Snipe	*Lymnocryptes minimus*	6	0.12	0.19		1,000,000
Stilt Sandpiper	*Micropalama himantopus*	3	0.13	0.19		820,000
Long-billed Curlew	*Numenius americanus*	2	0.19	0.19		161,000
Eurasian Curlew	*Numenius arquata*	6	0.10	0.19		917,500
Far Eastern Curlew	*Numenius madagascariensis*	5	0.19	0.39		32,000
Whimbrel	*Numenius phaeopus*	14	1.91	4.01	2	1,575,000
Bristle-thighed Curlew	*Numenius tahitiensis*	6	0.06	0.19		10,000
Red Phalarope	*Phalaropus fulicaria*	8	0.00	0.00		200,000
Red-necked Phalarope	*Phalaropus lobatus*	33	0.05	0.39		3,600,000
Wilson's Phalarope	*Phalaropus tricolor*	2	0.19	0.19		1,500,000
Ruff	*Philomachus pugnax*	23	0.11	0.39		2,300,000
American Woodcock	*Scolopax minor*	5	0.00	0.00		3,500,000
Eurasian Woodcock	*Scolopax rusticola*	11	0.04	0.19		18,018,750
Grey-tailed Tattler	*Tringa brevipes*	4	0.00	0.00		44,000
Spotted Redshank	*Tringa erythropus*	8	0.28	0.59		145,000
Lesser Yellowlegs	*Tringa flavipes*	12	0.14	0.39		400,000
Wood Sandpiper	*Tringa glareola*	17	0.05	0.39		3,300,000
Wandering Tattler	*Tringa incana*	3	0.00	0.00		18,500
Greater Yellowlegs	*Tringa melanoleuca*	9	0.00	0.00		100,000

Common Greenshank	*Tringa nebularia*	11	0.35	0.78		780,000
Green Sandpiper	*Tringa ochropus*	9	0.44	1.37		2,250,000
Willet	*Tringa semipalmatus*	8	0.35	0.93		250,000
Solitary Sandpiper	*Tringa solitaria*	13	2.33	5.25	2	150,000
Marsh Sandpiper	*Tringa stagnatilis*	3	0.13	0.19		730,000
Common Redshank	*Tringa totanus*	17	0.50	1.17	2	1,730,000
Buff-breasted Sandpiper	*Tryngites subruficollis*	2	0.19	0.19		56,500
Terek Sandpiper	*Xenus cinereus*	5	0.00	0.00		550,000
**Parulidae (New World warblers)**						
Golden-crowned Warbler	*Basileuterus culicivorus*	8	0.67	1.76	2	
Black-cheeked Warbler	*Basileuterus melanogenys*	4	0.19	0.39		
Three-striped Warbler	*Basileuterus tristriatus*	4	0.19	0.39		
Canada Warbler	*Cardellina canadensis*	9	0.20	0.58		4,000,000
Wilson's Warbler	*Cardellina pusilla*	32	0.59	1.56	2	60,000,000
Red-faced Warbler	*Cardellina rubifrons*	2	0.00	0.00		700,000
Masked Yellowthroat	*Geothlypis aequinoctialis*	6	0.95	2.56	2	
Kentucky Warbler	*Geothlypis formosus*	8	0.09	0.39		2,800,000
Mourning Warbler	*Geothlypis philadelphia*	8	0.44	0.98		17,000,000
Olive-crowned Yellowthroat	*Geothlypis semiflava*	4	0.00	0.00		
MacGillivray's Warbler	*Geothlypis tolmiei*	11	0.10	0.58		12,000,000
Common Yellowthroat	*Geothlypis trichas*	24	0.46	1.17		87,000,000
Worm-eating Warbler	*Helmitheros vermivorum*	4	0.23	0.39		830,000
Swainson's Warbler	*Limnothlypis swainsonii*	2	0.00	0.00		90,000
Black-and-white Warbler	*Mniotilta varia*	16	0.05	0.39		20,000,000
Brown-capped Whitestart	*Myioborus brunniceps*	6	2.46	4.60	2	
Slate-throated Whitestart	*Myioborus miniatus*	6	1.63	2.96	2	
Painted Redstart	*Myioborus pictus*	2	0.58	0.58		2,000,000
Collared Whitestart	*Myioborus torquatus*	4	0.00	0.00		
Two-banded Warbler	*Myiothlypis bivittata*	6	3.91	6.51	2	
Russet-crowned Warbler	*Myiothlypis coronata*	2	4.80	4.80		
Flavescent Warbler	*Myiothlypis flaveola*	9	0.12	0.39		
Buff-rumped Warbler	*Myiothlypis fulvicauda*	3	3.04	3.58	3	
White-browed Warbler	*Myiothlypis leucoblephara*	5	0.07	0.19		
Riverbank Warbler	*Myiothlypis rivularis*	7	0.00	0.00		
Pale-legged Warbler	*Myiothlypis signata*	3	0.39	0.58		
Connecticut Warbler	*Oporornis agilis*	4	0.20	0.39		1,700,000
Orange-crowned Warbler	*Oreothlypis celata*	23	0.27	0.79		80,000,000
Colima Warbler	*Oreothlypis crissalis*	2	0.00	0.00		30,000
Lucy's Warbler	*Oreothlypis luciae*	3	0.00	0.00		3,000,000
Tennesse Warbler	*Oreothlypis peregrina*	16	0.24	0.97		70,000,000
Nashville Warbler	*Oreothlypis ruficapilla*	9	0.89	1.97	2	32,000,000
Virginia's Warbler	*Oreothlypis virginiae*	4	0.00	0.00		1,100,000
Louisiana Waterthrush	*Parkesia motacilla*	3	0.00	0.00		360,000
Northern Waterthrush	*Parkesia noveboracensis*	25	0.21	0.58		19,000,000
Prothonotary Warbler	*Protonotaria citrea*	6	0.06	0.19		1,600,000
Ovenbird	*Seiurus aurocapilla*	21	0.42	1.17		22,000,000
Northern Parula	*Setophaga americana*	13	0.03	0.19		13,000,000
Black-throated Blue Warbler	*Setophaga caerulescens*	12	0.03	0.19		2,100,000
Bay-breasted Warbler	*Setophaga castanea*	7	0.20	0.39		9,000,000
Cerulean Warbler	*Setophaga cerulea*	3	0.26	0.39		600,000
Hooded Warbler	*Setophaga citrinia*	5	0.00	0.00		4,600,000
Yellow-rumped Warbler	*Setophaga coronata*	32	0.28	0.99		130,000,000
Prairie Warbler	*Setophaga discolor*	5	0.31	0.78		3,500,000
Yellow-throated Warbler	*Setophaga dominica*	4	0.48	0.97		1,800,000
Blackburnian Warbler	*Setophaga fusca*	7	0.06	0.19		10,000,000
Grace's Warbler	*Setophaga graciae*	3	0.13	0.19		2,000,000
Kirtland's Warbler	*Setophaga kirtlandii*	3	0.00	0.00		4,000
Magnolia Warbler	*Setophaga magnolia*	26	0.15	0.58		40,000,000
Black-throated Gray Warbler	*Setophaga nigrescens*	7	0.17	0.58		2,400,000
Hermit Warbler	*Setophaga occidentalis*	5	0.31	0.78		2,500,000
Palm Warbler	*Setophaga palmarum*	13	0.12	0.39		13,000,000
Chestnut-sided Warbler	*Setophaga pennsylvanica*	14	0.25	0.78		19,000,000
Yellow Warbler	*Setophaga petechia*	32	0.68	1.76	2	90,000,000
Pine Warbler	*Setophaga pinus*	4	0.19	0.39		13,000,000
Tropical Parula	*Setophaga pitayumi*	8	0.31	0.99		20,000,000
American Redstart	*Setophaga ruticilla*	22	0.17	0.58		39,000,000
Blackpoll Warbler	*Setophaga striata*	22	0.05	0.39		60,000,000
Cape May Warbler	*Setophaga tigrina*	8	0.44	1.60	1	7,000,000
Townsend's Warbler	*Setophaga townsendi*	10	0.37	0.78		17,000,000
Black-throated Green Warbler	*Setophaga virens*	11	0.26	0.97		10,000,000
Golden-winged Warbler	*Vermivora chrysoptera*	5	0.00	0.00		410,000
Blue-winged Warbler	*Vermivora cyanoptera*	6	1.34	3.78	2	810,000

**Highly abundant > 100M**						
Common Redpoll	*Acanthis flammea*	18	0.15	0.44		160,000,000
Red-winged blackbird	*Agelaius phoeniceus*	14	0.41	0.85		130,000,000
Lapland Longspur	*Calcarius lapponicus*	13	0.47	1.20		130,000,000
Northern Cardinal	*Cardinalis cardinalis*	11	0.21	0.58		120,000,000
Swainson's Thrush	*Catharus ustulatus*	30	0.54	1.84	2	100,000,000
Rock dove	*Columba livia*	29	0.09	0.58		120,000,000
Horned Lark	*Eremophila alpestris*	23	2.29	5.03	4	120,000,000
Barn Swallow	*Hirundo rustica*	24	0.92	2.17	3	120,000,000
Dark eyed Junco	*Junco hyemalis*	60	0.05	0.59		200,000,000
Shiny Cowbird	*Molothrus bonariensis*	10	0.00	0.00		200,000,000
House sparrow	*Passer domesticus*	39	0.11	0.39		540,000,000
Yellow-rumped warbler	*Setophaga coronata*	32	0.28	0.99		130,000,000
Chipping Sparrow	*Spizella passerina*	21	0.07	0.39		230,000,000
Common Starling	*Sturnus vulgaris*	23	0.63	2.36	1	150,000,000
American robin	*Turdus migratorius*	29	0.19	0.58		310,000,000
Tropical Kingbird	*Tyrannus melancholicus*	16	1.30	2.56	2	200,000,000
Red-eyed vireo	*Vireo olivaceous*	24	1.67	3.38	3	180,000,000
Mourning Dove	*Zenaida macroura*	14	0.03	0.20		120,000,000
White-throated sparrow	*Zonotrichia albicollis*	27	0.13	0.39		140,000,000
