# Supplementary material for: DNA Barcoding Works in Practice but Not in (Neutral) Theory
Source: PLoS One. 2014 Jul 2;9(7):e100755. doi: 10.1371/journal.pone.0100755 (PMC4079456; doi:10.1371/journal.pone.0100755)
Supplement: Table S2 — Variation within avian geographic and hybrid clusters is low. (PDF) [file pone.0100755.s008.pdf]

Table S2. Variation within avian geographic, hybrid clusters is low. Outlier species highlighted in Table S1 are shown with clusters analyzed separately. For each cluster, number of individuals, average and maximum variation (values ≥0.5% average or ≥1.5% maximum highlighted in green), geographic range or hybridizing species, and supporting references are listed. Maps of collection locations and corresponding clusters are available in Supporting Information Figs. S2-S5.

		No. indivs	ave K2P519	maxK2P519	Range	Supporting references
**Scolopacidae (Sandpipers and allies)**						
Dunlin	*Calidris alpina*	4	0.0000	0.0000	Europe	1
	Calidris alpina	3	0.3868	0.0000	e Palearctic, Alaska	
	Calidris alpina	2	0.0000	0.3868	N America	
Black-tailed Godwit	*Limosa limosa*	55	0.0343	0.3868	w Palearctic	2
	Limosa limosa	5	0.0000	0.0000	e Palearctic	
Whimbrel	*Numenius phaeopus*	9	0.2832	0.5814	Europe	3
	Numenius phaeopus	5	0.0783	0.2041	N America	
Solitary Sandpiper	*Tringa solitaria*	9	0.1396	0.3884	w N America, Argentina	4
	Tringa solitaria	4	0.0000	0.0000	e N America	
Common Redshank	*Tringa totanus*	11	0.0351	0.1931	w Palearctic	
	Tringa totanus	6	0.0000	0.0000	e Palearctic	
**Parulidae (New World warblers)**						
Golden-crowned Warbler	*Basileuterus culicivorus*	6	0.1934	0.5814	Argentina, Brazil	5
	Basileuterus culicivorus	1	-	-	Bolivia	
	Basileuterus culicivorus	1			Trinidad	
Wilson's Warbler	*Cardellina pusilla*	22	0.2840	0.9712	w N America	6
	Cardellina pusilla	10	0.2508	0.9988	e N America	
Masked Yellowthroat	*Geothlypis aequinoctialis*	5	0.2319	0.5806	e Argentina, Uruguay	7
	Geothlypis aequinoctialis	1	-	-	w Argentina	
Brown-capped Whitestart	*Myioborus brunniceps*	4	0.0000	0.0000	Argentina	8
	Myioborus brunniceps	2	0.1930	0.1930	Guyana, Venezuela	
Slate-throated Whitestart	*Myioborus miniatus*	4	0.3871	0.5814	Panama	8
	Myioborus miniatus	2	0.0000	0.0000	Guyana	
Painted Redstart	*Myioborus pictus*	1	-	-	Arizona	
	Myioborus pictus	1	-	-	s Mexico	
Two-banded Warbler	*Myiothlypis bivittata*	3	0.5170	0.7755	Argentina, Bolivia	9
	Myiothlypis bivittata	3	0.1286	0.1930	Guyana, Venezuela	
Russet-crowned Warbler	*Myiothlypis coronata*	1	-	-	Peru	9
	Myiothlypis coronata	1	-	-	Ecuador	
Buff-rumped Warbler	*Myiothlypis fulvicauda*	1	-	-	n Panama	10
	Myiothlypis fulvicauda	1	-	-	s Panama	
	Myiothlypis fulvicauda	1	-	-	Brazil	
Nashville Warbler	*Oreothlypis ruficapilla*	7	0.3893	0.9807	e N America	11
	Oreothlypis ruficapilla	2	0.0000	0.0000	w N America	
Yellow Warbler	*Setophaga petechia*	16	0.0483	0.3868	e N America	12
	Setophaga petechia	16	0.1208	0.7755	w N America	
Cape May Warbler	*Setophaga tigrina*	8	0.4432	1.5969	N Amer	
Blue-winged Warbler	*Vermivora cyanoptera*	5	0.1932	0.3868	*V. cyanoptera* group	13
	Vermivora cyanoptera	1	-	-	*V. chrysoptera* group	
**Highly abundant > 100M**						
Swainson's Thrush	*Catharus ustulatus*	24	0.0992	0.8126	inland N America	14,15
	Catharus ustulatus	6	0.3097	0.5814	Pacific coast N America	
Horned Lark	*Eremophila alpestris*	16	0.4002	0.9728	China	16
	Eremophila alpestris	3	0.1290	0.1941	Russia	
	Eremophila alpestris	2	0.0000	0.0000	Norway	
	Eremophila alpestris	2	0.0000	0.0000	US	
Barn Swallow	*Hirundo rustica*	10	0.0386	0.1931	Europe	17
	Hirundo rustica	9	0.0430	0.1946	N America	
	Hirundo rustica	5	0.0000	0.0000	Japan	
Common Starling	*Sturnus vulgaris*	23	0.6259	2.3638	World	
Tropical Kingbird	*Tyrannus melancholicus*	11	0.3808	1.1696	Argentina, Brazil	
	Tyrannus melancholicus	5	0.5654	0.7767	Brazil, Guatemala	
Red-eyed vireo	*Vireo olivaceous*	16	0.3910	1.1696	N Amer	18
	Vireo olivaceous	5	0.2706	0.3868	Brazil, Argentina	
	Vireo olivaceous	3	0.2575	0.3864	Argentina	
