# Supplementary material for: DNA Barcoding Works in Practice but Not in (Neutral) Theory
Source: PLoS One. 2014 Jul 2;9(7):e100755. doi: 10.1371/journal.pone.0100755 (PMC4079456; doi:10.1371/journal.pone.0100755)
Supplement: Table S3 — Re-analysis of intraspecific variation in Nabholz 2009 [30] avian cytochrome b dataset. (PDF) [file pone.0100755.s009.pdf]

Table S3. Re-analysis of intraspecific variation in Nabholz 2009 [30] avian cytochrome b dataset. Taxonomic classification and number of individuals for all species with ≥0.5% average pairwise difference are shown (n=109 of 185 in series). Intraspecific variation was unrelated to sample size in this dataset (Fig. S6). Species with geographic (G) (n=85) or hybrid (H) (n=1) clusters, lacking clusters (N) (n=5), or with insufficient data (I) (n=17) are indicated and supporting references are listed. Most species with geographic clusters are officially split or recommended to be split into two or more species (S) (n=47). In addition, *Passer montanus* dataset includes two probable pseudogene sequences based on similarity to other *Passer* pseudogenes (P). Abbreviations: American Ornithologists' Union (AOU), mitochondrial control region (CR), International Ornithologists' Union (IOC), South American Classification Committee (SACC).

**Order**	**Family**	**Species**	**No. seqs**	**Sequence length**	**Ave pairwise difference**	**Clusters**	**Comments**	**References**
Ratites	Apterygidae	*Apteryx australis*	21	654	1.81	G,S	differ among islands in New Zealand	19
Galleoansereae	Anatidae	*Branta canadensis*	5	306	0.98	G,S	clusters match subspecies; split AOU	20
Galleoansereae	Anseridae	*Anser cygnoides*	19	1143	0.77	I	domesticated strains	
Galleoansereae	Phasianidae	*Tetraogallus himalayensis*	16	533	0.66	G	eastern, western Himalayan plateau China	21
Neoaves	Accipitridae	*Pernis celebensis*	10	330	1.78	G,S	clusters match subspecies	22
Neoaves	Accipitridae	*Pernis ptilorhyncus*	21	330	1.69	G	clusters match subspecies	22
Neoaves	Apodidae	*Aerodramus fuciphagus*	8	1044	1.43	I	insufficient location data	23
Neoaves	Apodidae	*Aerodramus maximus*	16	405	0.72	I	insufficient location data	23
Neoaves	Apodidae	*Collocalia esculenta*	29	385	5.02	G	clusters match subspecies	24
Neoaves	Apodidae	*Collocalia linchi*	5	1142	2.98	G,S	clusters match subspecies	23,25
Neoaves	Coccyzidae	*Coccyzus americanus*	10	976	0.50	G,S	eastern, western US	26
Neoaves	Falconidae	*Falco tinnunculus*	8	998	0.71	G,S	clusters match subspecies	27
Neoaves	Gruidae	*Grus canadensis*	4	1143	0.93	G	clusters match subspecies	28
Neoaves	Laeidae	*Brachyramphus marmoratus*	14	1044	1.55	G,S	clusters match subspecies; split AOU 1998	29
Neoaves	Laridae	*Brachyramphus brevirostris*	7	330	1.07	G	Aleutian Islands, Gulf of Alaska; reference CR	30
Neoaves	Laridae	*Larus cachinnans*	26	300	0.69	G	clusters match subspecies; reference CR	31,32
Neoaves	Laridae	*Larus fuscus*	18	300	0.69	G,S	clusters match subspecies; reference CR	31,32
Neoaves	Opisthocomidae	*Opisthocomus hoazin*	6	961	3.57	I	insufficient location data	33

Neoaves	Picidae	*Dendrocopos major*	67	427	0.54	G,S	clusters match subspecies (Japan, mainland Asia)	34
Neoaves	Picidae	*Picoides tridactylus*	29	429	1.57	G,S	clusters match subspecies; split AOU	35
Neoaves	Podargidae	*Aegotheles archboldi*	4	527	0.57	N	no clusters, single collection location	36
Neoaves	Podargidae	*Aegotheles bennettii*	10	220	4.69	G,S	clusters match subspecies; split IOC	36
Neoaves	Podargidae	*Aegotheles insignis*	4	296	1.13	N	clusters not geographic	36
Neoaves	Procellaridae	*Calonectris diomedea*	41	300	0.67	G	clusters match subspecies	37
Neoaves	Procellariidae	*Puffinus lherminieri*	11	301	0.56	I	insufficient location data	38
Neoaves	Psittacidae	*Amazona ochrocephala*	13	656	1.71	G	clusters match subspecies	39
Neoaves	Psittacidae	*Aratinga auricapilla*	7	340	0.59	I	insufficient location data	40
Neoaves	Psittacidae	*Cacatua goffini*	5	327	0.67	I	includes captive birds	41
Neoaves	Psittacidae	*Cacatua haematuropygia*	5	325	0.80	I	includes captive birds	41
Neoaves	Psittacidae	*Cacatua sulphurea*	26	696	1.10	I	includes captive birds	41
Neoaves	Psittacidae	*Poicephalus robustus*	9	397	0.78	G	clusters match subspecies in GenBank records	
Neoaves	Psittacidae	*Probosciger aterrimus*	5	696	0.92	I	insufficient location data; reference not available	42
Neoaves	Psittacidae	*Psittacula krameri*	6	799	2.17	G	clusters match subspecies	43
Neoaves	Psittacidae	*Psittacus erithacus*	7	720	1.72	G,S	clusters match subspecies	44
Neoaves	Spheniscidae	*Eudyptula minor*	13	308	1.75	G,S	Australia, New Zealand	45
Neoaves	Strigidae	*Aegolius funereus*	6	300	0.82	G	Nearctic, Palearctic; reference COI	46
Neoaves	Strigidae	*Asio otus*	11	300	1.20	G	Nearctic, Palearctic; reference COI	46
Neoaves	Strigidae	*Bubo bubo*	24	292	0.71	G,S	clusters match subspecies	47
Neoaves	Strigidae	*Glaucidium brasilianum*	17	298	1.04	G,S	North America, South America	48
Neoaves	Strigidae	*Speotyto cunicularia*	7	296	2.45	G,S	North America, South America	49
Neoaves	Strigidae	*Strix aluco*	13	1040	0.74	I	insufficient location data	47
Neoaves	Strigidae	*Strix uralensis*	6	299	1.07	G	geographic clusters; reference CR	50
Neoaves	Sulidae	*Sula dactylatra*	5	450	1.00	G	clusters match subspecies	51
Neoaves	Sulidae	*Sula leucogaster*	5	450	0.80	G	clusters match subspecies	51
Neoaves	Trochilidae	*Metallura tyrianthina*	6	342	2.53	G	Ecuador, Bolivia	52
Neoaves	Tytonidae	*Tyto alba*	21	295	1.61	G,S	Nearctic, Palearctic; reference COI	46
Passeriformes	Corvidae	*Corvus corax*	86	249	1.25	G	California, Holarctic	53
Passeriformes	Corvidae	*Corvus cryptoleucus*	6	306	0.74	N	no clusters	53
Passeriformes	Corvidae	*Corvus macrorhynchos*	41	336	0.82	G	mainland Asia, Japan	54
Passeriformes	Fringillidae	*Basileuterus fulvicauda*	5	1142	4.03	G,S	multiple geographic clusters Central, South America	10
Passeriformes	Fringillidae	*Basileuterus rivularis*	4	1139	3.37	G,S	multiple geographic clusters Central, South America	10
Passeriformes	Fringillidae	*Cacicus cela*	5	901	1.89	G	clusters match subspecies	55
Passeriformes	Fringillidae	*Cacicus uropygialis*	5	918	4.12	G,S	clusters match subspecies	55
Passeriformes	Fringillidae	*Fringilla coelebs*	15	615	1.80	G	clusters match subspecies	56
Passeriformes	Fringillidae	*Geospiza scandens*	5	921	0.56	H	hybridization G. fortis	57,58
Passeriformes	Fringillidae	*Icterus dominicensis*	4	905	3.76	G,S	clusters match subspecies	59
Passeriformes	Fringillidae	*Loxia curvirostra*	7	1143	0.77	G	clusters match subspecies plus gene flow; reference CR	60
Passeriformes	Fringillidae	*Passerella iliaca*	11	386	1.41	G,S	clusters match subspecies	61

Passeriformes	Fringillidae	*Psarocolius angustifrons*	4	919	1.38	G	clusters match subspecies	62
Passeriformes	Fringillidae	*Psarocolius decumanus*	6	918	1.77	G	clusters match subspecies	62
Passeriformes	Fringillidae	*Tangara gyrola*	5	1142	2.57	G,S	Central America, South America	63
Passeriformes	Fringillidae	*Vermivora ruficapilla*	4	1140	1.48	G	eastern, western US	11
Passeriformes	Fringillidae	*Wilsonia pusilla*	33	315	2.31	G,S	eastern, western US	6
Passeriformes	Furnariidae	*Xiphorhynchus elegans*	56	900	1.20	G	clusters match subspecies	64
Passeriformes	Furnariidae	*Xiphorhynchus guttatus*	7	1000	3.18	G,S	clusters match subspecies	65
Passeriformes	Furnariidae	*Xiphorhynchus spixii*	24	928	1.24	G,S	Para, Belém endemic areas in Brazil; split IOC, SACC	64
Passeriformes	Hirundinidae	*Petrochelidon fulva*	11	921	0.73	G,S	clusters match subspecies	66
Passeriformes	Muscicapidae	*Alethe castanea*	10	1140	1.37	G	geographic clusters central, west Africa	67
Passeriformes	Muscicapidae	*Alethe poliocephala*	15	612	4.05	G	geographic clusters central, west Africa	67
Passeriformes	Muscicapidae	*Monticola sharpei*	6	1059	0.86	I	GenBank records differ from article	68
Passeriformes	Muscicapidae	*Sheppardia aurantithorax*	6	838	1.07	N	clusters not geographic	69
Passeriformes	Muscicapidae	*Sheppardia lowei*	4	717	2.16	G	Iringa/Moro, Uluguru Mts Tanzania	69
Passeriformes	Muscicapidae	*Turdus olivaceus*	4	671	0.77	G	clusters match subspecies	70
Passeriformes	Nectariniidae	*Nectarinia humbloti*	6	301	1.88	G	clusters match subspecies; split IOC	71
Passeriformes	Nectariniidae	*Nectarinia oritis*	22	566	1.14	G	clusters match geography	72
Passeriformes	Nectariniidae	*Nectarinia souimanga*	13	284	1.68	G,S	clusters match subspecies; split IOC	71
Passeriformes	Orthonychidae	*Orthonyx novaeguineae*	6	221	4.25	G,S	NW, SE New Guinea	73
Passeriformes	Paridae	*Parus caeruleus*	8	306	2.71	G,S	clusters match subspecies	74
Passeriformes	Paridae	*Parus montanus*	20	306	1.40	G	clusters match subspecies	75
Passeriformes	Passeridae	*Motacilla citreola*	6	424	0.53	G,S	eastern, western Palearctic	76
Passeriformes	Passeridae	*Motacilla flava*	22	413	3.28	G,S	multiple geographic clusters Palearctic	76
Passeriformes	Passeridae	*Passer montanus*	7	358	4.79	P	incl 2 prob pseudogenes (L76637, L76717)	77
Passeriformes	Pomastostomidae	*Pomatostomus temporalis*	15	282	2.09	G,S	north, south Australia	787
Passeriformes	Pycnonotidae	*Andropadus masukuensis*	8	282	4.52	G,S	clusters match subspecies; split IOC	79
Passeriformes	Regulidae	*Regulus regulus*	19	576	3.14	G	clusters match subspecies	80
Passeriformes	Rhinocryptidae	*Scytalopus latebricola*	4	281	1.60	I	dataset includes unique sequences only	81
Passeriformes	Sturnidae	*Toxostoma curvirostre*	66	398	1.38	G,S	clusters match subspecies	82
Passeriformes	Sturnidae	*Toxostoma lecontei*	5	431	2.00	G,S	clusters match subspecies	83
Passeriformes	Sylviidae	*Acrocephalus agricola*	7	480	1.53	G,S	clusters match subspecies	84
Passeriformes	Sylviidae	*Acrocephalus bistrigiceps*	6	480	0.88	G	Thailand, Ussuria	84
Passeriformes	Sylviidae	*Acrocephalus gracilirostris*	8	477	1.54	G	Kenya, South Africa	84
Passeriformes	Sylviidae	*Acrocephalus melanopogon*	10	1041	1.49	G	clusters match subspecies	84
Passeriformes	Sylviidae	*Acrocephalus newtoni*	5	306	0.52	N	clusters not geographic	85
Passeriformes	Sylviidae	*Acrocephalus scirpaceus*	8	1026	2.69	G,S	clusters match subspecies	84
Passeriformes	Sylviidae	*Cettia diphone*	6	1143	1.32	G,S	clusters match subspecies	86
Passeriformes	Sylviidae	*Garrulax canorus*	48	1143	1.64	G,S	clusters match subspecies	87
Passeriformes	Sylviidae	*Locustella pleskei*	6	1143	0.71	I	insufficient location data	
Passeriformes	Sylviidae	*Phylloscopus chloronotus*	14	442	2.23	G,S	west China, Nepal	88

Passeriformes	Sylviidae	*Phylloscopus collybita*	4	1027	1.36	G	clusters match subspecies	89
Passeriformes	Sylviidae	*Phylloscopus kansuensis*	5	442	0.90	I	insufficient location data	88
Passeriformes	Sylviidae	*Phylloscopus proregulus*	5	442	0.63	I	insufficient location data	88
Passeriformes	Sylviidae	*Phylloscopus yunnanensis*	5	442	0.95	I	insufficient location data	88
Passeriformes	Thamnophilidae	*Drymophila caudata*	4	372	2.20	G,S	clusters match subspecies	90
Passeriformes	Thamnophilidae	*Drymophila devillei*	11	371	1.42	G	northern Boliva, eastern Boliva/Brazil	91
Passeriformes	Thamnophilidae	*Formicivora rufa*	6	378	0.79	G,S	Boliva, Brazil	92
Passeriformes	Thamnophilidae	*Hypocnemis cantator*	6	362	4.62	G,S	Brazil, Bolivia; split SACC, IOC	93
Passeriformes	Troglodytidae	*Troglodytes troglodytes*	42	963	0.70	G,S	clusters match subspecies; split AOU	94
Passeriformes	Vireonidae	*Vireo gilvus*	4	273	1.71	G,S	clusters match subspecies; split Avibase	95
Passeriformes	Vireonidae	*Vireo solitarius*	11	273	1.12	G,S	clusters match subspecies; split AOU	95
