# Supplementary material for: DNA Barcoding Works in Practice but Not in (Neutral) Theory
Source: PLoS One. 2014 Jul 2;9(7):e100755. doi: 10.1371/journal.pone.0100755 (PMC4079456; doi:10.1371/journal.pone.0100755)
Supplement: Table S4 — Avian species with shared DNA barcode clusters. (PDF) [file pone.0100755.s010.pdf]

Table S4. Avian species with shared barcode clusters. Sets and pairs identified in four large series (48,77,127,128) are shown.

No. species in cluster	Species name 1	Species name 2	resolvable by barcode (Y/N), single sequence overlap (S), proposed lump (L)	hybridization (H), introgression (I), young species (Y)	Comments	References
2	*Aechmophorus occidentalis*	*A. clarkii*	N	-	no other information	
2	*Anas discors*	*A. cyanoptera*	N	H,I	hybridization; introgression 70 Ka	96
2	*Anas flavirostris*	*A. sibilatrix*	N	H,I	hybridization; share divergent lineages with other Anas spp	
3	*Anas rubripes*	*A. platyrhynchos, fulvigula*	N	H,I	hybridization	97-99
3	*Anser brachyrhynchus*	*A. erythropus, A. fabalis*	N	H,I	hybridization	100
2	*Basileuterus culicivorus*	*B. hypoleucus*	N,L	-	lump with hypoleucus see ref	5
2	*Carduelis flammea*	*C. hornemanni*	N,L	-	no differentiation by other mtDNA, microsatellite	101
2	*Celeus elegans*	*C. lugubris*	N	I	introgression see ref	102
2	*Chen caerulescens*	*C. rossii*	N	H,I	hybridization; share divergent CR lineages	100,103
2	*Corvus brachyrhynchos*	*C. caurinus*	N,L	-	no differentiation by other mtDNA loci	104,105
2	*Gymnopithys rufigula*	*G. leucaspis*	S	-	1 leucapsis in rufigula cluster	
2	*Herpsilochmus atricapillus*	*H. sellowi*	S	-	1 sellowi between 2 atricapillus clusters	
2	*Hylophilus ochraceiceps*	*H. semicinereus*	S	-	1 semicinereus matches 1 ochraceiceps; multiple ochraceiceps clusters	
2	*Junco hyemalis*	*J. phaeonotus*	N	Y	young species 10 Ka	106
8	*Larus argentatus*	*L. californicus, fuscus, glaucescens, glaucoides, hyperboreus, thayeri, occidentalis*	N	H,I	hybridization; introgression	31,32,107
2	*Leucophaeus atricilaa*	*L. pipixcan*	S	-	1 atricilla paraphyletic; pipixcan cluster distinct	

2	*Loxia curvirostra*	*L. pytyopsittacus*	N	Y	young species 10 Ka	108,109
3	*Muscisaxicola flavinucha*	*M. cinereus, rufivertex*	Y	-	2 diagnostic sites rufivertex; 1 diagnostic site flavinucha vs cinereus	110
2	*Pica hudsonia*	*Pica nuttalli*	Y	-	1 diagnostic site	111
2	*Picumnus pygmaeus*	*P. temminckii*	S	-	1 pygmaeus, differs 1.4% vs temminckii	
2	*Plectrophenax nivalis*	*P. hyperboreus*	N	H,I	hybridization, documented by AFLP; speciation 18K-73 Ka	112
2	*Quiscalis mexicanus*	*Q. major*	Y	-	1 diagnostic site; major is distinct clade within mexicanus	113
2	*Setophaga occidentalis*	*S. townsendi*	N	H,I	hybridization, Townsend's replacing Hermit	114
2	*Somateria spectabilis*	*S. mollissima*	N	H,I	hybridization; CR 2 clusters, 1 mixed	100
2	*Sphyrapicus nuchalis*	*S. ruber*	N	H,I	hybridization	115
8	*Sporophila bouvreil*	*S. cinnamomea, hypochroma, hypoxantha, palustris, ruficollis, zelichi*	N	H,I	hybridization, introgresssion	116
2	*Synallaxis gujanensis*	*S. rutilans*	S	-	1 gujanensis in rutilans cluster; others distant	
2	*Tachyeres patachonicus*	*T. pteneres*	S	-	1 patachonicus close to 2 pteneres	
2	*Thraupis palmarum*	*T. sayaca*	S	-	1 palmarum matches sayaca, others distant	
2	*Turdus albicollis*	*T. leucomelas*	N	-	no other information	
3	*Tympanuchus phasianellus*	*T. cupido, pallidicinctus*	N	H,I	hybridization; also recent speciation 10-80 Ka	100,117
2	*Venilornis passerinus*	*V. frontalis*	Y	-	1 diagnostic site	
2	*Zonotricha atricapilla*	*Z. leucophrys*	N	I	introgression; differ by AFLP	118
