# Supplementary material for: DNA Barcoding Works in Practice but Not in (Neutral) Theory
Source: PLoS One. 2014 Jul 2;9(7):e100755. doi: 10.1371/journal.pone.0100755 (PMC4079456; doi:10.1371/journal.pone.0100755)
Supplement: References S1 — (DOCX) [file pone.0100755.s011.docx]

SUPPORTING INFORMATION REFERENCES

1. Wenink PW, Baker AJ, Rosner H-U, Tilanus MGJ (1996) Global mitochondrial DNA phylogeography of holarctic breeding dunlins (*Calidris alpina*). Evolution 50: 318-330.

2. Hoglund J, Johansson T, Beintema A, Schekkerman H (2009) Phylogeography of the Black-tailed Godwit *Limosa limosa*: geographic substructuring revealed by mtDNA control region sequences. J Ornithol 150: 45-53.

3. Sangster G, Collinson JM, Crochet P-A, Knox AG, Parkin DT, et al. (2011) Taxonomic recommendations for British birds: Seventh report. Ibis 153: 883-892.

4. Hebert PDN, Stoeckle MY, Zemlak TS, Francis CM (2004) Identification of birds through DNA barcodes. PLOS Biol 2: e312.

5. Vilaca ST, Santos FR (2010) Biogeographic history of the species complex *Basileuterus culicivorus* (Aves, Parulidae) in the Neotropics. Mol Phylogenet Evol 57: 585-597.

6. Irwin DE, Irwin JH, Smith TB (2011) Genetic variation and seasonal migratory connectivity in Wilson's warblers (*Wilsonia pusilla*): species-level differences in nuclear DNA between western and eastern populations. Mol Ecol 20: 3102-3115.

7. Escalante P (2009) Evolutionary history of a prominent North American warbler clade: The *Oporornis-Geothlypis* complex. Mol Phylogenet Evol 53: 668-678.

8. Perez-Eman JL (2005) Molecular phylogenetics and biogeography of the Neotropical redstarts (*Myioborus*; Aves, Parulinae). Mol Phylogenet Evol 37: 511-528.

9. Lovette IJ, Perez-Eman JL, Sullivan JP, Banks RC, Fiorentino I, et al. (2010) A comprehensive multilocus phylogeny for the wood-warblers and a revised classification of the Parulidae (Aves). Mol Phylogenet Evol 57: 753-770.

10. Lovette IJ (2004) Molecular phylogeny and plumage signal evolution in a trans-Andean and circum-Amazonian avian species complex. Mol Phylogenet Evol 32: 512-523.

11. Lovette IJ, Clegg SM, Smith TB (2004) Limited utility of mtDNA markers for determining connectivity among breeding and overwintering locations in three neotropical migrant birds. Conservation Biol 18: 156-166.

12. Milot E, Gibbs HL, Hobson KA (2000) Phylogeography and genetic structure of northern populations of the Yellow Warbler (*Dendroica petechia*). Mol Ecol 9: 667-681.

13. Dabrowski A, Fraxer R, Confer JL, Lovette IJ (2005) Geographic variability in mitochondrial introgression among hybridizing populations of Golden-winged (*Vermivora chrysoptera*) and Blue-winged (*V. pinus*) Warblers. Conservation Genet 6: 843-853.

14. Ruegg KC, Hijmans RJ, Moritz C (2006) Climate change and the origin of migratory pathways in the Swainson's Thrush, *Catharus ustulatus.* J Biogeography 33: 1172-1182.

15. Topp CM, Pruett CL, McCracken KG, Winker K (2013) How migratory thrushes conquered northern North America: a comparative phylogeography approach. PeerJ 1: e206.

16. Alstrom P, Barnes KN, Olsson U, Barker FK, Bloomer P, et al. (2013) Multilocus phylogeny of the avian family Alaudidae (larks) reveals complex morphological evolution, non-monophyletic genera and hidden species diversity. Mol Phylogenet Evol 69: 1043-1056.

17. Dor R, Safran RJ, Sheldon FH, Winkler DW, Lovette IJ (2010) Phylogeny of the genus *Hirundo* and the Barn Swallow subspecies complex. Mol Phylogenet Evol 56: 409-418.

18. Kerr KCR, Lijtmaer DA, Barreira AS, Hebert PDN, Tubaro PL (2009) Probing evolutionary patterns in Neotropical birds through DNA barcodes. PLOS ONE 4: e4379.

19. Baker AJ, Daugherty CH, Colbourne R, McLennan JL (1995) Flightless brown kiwis of New Zealand possess extremely subdivided population structure and cryptic species like small mammals. Proc Natl Acad Sci USA 92: 8254-8258.

20. Paxinos EE, James HF, Olson SL, Sorenson MD, Jackson J, et al. (2002) mtDNA from fossils reveals a radiation of Hawaiian geese recently derived from the Canada goose (*Branta canadensis*). Proc Natl Acad Sci U S A 99: 1299-1404.

21. Luzhang R, Bei A, Backstrom N, Huaxing L, Longying W, et al. (2010) Phylogeographic structure and gene flow of Himalayan snowcock (*Tetraogallus himalayensis*). Animal Biol 60: 449-465.

22. Gamauf A, Haring E (2004) Molecular phylogeny and biogeography of Honey-buzzards (genera *Pernis* and *Henicopernis*). J Zool Syst Evol Res 42: 145-153.

23. Thomassen HA, Wiersema AT, de Bakker MA, de Knijff P, Hetebrij E, et al. (2003) A new phylogeny of swiftlets (Aves: Apodidae) based on cytochrome-b DNA. Mol Phylogenet Evol 29: 86-93.

24. Price JJ, Johnson KP, Clayton DH (2004a) The evolution of echolocation in swiftlets. J Avian Biol 35: 135-143.

25. Moyle RG, Hosner PA, Nais J, Lakim M, Sheldon FH (2008) Taxonomic status of the Kinabalu '*linchi*' swiftlet. Bull Brit Ornith Club 128: 94-100.

26. Pruett CL, Gibson DD, Winker K (2001) Molecular “cuckoo clock” suggests listing of western Yellow-billed Cuckoos may be warranted. Wilson Bull 113: 228-231.

27. Groombridge JJ, Jones CG, Bayes MK, van Zyl AJ, Carrillo J, et al. (2002) A molecular phylogeny of African kestrels with reference to divergence across the Indian Ocean. Mol Phylogenet Evol 25: 267-277.

28. Krajewski C, Fetzner JW Jr (1994) Phylogeny of cranes (Gruiformes: Gruidae) based on cytochrome-b DNA sequences. Auk 111: 351-364.

29. American Ornithologists' Union (1998) The American Ornithologists’ Union Check-List of North American birds, 7th edition. Lawrence: Allen Press. 829 p.

30. Birt TP, MacKinnon D, Piatt JF, Friesen VL (2011) Genetic differentiation of the Kittlitz's Murrelet in the Aleutian Islands and Gulf of Alaska. Marine Ornithol 39: 45-51.

31. Liebers D, Helbig AJ, de Knijff (2001) Genetic differentiation and phylogeography of gulls in the *Larus cachinnans-fuscus* group (Aves: Charadriiformes). Mol Ecol 10: 2447-2462.

32. Liebers D, deKnijff P, Helbig AJ (2004) The Herring Gull (*Larus argentatus*) complex is not a ring species. Proc Biol Sci 271: 893-901.

33. Avise JC, Nelson WS, Sibley CG (1994) Why one-kilobase sequences from mitochondrial DNA fail to solve the Hoatzin phylogenetic enigma. Mol Phylogenet Evol 3: 175-184.

34. Zink RM, Drovetski SV, Rohwer S (2002) Phylogeographic patterns in the Great Spotted Woodpecker *Dendrocopos major* across Eurasia. J Avian Biol. 33: 175-178.

35. Zink RM, Rohwer S, Drovetski S, Blackwell-Rago RC, Farrell SL (2002) Holarctic phylogeography and species limits of Three-Toed Woodpeckers. Condor 104: 167-170.

36. Dumbacher JP, Pratt TK, Fleischer RC (2003) Phylogeny of the owlet-nightjars (Aves: Aegothelidae) based on mitochondrial DNA sequence. Mol Phylogenet Evol 29: 540-549.

37. Gomez-Diaz E, Gonzalez-Solis J, Peinado MA, Page RD (2006) Phylogeography of the *Calonectris* shearwaters using molecular and morphometric data. Mol Phylogenet Evol 41: 322-332.

38. Austin JJ (2004) Molecular phylogenetics of *Puffinus* shearwaters: preliminary evidence from mitochondrial cytochrome b gene sequences. Mol Phylogenet Evol 6: 77-88.

39. Eberhard JR, Bermingham E (2004) Phylogeny and biogeography of the *Amazona ochrocephala* (Aves: Psittacidae) complex. Auk 121: 318-322.

40. Ribas CC, Miyaki CY (2004) Molecular systematics in *Aratinga* parakeets: species limits and historical biogeography in the '*solstitialis*' group, and the systematic position of Nandayus Nenday. Mol Phylogenet Evol 30: 663-675.

41. Christidis L, Boles W, eds. (2008) Systematics and taxonomy of Australian birds. Collingwood: CSIRO Publishing. 288 p.

42. Schliebusch I, Schliebusch G, Henning FW, Schottler B (2001) The systematic status of the sulphur-crested and the lesser sulphur-crested cockatoo. Papageien 5: 166-174.

43. Groombridge JJ, Jones CG, Nichols RA, Carlton M, Bruford MW (2004) Molecular phylogeny and morphological change in the *Psittacula* parakeets. Mol Phylogenet Evol 31: 96-108.

44. Melo M, O'Ryan C (2007) Genetic differentiation between Príncipe Island and mainland populations of the grey parrot (*Psittacus erithacus*), and implications for conservation. Mol Ecol 16: 1673-1685.

45. Banks JC, Cruickshank RH, Drayton GM, Paterson AM (2008) Few genetic differences between Victorian and Western Australian blue penguins, *Eudyptula minor*. New Zealand J Zool 35: 265-270.

46. Johnsen A, Rindal E, Ericson PGP, Zuccon D, Kerr KCR, et al. (2010) DNA barcoding of Scandinavian birds reveals divergent lineages in trans-Atlantic species. J Ornithol 151: 565-578.

47. Wink M, Heidrich P (1999) Molecular evolution and systematics of owls (Strigiformes). In: Konig C, Weick F, Beckin JH (eds.) Owls of the world. Sussex: Pica Press. pp. 39-57.

48. Proudfoot GA, Honeycutt RL, Slack RD (2006) Mitochondrial DNA variation and phylogeography of the Ferruginous Pygmy-Owl (*Glaucidium brasilianum*). Conserv Genet 7: 1-12.

49. Desmond MJ, Parsons TJ, Powers TO, Savidge JA (2001) An initial examination of mitochondrial DNA structure in Burrowing Owl populations. J Raptor Res 35: 274-281.

# 50. Hausknecht R, Jacobs S, Müller J, Zink R, Frey H, et al. (2014) Phylogeographic analysis and genetic cluster recognition for the conservation of Ural Owls (Strix uralensis) in Europe. J Ornithol 155: 121-134.

51. Steeves TE, Anderson DJ, McNally H, Kim MH (2003) Phylogeography of *Sula*: the role of physical barriers to gene flow in the diversification of tropical seabirds. J Avian Biol 34: 217-223.

52. Garcia-Moreno J, Arctander P, Fjeldsa J (1999) Strong diversification at the treeline among *Metallur*a hummingbirds. Auk 116: 702-711.

53. Omland KE, Tarr CL, Boarman WI, Marzluff JM, Fleischer RC (2000) Cryptic genetic variation and paraphyly in ravens. Proc Biol Sci 267: 2475-2482.

54. Kryukov A, Spiridonova L, Nakamura S, Haring E, Suzuki H (2012) Comparative phylogeography of two crow species: Jungle Crow *Corvus macrorhynchos* and Carrion Crow *Corvus corone*. Zool Sci 29: 484-492.

55. Price JJ, Lanyon SM (2004) Patterns of song evolution and sexual selection in the oropendolas and caciques. Behav Ecol 15: 485-497.

56. Marshall HD, Baker AJ (1998) Rates and patterns of mitochondrial DNA sequence evolution in Fringilline finches (*Fringilla* spp.) and the Greenfinch (*Carduelis chloris*). Mol Biol Evol 15: 638-646.

57. Sato A, O'hUigin C, Figueroa F, Grant PR, Grant BR, et al. (1999) Phylogeny of Darwin's finches as revealed by mtDNA sequences. Proc Natl Acad Sci U S A 96: 5101-5106.

58. Grant PR, Grant BR (2002) Unpredictable evolution in a 30-year study of Darwin's finches. Science 296: 707-711.

59. Omland KE, Lanyon SM, Fritz SJ (1999) A molecular phylogeny of the New World orioles (*Icterus*): The importance of dense taxon sampling. Mol Phylogenet Evol 12: 224-229.

60. Questiau S, Gielly L, Clouet M, Taberlet P (1999) Phylogeographical evidence of gene flow among Common Crossbill (*Loxia curvirostra*, Aves, Fringillidae) populations at the continental level. Heredity 83: 196-205.

61. Zink RM, Weckstein JD (2003) Recent evolutionary history of the Fox Sparrows (Genus: *Passerella*). Auk 120: 522–527.

62. Price JJ, Lanyon SM (2002) A robust phylogeny of the oropendolas: polyphyly revealed by mitochondrial sequence data. Auk 119: 335-348.

63. Burns KJ, Naoki K (2004) Molecular phylogenetics and biogeography of Neotropical tanagers in the genus *Tangara*. Mol Phylogenet Evol 32: 838-854.

64. Alexio A (2004) Historical diversification of a terra-firme forest bird superspecies: a phylogeographic perspective on the role of different hypotheses of Amazonian diversification. Evolution 58: 1303-1317.

65. Naka LN, Bechtoldt CL, Henriques LMP, Brumfield RT (2012) The role of physical barriers in the location of avian suture zones in the Guiana Shield, Northern Amazonia. American Naturalist 179: E115-E132.

66. Kirchman JJ, Whittingham LA, Sheldon FH (2000) Relationships among cave swallow populations (*Petrochelidon fulva*) determined by comparisons of microsatellite and cytochrome b data. Mol Phylogenet Evol 14: 107-121.

67. Beresford P (2003) Molecular systematics of *Alethe*, *Sheppardia* and some other African robins (Muscicapoidea). Ostrich 74: 58-73.

68. Goodman SM, Weigt LA (2009) The generic and species relationships of the reputed endemic Malagasy genus *Pseudocossyphus* (family Turdidae). Ostrich 73: 26-35.

69. Beresford P, Fjeldsa J, Kiure J (2004) A new species of Akalat (*Sheppardia*) narrowly endemic in the Eastern Arc of Tanzania. Auk 121: 23-34

70. Bowie RCK, Bloomer P, Clancey PA, Crowe TM (2003) The Karoo Thrush, *Turdus smithi* Bonaparte 1850, a southern African endemic. Ostrich 74: 1-7.

71. Warren BH, Bermingham E, Bowie RCK, Prys-Jones RP, Thebaud C (2003) Molecular phylogeography reveals island colonization history and diversification of western Indian Ocean sunbirds (*Nectarinia*: Nectariniidae). Mol Phylogenet Evol 29: 67-85.

72. Smith TB, Holder K, Girman D, O'Keefe K, Larison B, et al. (2000) Comparative avian phylogeography of Cameroon and equatorial Guinea mountains: implications for conservation. Mol Ecol 9: 1505-1516.

73. Joseph L, Silkas B, Alpers D, Schodde R (2001) Molecular systematics and phylogeography of New Guinean logrunners (Orthonychidae). Emu 101: 273–280.

74. Dietzen C, Garcia-del-Rey E, Castro GD, Wink M (2008) Phylogeography of the blue tit (*Parus teneriffae*-group) on the Canary Islands based on mitochondrial DNA sequence data and morphometrics. J Ornithol 149: 1-12.

75. Salzburger W, Martens J, Nazarenko AA, Sun Y-H, Dallinger R, et al. (2002) Phylogeography of the Eurasian Willow Tit (*Parus montanus*) based on DNA sequences of the mitochondrial cytochrome b gene. Mol Phylogenet Evol 24: 26-34.

76. Pavlova A, Zink RM, Drovetski SV, Red'Kin, Rohwer S (2003) Phylogeographic patterns in *Motacilla flava* and *Motacilla citreola*: species limits and population history. Auk 120: 744-758.

77. Allende LM, Rubio I, Ruiz-Del-Valle V, Guillen J, Martinez-Laso J, et al. A (2001) The Old World sparrows (genus *Passer*) phylogeography and their relative abundance of mtDNA pseudogenes. J Mol Evol 53: 144-154.

78. Edwards SV, Wilson AC (1990) Phylogenetically informative length polymorphism and sequence variability in mitochondrial DNA of Australian songbirds (*Pomatostomus*). Genetics 126: 695-711.

79. Roy MS (1997) Recent diversification in African greenbuls (Pycnonotidae: *Andropadus*) supports a montane speciation model. Proc Biol Sci 264: 1337-1344.

80. Packert M, Martens J, Kosuch J, Nazarenko AA, Veith (2003) Phylogenetic signal in the song of crests and kinglets (Aves: *Regulus*). Evolution. 2003 57: 616-629.

81. Arctander P, Fjeldsa J (1994) Andean tapaculos of the genus *Scytalopus* (Aves, Rhinocryptidae): a study of speciation using DNA sequence data. In: Loeschcke V, Tomiuk J, Jain SK (eds.) Conservation Genetics. Basel: Birkhauser Verlag. pp. 205-225.

82. Rojas-Soto OR, de Los Monteros AE, Zink RM (2007) Phylogeography and patterns of differentiation in the Curve-billed Thrasher. Condor 109: 456-463.

83. Zink RM, Blackwell RC (1997) Species limits in the LeConte's Thrasher. Condor 99: 132-138.

84. Leisler B, Heidrich P, Schulze-Hagen K, Wink M (1997) Taxonomy and phylogeny of reed warblers (genus *Acrocephalus*) based on mtDNA sequences and morphology. J Ornithol 138: 469-496

85. Goodman SM, Tello JG, Langrand O (2000) Patterns of morphological and molecular variation in *Acrocephalus newtoni* on Madagascar. Ostrich 71: 367-370.

86. Alstrom P, Hohna S, Gelang M, Ericson PGP, Olsson U (2011) Non-monophyly and intricate morphological evolution within the avian family Cettiidae revealed by multilocus analysis of a taxonomically densely sampled dataset. BMC Evol Biol 11: 352.

87. Li S-H, Li J-W, Han L-X, Yao C-T, Shi H, et al. (2006) Species delimitation in the Hwamei *Garrulax canorus*. Ibis 148: 698–706.

88. Martens J, Tietze DT, Eck S, Veith M (2004) Radiation and species limits in the Asian Pallas’s warbler complex (*Phylloscopus proregulus s.l.*). J Ornithol 145: 206-222.

89. Helbig AJ Martens J, Seibold I, Henning F, Schottler, Wink M (1996) Phylogeny and species limits in the Palearctic chiffchaff *Phylloscopus collybita* complex: mitochondrial genetic differentiation and bioacoustic evidence. Ibis 138: 650-666.

90. Isler ML, Cuervo AM, Bravo GA, Brumfield RT (2012) An integrative approach to species-level systematics reveals the depth of diversification in an Andean thamnophilid, the Long-Tailed Antbird. Condor 114: 571-583.

91. Bates JM, Hackett SJ, Goerck JM (2000) High levels of mitochondrial DNA differentiation in two lineages of small antbirds (*Drymophila* and *Hypocnemis*). Auk 116: 1093-1106.

92. Bates JM, Tello JG, da Silva JMC (2003) Initial assessment of genetic diversity in ten bird species of South American Cerrado. Studies Neotrop Fauna Environ 38: 87-94.

93. Isler ML, Isler PR, Whitney BM, Yasukawa (2007) Species limits in Antbirds (Thamnophilidae): the Warbling Antbird (*Hypocnemis cantator*) complex. Auk 124: 11-28.

94. Chesser RT, Banks RC, Barker FK, Cicero C, Dunn JL, et al. (2010) Fifty-first supplement to the American Ornithologists' Union Check-List of North American Birds. Auk 127: 726-744.

95. Murray BW, McGillivray B, Barlow JC, Beech RN, Strobeck C (1994) The use of cytochrome b sequence variation in estimation of phylogeny in the Vireonidae. Condor 96: 1037-1045.

96. Wilson RE, Eaton MD, Sonsthagen SA, Peters JL, Johnson KP, et al. (2011) Speciation, subspecies divergence, and paraphyly in the Cinnamon Teal and Blue-winged Teal. Condor 113: 747-761.

97. Kulikova IV, Drovetski SV, Gibson DD, Harrigan RJ, Rohwer S, et al. (2005) Phylogeography of the Mallard (*Anas platyrhynchos*): hybridization, dispersal, and lineage sorting contribute to complex geographic structure. Auk 122: 949-965.

98. Mank JE, Carlson JE, Brittingham MC (2004) A century of hybridization: decreasing genetic distance between American black ducks and mallards. Conservation Genet 5: 395-403.

99. McCracken KG, Johnson WP, Sheldon FH (2001) Molecular population genetics, phylogeography, and conservation biology of the mottled duck (*Anas fulvigula*). Conservation Genet 2: 87-102.

100. McCarthy EM (2006) Handbook of avian hybrids of the world. New York: Oxford University Press. 601 pp.

101. Martinsen G, Wennerberg L, Lifjeld JT (2008) Low support for separate species within the redpoll complex (*Carduelis flammea–hornemanni–cabaret*) from analyses of mtDNA and microsatellite markers. Mol Phylogenet Evol 47: 1005-1017.

102. Benz BW, Robbins MB (2011) Molecular phylogenetics, vocalizations, and species limits in *Celeus* woodpeckers (Aves: Picidae). Mol Phylogenet Evol 61: 29-44.

103. Weckstein JD, Afton AD, Zink RM, Alisauskas RT (2002) Hybridization and population subdivision within and between Ross's Geese and Lesser Snow Geese: a molecular perspective. Condor 104: 432-436.

104. Johnston DW (1961) The biosystematics of American Crows. Seattle: University of Washington Press. 119 p.

105. Haring E, Daeubl B, Pinsker W, Kryukov A, Gamauf A (2012) Genetic divergences and intraspecific variation in corvids of the genus *Corvus* (Aves: Passeriformes: Corvidae) - a first survey based on museum specimens. J Zool Syst Evol Res 50: 230-246.

106. Mila B, McCormack JE, Castaneda G, Wayne RK, Smith TB (2007) Recent postglacial range expansion drives the rapid diversification of a songbird lineage in the genus *Junco.* Proc Biol Sci 274: 2653-2660.

107. Sonsthagen SA, Chesser RT, Bell DA, Dove CJ (2012) Hybridization among arctic white-headed gulls (*Larus* ssp.) obscures the genetic legacy of the Pleistocene. Ecol Evol 2: 1278-1295.

108. Piertney SB, Summers R, Marquiss M (2011) Microsatellite and mitochondrial DNA homogeneity among phenotypically diverse crossbill taxa in the UK. Proc Biol Sci 268: 1511-1517. ‎

109. Bjorklund M, Alonso D, Edelaar P (2013) The genetic structure of crossbills suggests rapid diversification with little niche conservatism. Biol J Linnean Soc 109: 908-922.

110. Chesser RT (2000) Evolution in the high Andes: the phylogenetics of *Muscisaxicola* Ground-Tyrants. Mol Phylogenet Evol 15: 369-380.

111. Lee S-I, Parr CS, Hwang Y, Mindell DP, Choe JC (2003) Phylogeny of magpies (genus *Pica*) inferred from mtDNA data. Mol Phylogenet Evol 29: 250-257.

112. Maley JM, Winker K (2010) Diversification at high latitudes: speciation of buntings in the genus *Plectrophenax* inferred from mitochondrial and nuclear markers. Mol Ecol 19: 785-797.

113. DaCosta JM, Wehtje W, Klicka J (2008) Historic genetic structuring and paraphyly within the Great-tailed Grackle. Condor 110: 170-177.

114. Krosby M, Rohwer S (2009) A 2000  km genetic wake yields evidence for northern glacial refugia and hybrid zone movement in a pair of songbirds. Proc Biol Sci 276: 615-621.

115. Johnson NK, Johnson CB (1985) Speciation in sapsuckers (*Sphyrapicus*): II. Sympatry, hybridization and mate preference in *S. ruber daggetti* and *S. nuchalis*. Auk 102: 1-15.

116. Campagna L, Benites P, Lougheed SC, Lijtmaer DA, Di Giacomo AS, et al. (2012) Rapid phenotypic evolution during incipient speciation in a continental avian radiation. Proc Biol Sci 279: 1847-1856.

117. Spaulding AW, Mock KE, Schroeder MA, Warheit KI (2006) Recent range expansion and divergence among North American Prairie Grouse. Mol Ecol 15: 2317-2332.

118. Weckstein JD, Zink RM, Blackwell-Rago RC, Nelson DA (2001) Anomalous variation in mitochondrial genomes of White-crowned (*Zonotrichia leucophrys*) and Golden-crowned (*Z. atricapilla*) Sparrows: pseudogenes, hybridization, or incomplete lineage sorting. Auk 118: 231-236.
